# Ultra-resilient multi-layer fluorinated diamond like carbon hydrophobic surfaces

**DOI:** 10.1038/s41467-023-40229-6

**Published:** 2023-08-14

**Authors:** Muhammad Jahidul Hoque, Longnan Li, Jingcheng Ma, Hyeongyun Cha, Soumyadip Sett, Xiao Yan, Kazi Fazle Rabbi, Jin Yao Ho, Siavash Khodakarami, Jason Suwala, Wentao Yang, Omid Mohammadmoradi, Gozde Ozaydin Ince, Nenad Miljkovic

**Affiliations:** 1 Department of Mechanical Science and Engineering, University of Illinois, Urbana, IL USA; 2 Oerlikon Balzers Coating, Schaumburg, IL USA; 3 Department of Materials Science and Nanoengineering, Sabanci University, Istanbul, Turkey; 4 Sabanci University Nanotechnology Research and Application Center, Istanbul, Turkey; 5 Materials Research Laboratory, University of Illinois, Urbana, IL USA; 6 Department of Electrical and Computer Engineering, University of Illinois, Urbana, IL USA; 7 International Institute for Carbon Neutral Energy Research (WPI-I2CNER), Kyushu University, 744 Motooka, Nishi-ku, Fukuoka 819-0395 Japan; 8 Present Address: GPL Photonics Laboratory, State Key Laboratory of Luminescence and Applications, Changchun Institute of Optics, Fine Mechanics and Physics, Chinese Academy of Sciences, Changchun, Jilin 130033 P. R. China; 9 Present Address: Massachusetts Institute of Technology, 77 Massachusetts Avenue, Cambridge, MA USA

**Keywords:** Materials for energy and catalysis, Energy science and technology

## Abstract

Seventy percent of global electricity is generated by steam-cycle power plants. A hydrophobic condenser surface within these plants could boost overall cycle efficiency by 2%. In 2022, this enhancement equates to an additional electrical power generation of 1000 TWh annually, or 83% of the global solar electricity production. Furthermore, this efficiency increase reduces CO_2_ emissions by 460 million tons /year with a decreased use of 2 trillion gallons of cooling water per year. However, the main challenge with hydrophobic surfaces is their poor durability. Here, we show that solid microscale-thick fluorinated diamond-like carbon (F-DLC) possesses mechanical and thermal properties that ensure durability in moist, abrasive, and thermally harsh conditions. The F-DLC coating achieves this without relying on atmospheric interactions, infused lubricants, self-healing strategies, or sacrificial surface designs. Through tailored substrate adhesion and multilayer deposition, we develop a pinhole-free F-DLC coating with low surface energy and comparable Young’s modulus to metals. In a three-year steam condensation experiment, the F-DLC coating maintains hydrophobicity, resulting in sustained and improved dropwise condensation on multiple metallic substrates. Our findings provide a promising solution to hydrophobic material fragility and can enhance the sustainability of renewable and non-renewable energy sources.

## Abstract (web summary)

Hydrophobic condenser surfaces can improve power plant efficiency, but they suffer from poor durability. Here the authors developed a durable fluorinated diamond-like carbon coating that improved dropwise condensation and maintained hydrophobicity over three years of pure steam condensation on multiple metallic substrates.

## Introduction

Over the last two decades, the share of global electricity generation provided by solar photovoltaic (PV) and wind energy installations has risen from 0.2% to 10.1%^1^. However, as much as 70% of global electrical power generation is generated using traditional steam power cycles, which use either a fossil fuel or nuclear fission as the energy source. Therefore, increasing the overall efficiency of steam power plants has a disproportional impact on sustainability, global energy production, and the carbon footprint of developing and developed nations. Among the several strategies to increase power plant efficiency and sustainability, such as deploying supercritical steam cycles^2^ and optimizing power station infrastructure^3^, maximizing the steam condenser heat transfer represents the simplest, well known, and promising path to consider. Doing so has been shown to result in a potential 2% net increase in power plant energy efficiency^3^. If realized today, this efficiency improvement would increase global annual electricity generation by approximately 1000 TWh^1^, equivalent to matching 83% of the total electricity produced by all global PV installations with no additional increase in carbon emissions.

A key process governing the efficiency of the steam power cycle is the condensation occurring on a metallic shell-and-tube condenser. The hydrophilicity of these metal tube surfaces results in the condensate forming a water film on the condenser surface, termed filmwise condensation, which slows thermal transport^4^. However, it is well established that the filmwise condensation rate can be enhanced by modifying the condenser surface with a thin hydrophobic coating. On hydrophobic surfaces, steam condensate forms discrete droplets that easily shed due to dropwise condensation resulting in up to 2000% higher condensation heat transfer rates. This enhanced rate translates into a potential 2% net increase in power plant energy efficiency stemming from the ability to run the condenser at a lower steam pressure, and to extract more enthalpy from the steam working fluid^5,6^. Furthermore, use of hydrophobic surfaces in power plants also provides valuable corrosion and erosion protection for condenser tubing^7^, increasing up-time for power plant condensers, and decreasing power plant operating expense.

Designing a suitable hydrophobic coating is the main challenge to deploying dropwise condensation in real power systems. To overcome this challenge, five coating requirements need to be met simultaneously (Supplementary Fig. 1). (1) The coating needs to be fabricated scalably and must be applicable to a variety of potential substrate materials. Typical condensers in a power station are fabricated from different metals or metal alloys depending on the region, and they are relatively large (~10 m) often containing thousands of tubes in a bundle. (2) The coating needs to be thin (<5 μm) so that the coating parasitic thermal resistance does not off-set the benefit of high dropwise condensation heat transfer^8^. (3) The coating needs to resist delamination caused by the condensate capillary force. Specifically, condensation-induced blistering, which is the most direct coating degradation mechanism, enables condensate penetration between the interface of the coating through surface defects. This penetration causes localized pressure, and gradually results in complete coating delamination^9^. (4) The coating must be thermally stable at elevated temperature environments. (5) The coating needs to resist mechanical abrasion during condenser fabrication, assembly, maintenance, and operation. Classical artificial hydrophobic materials such as low surface energy polymers cannot meet these requirements and cannot be utilized due to their low elastic modulus, and poor thermal and mechanical stability.

To craft durable hydrophobic coatings, past strategies have generally focused on optimizing the coating geometric and structural design. By doing so, these have demonstrated that coating robustness can be enhanced by surface structures which act as a protective or sacrificial armor^10–12^. However, these hierarchical protective structures make the overall coating thick (>10 μm), not suitable for condenser applications. Thus far, little progress has been made to enhance the coating intrinsic properties to overcome the durability challenge.

Interestingly, a coating material which can potentially achieve the required coating intrinsic properties for steam condensation applications is diamond-like carbon (DLC). The intrinsic properties of DLC thin films give them the desired mechanical and thermal stability, which are strongly related to the hybridization of sp^2^ and sp^3^ carbon bonding^13–15^. Moreover, the abrasion resistance and corresponding interfacial adhesion of DLC coatings can be enhanced by adapting multilayer designs^16,17^. However, the utilization of such structures for phase-change heat transfer has been under-explored. Specifically, a literature review (included in Supplementary Note 2) reveals that the majority of the past studies have explored tribological, medical, mechanical, wettability, and dielectric property improvement with a focus on altering the fabrication method, recipe modification, and doping. Limited focus has been placed on substrate versatility, or the long-term sustainability of enhanced properties (see Supplementary Table 1). Furthermore, no past study has synergistically considered the crucial parameters required to achieve robust hydrophobic coating design or demonstrated continual dropwise condensation under sustained long-term (>3 year) steam exposure.

Here, we develop a hydrophobic coating synthesis and application approach using multilayer fluorinated diamond-like carbon (F-DLC) that meets all five requirements to enable sustained dropwise condensation of steam. By using scalable co-deposition of a short-chain fluorocarbon with well-established DLC (a-C:H, amorphous hydrogenated carbon films), we demonstrate a hydrophobic coating having surface energy characteristics of non-polar polymers, with a high Young’s modulus approaching that of pure metals. We demonstrate the versatility of F-DLC on a wide range of substrates including crystalline, non-crystalline and common engineering metals, all showing similar surface energy after coating. Multilayer fluorinated diamond-like carbon not only demonstrates enhanced dropwise condensation heat transfer, but also durability in moist environments for a period of more than three years. Characterization of the compatibility of multilayer F-DLC in elevated temperature environments exceeding 300 °C and sustainability after 5000 mechanical abrasion cycles demonstrates thermomechanical resiliency. The outcomes of our work not only develop a low surface energy coating capable of implementation with many potential applications, they overcome barriers to generating hydrophobic surfaces which are resilient to harsh thermomechanical environments in energy production infrastructure.

## Results

### Hydrophobic coating design strategy

The rational design of the multilayer F-DLC coating (Supplementary Fig. 2ii) was guided by our physics-based understanding of condensation-induced blistering. The quantitative parameter that describes blistering, $\varOmega$, demonstrates that delamination of a hydrophobic coating will occur if $\varOmega \, > \, 1$^9^. Specifically, the blistering parameter $\varOmega$ is governed by the pinhole size, ${R}_{{{{{{\rm{d}}}}}}}$, the base radius of the pinhole-adjunct delaminated region, ${R}_{b0}$, the liquid-vapor surface tension of the working fluid (water) $\gamma$, as well as the coating intrinsic properties including its wet adhesion $G$, Young’s modulus $E$ and coating thickness $h$:

1 $$\varOmega=\left(\frac{{1.04R}_{{{{{{\rm{b}}}}}}0}}{{R}_{{{{{{\rm{d}}}}}}}}\right){\left(\frac{{\gamma }^{4}}{E{G}^{3}h}\right)}^{\frac{1}{4}}.$$

For a typical 100-nm-thick fluoropolymer deposited on a smooth metal substrate (${R}_{{{{{{\rm{b}}}}}}0}\,\approx \,5{R}_{{{{{{\rm{d}}}}}}}$, $E\,\approx \,1{{{{{\rm{GPa}}}}}}$, $G\,\approx \,10{{{{{\rm{mJ}}}}}}.{{{{{{\rm{m}}}}}}}^{-2}$), $\varOmega \,\approx \,3.6$. Hence, polymers are unable to prevent delamination, unless their thicknesses exceed 10 μm where $\varOmega \,\approx \,1$. Our multilayer F-DLC coating decreases $\varOmega$ by using several synergistic approaches. By co-depositing short-chain perfluorinated compounds (PFCs) with the top DLC (a-C:H) surface (f-DLC), we enable high Young’s modulus of $E$ = 78 GPa and low surface energy of ~24 ${{{{{\rm{mJ}}}}}}.{{{{{{\rm{m}}}}}}}^{-2}$. By deploying a well-established titanium (Ti) bonding layer, we enable an interfacial toughness of ~10 J m^−2^. Utilizing our Ti-DLC-f-DLC multilayer, we eliminate pinholes, ensuring $\varOmega \, <$ 4.2 × 10^−3^ via the design of a 1-μm-thick multilayer F-DLC coating.

While the Ti-DLC-f-DLC multilayer focuses primarily on blistering and delamination, satisfying abrasion resistance and high temperature stability requires further stack modification. In addition to the aforementioned three-layer design, we included an additional layer composed of co-deposited DLC and silica (a-C:H:Si:O, amorphous hydrogenated carbon films containing silicon and oxygen) between the DLC layer and the Ti adhesion layer, which we term DLN. The added DLN layer is well-adapted in conventional DLC multilayers to: (1) enhance adhesion with the Ti layer by silica infusion, (2) provide good thermal stability and act as a stress reliever, and (3) further decrease the pinhole density. As a result, the multilayer F-DLC coating designed here not only promises reliable adhesion between the coating and a variety of arbitrary substrates, it also decreases interfacial stresses from abrasion or thermal expansion by increasing the numbers of layered interfaces. We note again that f-DLC represents the top fluorinated DLC layer while F-DLC represents the entire multilayer coating including the Ti, DLN, DLC, and f-DLC layers.

### Nanofabrication and structure properties

The F-DLC fabrication process is a rationally-developed and optimized multilayer film comprised of a primary adhesion layer deposited via physical vapor deposition (PVD) sputtering followed by plasma assisted physical vapor deposition (PACVD) to build the remaining three layers. We attempted multiple failed coating designs and fabrication iterations prior to successfully optimizing the described F-DLC recipe. These included failed attempts to use traditional DLC and surface modified DLC for condensation applications (see Supplementary Note 3). The failed attempts revealed that traditional DLC and surface modified DLC coatings have high contact angle hysteresis (difference between advancing and receding angles) leading to filmwise condensation (see Supplementary Table 2). These failed attempts, and the physics-based understanding stemming from them, guided the design of the multilayer F-DLC coating. The PACVD coating is done in a vacuum chamber at 250 °C, maintaining localized filaments to assist in homogenous plasma conditions throughout the vessel. Hydro-carbonated DLN gases which give the elemental structure were introduced in the system which was deposited on the Ti-coated substrate. Then a fluorine precursor as a liquid form is introduced to the system which was vaporized and co-deposited with the DLC layer at the end of the deposition process. In our study, a 0.29-µm-thick layer of PVD sputtered Ti on the substrate ensures strong adhesion between the subsequent multilayer deposition and the substrate. The Ti PVD process is followed by the deposition of a 0.30-µm-thick DLN (a-C:H:Si:O) intermediate layer, a 0.51-µm-thick DLC (a-C:H) layer, and finally a 0.50-µm-thick fluorinated DLC (f-DLC) layer (a-C:H:O:Si:F, amorphous hydrogenated carbon films containing silicon, oxygen and fluorine), in sequential order.

**Fig. 1:**
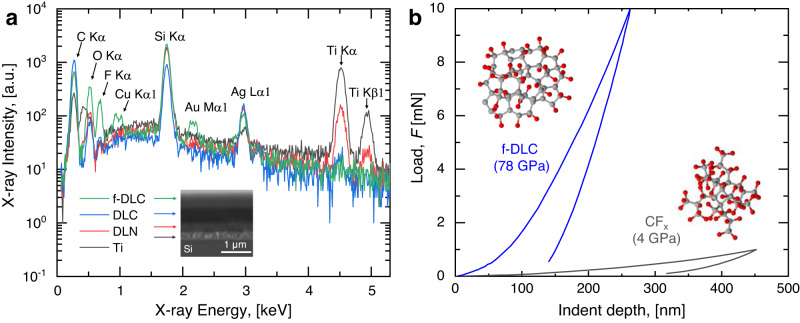
Design and structure of F-DLC. **a** Layer structure and chemistry of F-DLC obtained from EDS. Peaks of Au and Cu stem from impurities present in the EDS chamber. Inset: cross-sectional SEM image of F-DLC deposited on a polished silicon wafer. **b** Load-depth curve showing F-DLC has a~ 20× higher Young’s modulus when compared to amorphous C–F materials. Inset: schematics of the atomic structures of the top f-DLC coating in the F-DLC stack and the amorphous C–F coating.

Cross-sectional SEM of the coating shows a total thickness of ~1.65 ± 0.05 µm (Fig. 1a inset). As shown in Fig. 1a, energy-dispersive X-ray spectroscopy (EDS) analysis of each layer ensures the presence of the required respective components. The multilayer F-DLC film not only generates a pinhole-free coating but also improves the mechanical resiliency of the surface.

To evaluate and compare the elastic properties, nanoindentation (Hysitron TI 950 TriboIndenter) was performed on both the F-DLC multilayer coating and a control sample consisting of an amorphous carbon-fluorine (CFx) film of equivalent thickness. The depth of the indent was controlled by the applying force from the indenting tip (Berkovich TI-0039 standard tip, Bruker) to the samples. As shown in Fig. 1b, the F-DLC coating shows a ~20× higher Young’s modulus when compared to amorphous carbon-fluorine materials. Additional details of the material fabrication, cross-sectional SEM, and EDS analysis are included in the “Methods” section.

### Surface characterization and wettability

**Fig. 2:**
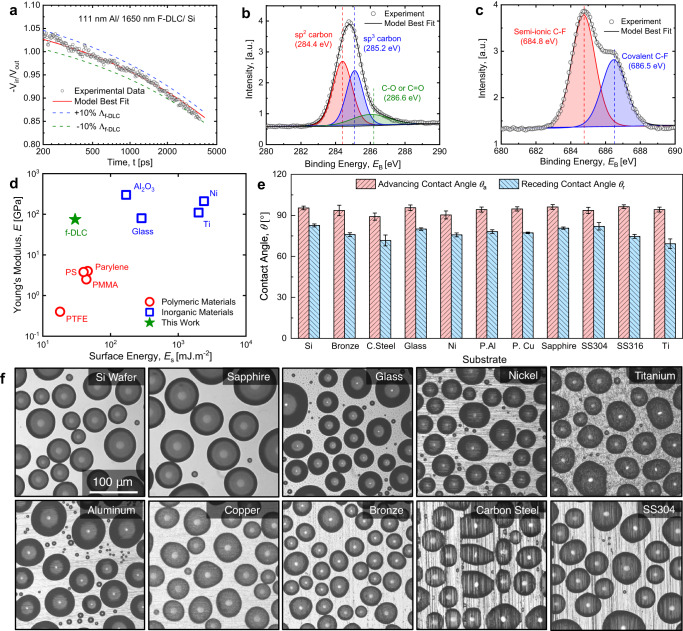
Surface characterization, wettability, and versatility of F-DLC coatings. **a** TDTR thermal reflectivity as a function of the time delay Δ*t* between pump and probe pulses on the F-DLC coating. The measured thermal conductivity was *k* = 0.46 ± 0.05 W m^−1^ K^−1^. The TDTR sample consisted of a 111 nm sputtered Al layer on a 1650 nm F-DLC multilayer stack. **b** X-ray photoelectron spectroscopy (XPS) of the C1s peak demonstrating the three components consisting of *sp*^3^ (C–C) bonds at 285.2 eV, *sp*^2^ (C=C) bonds at 284.4 eV, and C–O or C=O bonds at 286.6 eV. **c** The XPS F1s spectrum showed the highest amount of fluorine atoms are bonded with carbon by covalent and semi-ionic C–F bonds. **d** Surface energy and Young’s modulus of different commonly used engineering materials, showing that F-DLC combines the merit of both low surface energy and high mechanical modulus. **e** Measured apparent advancing and receding contact angles of DI water droplets on a variety of substrates coated with F-DLC. Here, the error bars indicate the standard deviation, which was determined based on three spatially independent measurements conducted for each data point. **f** Optical microscopy top-view images of atmospheric water vapor condensation on the different F-DLC coated substrates, showing the substrate versatility with similar hydrophobicity of the F-DLC coating.

The thermal conductivity of the fabricated multilayer F-DLC sample was measured using time-domain thermoreflectance (TDTR). Details of TDTR measurement are discussed in the “Methods” section. Figure 2a shows the thermal reflectivity change as a function of the time delay $\varDelta t$ between the pump and probe pulses for the test sample, yielding a thermal conductivity of the coating of $k$ = 0.46 ± 0.05 W m^−1^ K^−1^ by assuming a volumetric heat capacity consistent with previous measurements on DLC materials, $C=$ 2.5 J cm^−3^ K^−1 ^^18^. Due to the limited thermal penetration depth of the TDTR measurement, $d=\sqrt{k/\pi {Cf}} \sim$100 nm, we measure the thermal conductivity of the top fluorinated f-DLC layer. As the top f-DLC layer is the most amorphous among all layers due to the fluorination process, it has the lowest thermal conductivity of all layers, ensuring that our measurement represents the lower-bound thermal conductivity of the entire multilayer coating.

The mechanical properties of DLC-like materials are strongly related to the hybridization of *sp*^2^ and *sp*^3^ carbon bonding. To quantitatively measure the carbon composition of the top layer, we used X-ray photoelectron spectroscopy (XPS, Fig. 2b). The main peak of the C1s spectrum was decomposed into three components: the *sp*^3^ (C–C) bond at 285.2 eV, the *sp*^2^ (C=C) bond at 284.4 eV and the C–O or C=O bonds at 286.6 eV. We did not observe noticeable amounts of C-Si bonding, which should be located at ~283 eV. The area of the decomposed peaks indicates that relative content of the *sp*^3^ and *sp*^2^ bonds is 47 ± 10% and 35 ± 10%, respectively, where the uncertainties were calculated from the uncertainty of the sp^2^ peak position (0.1 eV). The relative content result indicates that although the top layer contains fluorine, the carbon bonding structure does not deviate appreciably from typical DLC materials and maintains a high Young’s modulus and hardness (Fig. 1a). The F1s spectrum showed that most fluorine atoms are bonded with carbon by covalent and semi-ionic C–F bonds. Additional details of the XPS analysis are included in the Methods Section.

The surface energy (${E}_{{{{{{\rm{s}}}}}}}$) of the F-DLC coating was measured by probing the advancing contact angle of deionized (DI) water and diiodomethane (Dii) on the F-DLC surface, which yielded ${E}_{s}$ = 24 ± 1 mJ m^−2^. Details of the surface energy measurement can be found in the “Methods” section^19^. Although the surface energy is 20% higher when compared to Teflon-like materials having surface energy of ~20 mJ m^−2^, the F-DLC coating maintains a ~20× higher Young’s modulus. Figure 2d shows the surface energy and Young’s modulus of many commonly used engineering materials, demonstrating that F-DLC combines the merits of both low surface energy and high mechanical modulus.

To demonstrate versatility, we deposited F-DLC on a variety of substrates, showing substrate-independent wetting. The wettability of the F-DLC coated substrates were determined by performing water contact angle measurements using a microgoniometer (MCA-3, Kyowa Interface Science). The apparent advancing contact angle on an F-DLC coated smooth silicon wafer (University Wafer) is, ${\theta }_{{{{{{\rm{a}}}}}}}=$97.5 ± 1.0° (Fig. 2e). Figure 2e shows the apparent advancing (${\theta }_{{{{{{\rm{a}}}}}}}$) and receding (${\theta }_{{{{{{\rm{r}}}}}}}$) contact angles of water on a wide variety of F-DLC coated substrates. A slight variation in the apparent advancing contact angle (${\theta }_{{{{{{\rm{a}}}}}}}$), and contact angle hysteresis ($\Delta \theta={\theta }_{{{{{{\rm{a}}}}}}}-{\theta }_{{{{{{\rm{r}}}}}}}$) among the substrates occurred due to the variability in topological homogeneity (roughness) of the surfaces^20^. Surface roughness homogeneity also affects the droplet distribution and droplet shapes during water droplet condensation. Figure 2f shows top-view optical microscopy images of atmospheric water vapor condensation on the different F-DLC coated substrates demonstrating spherical droplet morphologies with highly mobile contact lines, key to attaining high quality hydrophobicity. Additional details of the condensation experiment can be found in the “Methods” section.

### Steam condensation heat transfer performance

**Fig. 3:**
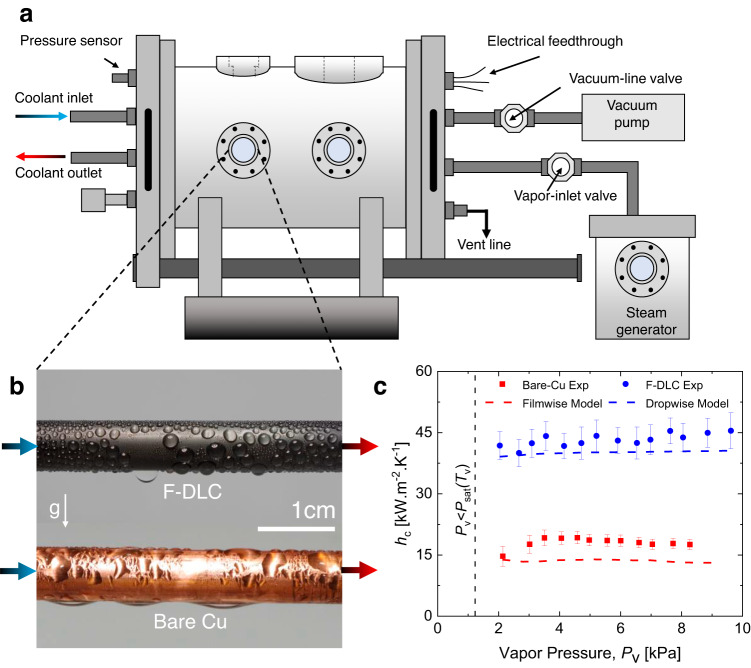
Enhanced condensation heat transfer of F-DLC. **a** Schematic of the chamber (not to scale) used for the condensation heat transfer experiments. Full details of the chamber and its operation can be found in Supplementary Notes 4a and 4b^54,55^. The Cu tube sample having outer diameter *D*_OD_ = 9.53 mm, inner diameter *D*_ID_ = 8.0 mm, and length *L* = 134.6 mm was cooled via chilled water flowing inside of the tube at 8 ± 0.2 L min^−1^. **b** Optical images during condensation showing (top) dropwise condensation on the F-DLC coated smooth Cu tube, and (bottom) filmwise condensation on the smooth uncoated hydrophilic Cu tube. The chamber vapor pressure *P*_v_ = 2.67 ± 0.15 kPa was identical for both tests. **c** Experimentally measured and theoretically computed steady-state condensation heat transfer coefficient (*h*_c_) as a function of saturated steam vapor pressure (*P*_v_) on the F-DLC coated Cu (dropwise) and bare Cu (filmwise) tubes. Error bars were computed using the propagation of error (see Supplementary Note 4c). The theoretical prediction for dropwise condensation (blue dotted line) was obtained using the classical droplet growth and distribution model (see Supplementary Note 4d). The theoretical prediction for filmwise condensation (red dotted line) was obtained using the Nusselt filmwise condensation model for a single horizontally oriented tube (see Supplementary Note 4e). For details about the experimental facility, test protocols, and uncertainty propagation, refer to Supplementary Notes 4a, 4b, and 4c.

Due to the demonstrated F-DLC conformity, low surface energy, and good thermal conductivity compared to polymeric hydrophobic coatings, the F-DLC coating has high potential to enhance condensation heat transfer performance. To determine the overall condensation heat transfer performance, an F-DLC coated copper (Cu) tube was fabricated following the same process described for the flat coupons and condensation heat transfer experiments were performed in a chamber with a controlled environment (Fig. 3a and Supplementary Fig. 3). Condensation heat transfer behavior and condensate shedding were benchmarked by comparison to an uncoated Cu tube sample having identical diameter and length. Prior to performing the experiments, the water vapor supply was boiled, and the test chamber was evacuated to a pressure $P$ < 1 Pa to eliminate noncondensable gases and diffusional mass transfer resistances, key to obtaining high fidelity measurements^21–24^. Throughout the experiments, the chamber pressure and temperature were continuously monitored to ensure saturated conditions. The temperature of the sample tubes was independently controlled via a cooling loop, and the inlet and outlet tube temperatures were measured using high accuracy resistance temperature detectors to determine the coolant enthalpy change and relate it to the measured condensation heat flux. For all experiments, the cooling water inlet temperature was kept constant at 7 ± 1 °C with a coolant flow rate of 8.0 ± 0.2 L min^−1^, resulting in fully turbulent internal flow with Reynolds number, ${{{{{{\rm{Re}}}}}}}_{{{{{{\rm{D}}}}}}}$ = 24,000. Condensation heat transfer performance was tested within the vapor pressure range of 3.5 <${P}_{{{{{{\rm{v}}}}}}}$ < 10.0 kPa, which are common conditions used for condensers in steam-cycle power generation applications^25,26^. For full details regarding the experimental facility, test protocols, and full uncertainty propagation analysis, refer to Supplementary Notes 4a, 4b, and 4c.

Figure 3b shows optical images obtained during dropwise condensation on the smooth F-DLC-coated hydrophobic Cu tube (top image, Supplementary Movie 1) and during filmwise condensation on the clean, uncoated hydrophilic Cu tube (bottom image). As expected, on the bare Cu tube, vapor condensed and formed a thin liquid film that covered the entire surface. On the F-DLC coated Cu tube, stable dropwise condensation ensued. To maximize the tube internal heat transfer coefficient, the cooling water mass flow rate was held constant at 8.0 ± 0.2 L min^−1^ for all experiments (1.0 < $S$ ≤ 1.7, 8.5 °C < ${T}_{{{{{{\rm{s}}}}}}}$ < 25.0 °C, where $S$ is the supersaturation and ${T}_{{{{{{\rm{s}}}}}}}$ is the extrapolated tube surface temperature). The condensation heat transfer coefficient (${h}_{{{{{{\rm{c}}}}}}}$) was calculated from the measured condensation heat flux and overall heat transfer coefficient. By calculating the well-validated thermal resistances of the internal tube single-phase forced convection, radial conduction through the Cu tube wall, and F-DLC coating thermal resistance using the measured thermal conductivity, the steady-state condensation heat transfer coefficient at the tube outer surface, ${h}_{{{{{{\rm{c}}}}}}}$, was calculated. See Supplementary Note 4c for detailed calculation methodology and full uncertainty analysis. Figure 3c shows calculated and predicted heat transfer coefficient as a function of vapor pressure. The F-DLC coated Cu sample showed a ~3× higher condensation heat transfer compared to the uncoated Cu tube over a wide range of vapor pressures. To compare the experimental results to theoretical predictions, we calculated the dropwise condensation heat transfer coefficient (blue dotted line in Fig. 3c) using the droplet growth and distribution model (see Supplementary Note 4d) and the filmwise condensation heat transfer coefficient (red dotted line in Fig. 3c) using the Nusselt condensation model on a horizontal tube (see Supplementary Note 4e). The experimentally measured dropwise and filmwise heat transfer coefficient is in good agreement with the well-validated dropwise and filmwise condensation models, respectively.

### Long-term steam condensation durability

**Fig. 4:**
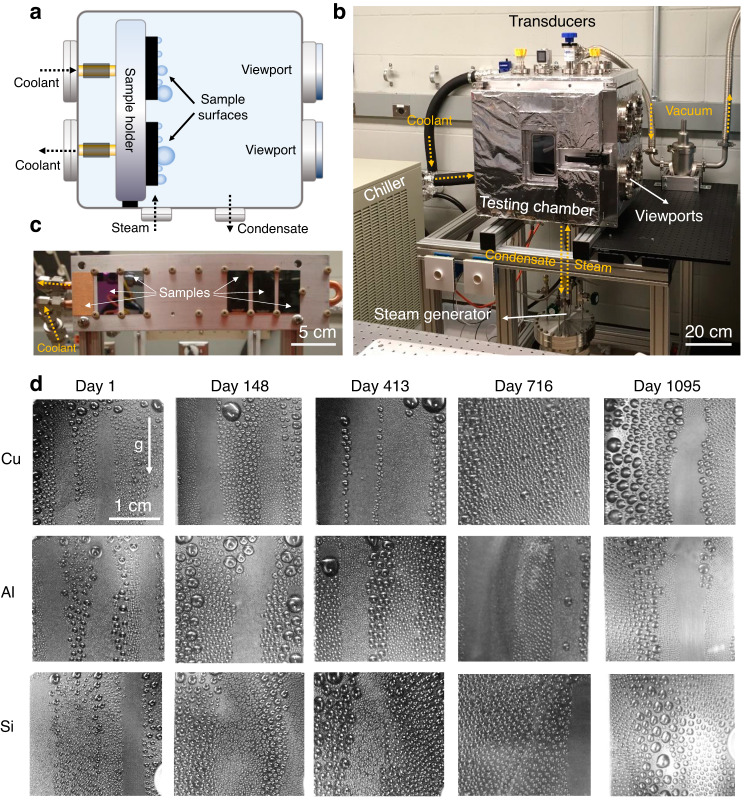
Long-term steam dropwise condensation on F-DLC. **a** Schematic and **b** photograph of the facility used to conduct long-term condensation experiments. **c** Image of the cold plate and sample assembly placed inside of the vacuum chamber for experiments. Throughout the experiment, the surface temperature of the samples was maintained by supplying coolant at 10 °C to the cold plate from a dedicated chiller. Steam is generated inside a separate degassing chamber connected to the bottom of the chamber. **d** Time-lapse images of steam dropwise condensation on the vertically oriented surfaces consisting of F-DLC coated: (top row) polished Cu, (middle row) polished Al, and (bottom row) polished Si wafer. All results showed hydrophobicity of the F-DLC coating irrespective of substrate for a period lasting more than 3 years at the time of writing this manuscript (March 2022).

The F-DLC coating provides a conformal, pinhole free, adherent solution for the rational design of multiple layers that can enable enhanced condensation heat transfer (Fig. 3). The demonstrated condensation heat transfer results are not unique, showing similar performance with previously developed hydrophobic materials^5,21,27,28^. However, the potential durability of F-DLC when compared to classical hydrophobic coatings makes it beneficial. To evaluate the long-term durability of the F-DLC coating during steam condensation, we built a separate vacuum-compatible condensation chamber (Fig. 4a, b). To accelerate the condensation process and increase the condensation heat flux, the testing samples were mounted on an aluminum cold plate (Fig. 4c) and placed in the custom-built environmental chamber commissioned in 2017. The environmental chamber was first evacuated to $P$ < 5 Pa to remove noncondensable gases, then, hot steam was injected into the chamber from a boiler after following a detailed degassing procedure. Cold water at ~10 °C was supplied to the cold plate inlet via a chiller, which reduces the surface temperature of the mounted samples. As the steam comes in contact with the F-DLC surfaces it forms water droplets and initiates dropwise condensation. Due to the good droplet mobility on the hydrophobic F-DLC surfaces, the condensed water droplets shed from the surface toward the condensate collector at the bottom of the chamber and finally the condensate returns to the boiler by gravity. During the long-term test, the chamber pressure ranged from 2 to 3 kPa. For details of the chamber, control systems, and operational procedure, see Supplementary Note 5.

A total of three F-DLC coated substrates consisting of a polished silicon wafer, a Cu tab, and an Al tab were tested in the chamber system for the full-time duration. The condensation conditions of each sample were recorded at least twice a week via visual observation, which has been used in the past to identify the condensation mode (dropwise or filmwise condensation). Due to inherent leaks into the chamber from the pressure difference arising from the atmosphere and the saturated steam at lower pressure, the system had to be shutdown periodically (once every month) in order to vacuum out the chamber and ensure pure saturated conditions. During shutdown, the chamber was not opened and simply was vacuumed to remove non condensables, with shutdown lasting no more than 3 h.

Figure 4d shows time-lapse images of condensation on each sample mounted in the vertical orientation. The images show that condensing droplets on the F-DLC surfaces have clear circular shapes and grew to 3.2–3.5 mm in diameter prior to departing the surface throughout the testing period, which lasted for 3 years (>1095 days). Even after the long-term exposure to saturated steam conditions, the F-DLC coating exhibited continued dropwise condensation during tests, regardless of the base substrate. This was not true for control samples consisting of Cu and Al tabs coated with self-assembled monolayers of (heptadecafluoro-1,1,2,2 tetrahydrodecyl)trimethoxysilane (HTMS), which failed via transition to filmwise condensation after less than one month of testing (see Supplementary Table 4). After exposing to steam for 3 years, the DI water contact angle on the F-DLC coating changed from ${\theta }_{{{{{{\rm{a}}}}}}}/{\theta }_{{{{{{\rm{r}}}}}}}$ = 99.1°/86.2° to ${\theta }_{{{{{{\rm{a}}}}}}}/{\theta }_{{{{{{\rm{r}}}}}}}$ = 70.5°/57.1° (see Supplementary Table 5). As a comparison, we also conducted durability assessment on iCVD coatings (DVB-PFDA films) having different film thicknesses (30 nm and 60 nm) in the same environmental vacuum chamber system (see Supplementary Fig. 7). Both iCVD surfaces showed unstable dropwise condensation (irregular shaped droplets) after 1 week of steam exposure, in agreement with recent work^29^. See the “Methods” section for details of the HTMS and iCVD hydrophobic surface fabrication.

We conducted XPS analysis on the tested samples to investigate the surface chemistry change after 3 years of steam condensation. The results show that the content of fluorine in the top f-DLC layer reduced from 9.5% to 4.9% during the 3-year test (see Supplementary Table 5), which indicates that the surface energy increases during prolonged exposure to steam and condensation, thus reducing the apparent contact angle. We also performed condensation experiments and analyzed the condensate droplet departure size variation on both fresh and 3-year condensation tested F-DLC surfaces (see Supplementary Fig. 8). The 3-year tested samples showed a 25% increase in the droplet departure size when compared to the fresh F-DLC coating. However, the tested F-DLC surfaces still maintain a low droplet Bond number (${{{{{\rm{Bo}}}}}}={R}_{{{{{{\rm{f}}}}}}}^{2}/{l}_{{{{{{\rm{y}}}}}}}^{2}$, where ${R}_{{{{{{\rm{f}}}}}}}$ is the characteristic lateral length of the liquid droplet, taken to be its final equilibrium radius immediately before departure, and ${l}_{{{{{{\rm{y}}}}}}}$ is the capillary length) representing the ratio of equilibrium droplet radius immediately before departure to capillary length scale of water. The ${{{{{\rm{Bo}}}}}}$ number mediated dropwise-to-filmwise transition occurs at ${{{{{{\rm{Bo}}}}}}}_{{{{{{\rm{crit}}}}}}}=1.4$, with ${{{{{\rm{Bo}}}}}} > 1.4$ predicting gravitationally dominated puddle formation and filmwise condensation^20^. Calculations of ${{{{{\rm{Bo}}}}}}$ for each 3-year tested F-DLC surface, based on the final surface contact angle measurement, reveal a maximum ${{{{{\rm{Bo}}}}}}$~0.5 (Supplementary Table 6). Since ${{{{{\rm{Bo}}}}}} < {{{{{{\rm{Bo}}}}}}}_{{{{{{\rm{crit}}}}}}}$, capillary-dominated hemispherical droplet shapes are maintained at departure, ensuring dropwise condensation and high droplet mobility even after 3 years of condensation testing. See Supplementary Note 5c for details regarding the Bond number model.

Furthermore, TDTR measurements of the 3-year tested F-DLC samples show insignificant change (<1%) in the thermal conductivity (see Supplementary Table 5). Even after 3 years of condensation, the F-DLC coatings demonstrate the capacity to maintain comparable droplet mobility and thermal conductivity. As a result, the heat transfer performance remains similar when compared to fresh F-DLC surfaces (see Supplementary Fig. 9).

### Thermomechanical robustness

Abrasion resistance and stability at elevated temperatures is required for a broader array of hydrophobic coating applications. The thermal stability of the F-DLC coating was tested by heating samples in a furnace (Lindberg 2″ Tube Furnace) with a flow of inert gas for various times and temperatures. A continuous flow of N_2_ gas (~ 3 LPM) in the tube furnace was used. After heating for a prescribed time and allowing the sample to cool back down to room temperature, the apparent advancing (${\theta }_{{{{{{\rm{a}}}}}}}$) and receding (${\theta }_{{{{{{\rm{r}}}}}}}$) contact angles of DI water droplets were measured using a microgoniometer (MCA−3, Kyowa Interface Science). The heating temperature ranged from 100 °C to 600 °C and at each temperature, five identical F-DLC coated samples were placed in the furnace. After 1 h, the first sample was taken out, followed by the rest of the samples taken out sequentially at 2, 4, 8, and 16 h. The change in apparent contact angle represents an indication of coating integrity due to the intrinsic hydrophilicity of the substrate (metal or native oxide) beneath the F-DLC coating.

**Fig. 5:**
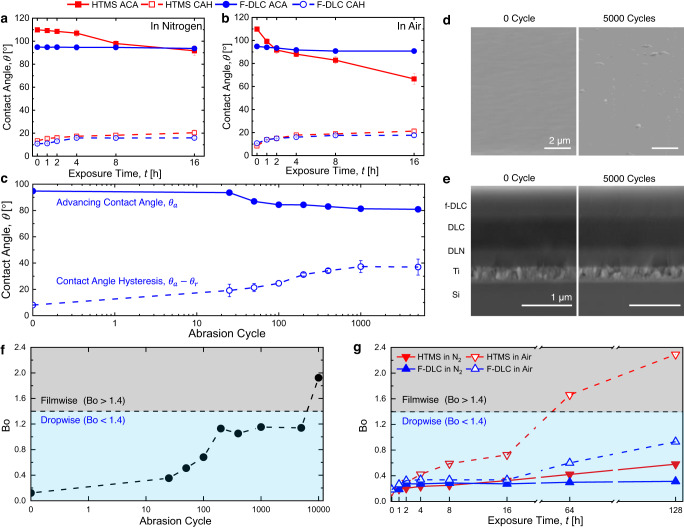
Thermomechanical robustness of F-DLC. Thermal stability of the F-DLC coating compared with HTMS SAM at 300 °C in **a** nitrogen (N_2_) and **b** air environments as measured by the change in apparent advancing contact angles (ACA) and receding contact angles (RCA) with DI water droplets. Contact angle hysteresis (CAH) represents the arithmetic difference between ACA and RCA. Legends are same for (**a**) and (**b**). To characterize the effects of abrasion, **c** the DI water droplet apparent advancing contact angle (blue solid curve) as well as the contact angle hysteresis (blue dashed curve) is plotted as a function of abrasion cycle. The apparent advancing contact angle on F-DLC reduced to ~81° after 5000 abrasion cycles due to **d** increased surface roughness and **e** reduced thickness of the top f-DLC layer after abrasion. After 5000 abrasion cycles, the thickness of the F-DLC coating reduces from ~1.65 µm to ~1.61 µm, resulting in exposure to lower fluorine content regions and reduced hydrophobicity. **f** Dropwise condensation modelling reveals that the droplet Bond number (Bo) is ~1.14 after 5000 abrasion cycles, less than the critical Bond number (Bo_crit_ = 1.4) for dropwise-to filmwise transition. **g** During high temperature exposure in 300 °C conditions (air), the HTMS surface transitions to filmwise condensation, while the F-DLC coating maintains dropwise condensation. For (**a**–**c**), the error bars indicate the standard deviation, which was determined based on three independent measurements conducted for each data point.

To characterize the effects of potential oxidation in non-inert (atmospheric pressure) conditions, identical experiments were also conducted in an atmospheric pressure oven (Lindberg/Blue M Moldatherm Box Furnace). The thermal stability of the F-DLC coating was compared to a control sample consisting of a HTMS SAM hydrophobic coating deposited on identical substrates. As shown in Fig. 5a, b, the F-DLC coating maintains consistent hydrophobicity up to 300 °C exposure temperatures both in N_2_ and air environments, with air exposure creating slight degradation in hydrophobicity due to substrate oxidation. However, on the HTMS coated control samples, 300 °C exposure resulted in complete degradation (hydrophilicity) after 2 h of heating due to silane desorption. In the inert N_2_ environment, the F-DLC coating maintained continual hydrophobicity up to 450 °C (Supplementary Fig. 10a, b). In air at 350 °C (Supplementary Fig. 10c) and 400 °C (Supplementary Fig. 10d), F-DLC degrades at a similar rate for 2 h of heating time. Then, at later times (>2 h), the oxidation rate was observed to significantly accelerate for the 400 °C case. For transient applications, where the temperature cycles through high and low setpoints, our F-DLC coating showed excellent durability and low thermomechanical stress (Supplementary Fig. 11).

To evaluate long-term mechanical durability of the F-DLC film against abrasion and wear, cyclic abrasion resistance tests were performed on a Taber abrasion tester (Supplementary Fig. 12). A silicon wafer coated with F-DLC was placed underneath an abrasive tip with a defined preload of 1 N on the tested surface. Then, a fixed speed linear reciprocating motion was applied to the sample stage to model abrasion, followed by DI water contact angle measurements on the abraded surface area at a prescribed number of abrasion cycles. Prior to contact angle measurement, residuals consisting of mainly abrasive nanoparticles and binder material on the test surface from the abrasion tips were cleaned by sonication of samples in ethanol. This ensured that the measured change in contact angle was due to damage during abrasion, and not parasitic material deposition. Details of the thermal stability and abrasion tests are included in Supplementary Note 8.

Figure 5c demonstrates that after 5000 abrasion cycles, ${\theta }_{{{{{{\rm{a}}}}}}}$ on the tested F-DLC surface decreased gradually from ~97.5° (fresh F-DLC surface) to ~81.0°. The contact angle hysteresis (Δ*θ* = *θ*_a_ − *θ*_r_) of the tested surface increased from ~8.0° to ~37.0°. The wettability changes due to the combined effects of increment of local surface energy from partial removal of the top fluorinated layer (f-DLC), and formation of local microscale surface roughness (Fig. 5d). The EDS line scan (Supplementary Fig. 13) of the F-DLC cross section shows the concentration of fluorine in the top f-DLC layer decreases from top to bottom, resulting in a surface energy gradient along the thickness of the f-DLC layer. Cross-sectional SEM analysis shows that after 5000 abrasion cycles, the total coating thickness reduces from ~1.65 µm to ~1.61 µm, only a removal of ~40 nm of the top f-DLC layer, exposing the surface to the lower fluorine content depth, and reducing hydrophobicity. Here we observe that <10% the material was removed after 5000 abrasion cycles, mainly due to the wear resistance originating from the coefficient of friction (COF) DLC material^30^. The observed abrasion resistance of our F-DLC coating is even better than H-terminated DLC because the two fluorine terminated DLC surfaces sliding against each other would exert higher repulsive forces compared to two H-terminated surfaces. Thus, the mutual interaction of two F-DLC surfaces results in a lower COF^31^.

Although the F-DLC surface can be characterized as hydrophilic (*θ*_a_ < 90°) after 50 abrasion cycles, dropwise condensation and exceptional droplet mobility is maintained due to the maintenance of low contact angle hysteresis^20^. In fact, the F-DLC surface is predicted to maintain dropwise condensation even after 5000 abrasion cycles due to the maintenance of low droplet Bond number (${{{{{\rm{Bo}}}}}} \, <$ 1.4). Calculations of ${{{{{\rm{Bo}}}}}}$ for each tested surface based on the abraded surface contact angle measurements reveal a maximum ${{{{{\rm{Bo}}}}}}$ ~ 1.14 after 5000 abrasion cycles. Since ${{{{{\rm{Bo}}}}}} < {{{{{{\rm{Bo}}}}}}}_{{{{{{\rm{crit}}}}}}}$, dropwise condensation occurs on the abraded samples. Continued abrasion experiments up to 10000 cycles showed that our F-DLC coating eventually transitions to droplet pinning and filmwise condensation behavior with Bo ~ 1.9 (Fig. 5f).

Consistent with the previous wettability characterization during heating in air at 300 °C, the HTMS coated surface showed a drastic increase in droplet ${{{{{\rm{Bo}}}}}}$ with little change for the F-DLC coating (Fig. 5f). In the inert environment, the F-DLC coating did not show filmwise transition (${{{{{\rm{Bo}}}}}} < 1.4$) until exposure to 600 °C for ~10 h (Supplementary Fig. 10e). However, the filmwise transition on the F-DLC coating is predicted to occur after exposure to 400 °C in air after ~10 h (Supplementary Fig. 10f). Since the F-DLC coating showed good hydrophobicity in both air and inert environments for up to 16 h of exposure at 300 °C, we extended the heating time up to 128 h to try to observe the filmwise transition. As shown in Fig. 5g, even after 128 h of exposure to hot air (300 °C) the F-DLC coating mainained dropwise condensation (Bo ~ 0.9 < 1.4). See Supplementary Note 5c for details regarding the Bond number model.

## Discussion

Many sustainability-relevant applications requiring hydrophobicity have been limited to laboratory scale experiments due to poor durability. Low surface energy hydrophobic coatings are known to enhance condensation. The majority of hydrophobic coatings developed in the past have been shown to degrade within one month of condensation exposure. In addition to wet conditions and condensation, hydrophobic coating resiliency to mechanical deformation or abrasion during application, handling, manufacturing, and operation is a critical bottleneck to coating implementation in real and societally impactful systems.

A summary of past attempts to achieve durable hydrophobicity shows limited progress to simultaneously achieve low surface energy, high thermomechanical robustness, and durability in pure-steam environments (see Table 1 and Supplementary Table 7). Many studies do not conduct detailed surface characterization after condensation durability tests. While some have reported year-long resiliency to condensation, many of these experiments were not conducted in pure-steam conditions. Instead, atmospheric condensation in the presence of noncondensable gases (NCGs) were used. Condensation in the presence of NCGs is typified by a low condensation rate, and a gentler environment on the coating under test. To clearly demonstrate the effect of accelerated condensation on coating lifespan, we performed a durability study at two different pure-steam vapor pressures devoid of NCGs. The hydrophobic surface exposed to a higher vapor pressure (~25 kPa) failed within 40 min of condensation initiation due to the high condensation rate and harsher environment (see Supplementary Fig. 14). However, the same hydrophobic surface lasted approximately 30 days when exposed to a lower vapor pressure (~2 kPa) and slower condensation rate. Hence, reported coating lifespans for experiments conducted in pure-steam conditions are characteristic of higher condensation rates, and are thus better representations of coating durability. This is stark contrast to condensation longevity studies conducted in the presence of NCGs, which have lower condensation rates, and gentler conditions. When compared to previous approaches, F-DLC represents a synergistic combination of parameters required to achieve robust hydrophobicity (see Table 1 and Supplementary Table 7). The architecture of our F-DLC coating is specifically optimized for the required robustness functionalities such as: strong interfacial toughness, thermal buffering, mechanical robustness, low surface energy, and pinhole prevention. Our 3-year pure-steam condensation durability is the longest reported in literature to date. Furthermore, F-DLC presents a pathway to improve the longevity of low energy coatings in manufacturing, biomedical, and marine applications, owing to its tribological and mechanical properties as well as demonstrated corrosion resistance, biocompatibility, and hemocompatibility^32–37^.

**Table 1:** Performance comparison of state-of-the-art hydrophobic coatings from literature

Coating	${{{\boldsymbol{\theta}}}}_{{{\rm{a}}}}$	*t* [µm]	*G* [J/m^2^]	*E* [GPa]	Abrasion resistance [# cycles]	Heat transfer coefficient [kW/m^2^K]	Thermal stability [°C]	Condensation durability [h]	Refs.
Vitrimer	93 ± 3°	~0.01	1	0.1	1	N/A	<200	408	^48^
Armor-polymer	>170°	>10	N/A	N/A	1000	N/A	<200	N/A	^11^
PKFE composite	~160°	>50	N/A	N/A	100	N/A	N/A	N/A	^10^
Candle soot	>160°	>30	N/A	N/A	1	N/A	<400	N/A	^12,49^
Polymer infused porous surfaces	~105°	~20	N/A	N/A	N/A	~ 130	<250	4800	^50^
Lipid-double layer	163° ± 2°	~0.1	0.8	4	1	N/A	<200	8760	^51^
Cu with SAM	~110°	<0.002	N/A	N/A	1	~15	<200	6480	^52^
SLIPS	~120 ± 3°	~2	N/A	N/A	N/A	N/A	<150	1080	^53^
F-DLC	**~97** ± **1°**	**~1.65**	**100**	**~80**	**>5000**	**~45**	**>300**	**26,280**	**This work**

Detailed comparison of properties from major published works are included in Supplementary Table 7. Cells labeled with N/A indicate that data were not measured. Symbols ${\theta }_{{{{{{\rm{a}}}}}}}$, *t, G, E*, refer to apparent advancing deionized water droplet contact angle, coating thickness, coating-substrate adhesion, and Young’s modulus, respectively. Coatings lifespan studies conducted during condensation in the presence of NCGs are not included in the table (see Supplementary Table 7).

Bold font is used to highlight the current work.

Achieving stable dropwise condensation on hydrophobic surfaces has a profound impact on global energy production, water conservation, and hence the carbon footprint and sustainability of industrialized and developing nations. The Electric Power Research Institute (EPRI) reports that a 1% heat rate increase of a 500 MW_e_ power plant operating at 80% capacity saves approximately $700,000 in annual costs and reduces CO_2_ emissions by 40,000 tons per year^38^. Seventy percent of global electricity is produced by steam power plants (natural gas, coal, nuclear, biofuel), which use an estimated 100 trillion gallons of water each year for cooling^39^. Power plant efficiency is sensitive to condenser performance. Dropwise condensation enhances the vapor-side heat transfer coefficient of the condenser and increases the difference between the evaporation and condensation temperatures across the power cycle. Thus, the overall system can operate at a higher efficiency or a decrease in overall size, weight and ultimately capital cost^40^. When designing the hydrophobic condenser, greater focus is needed to enhance the hydrophobic coating durability rather than maximizing the coating hydrophobicity (or superhydrophobicity). Overall power plant efficiency gains stagnate after a certain threshold of condensation heat transfer coefficient is achieved^3,40^. As a clear and economically viable use case, integration of our F-DLC surfaces within condensers of steam-cycle power plants represents an opportunity for 2% efficiency enhancement, societal greenhouse gas emissions reduction, as well as a 1.9% reduced levelized cost of electricity (LCOE)^3^.

Although our work demonstrates high durability for small-scale F-DLC surfaces (~0.1 m), the overall life cycle cost (LCC) $/m^2^/yr remains reasonable. When compared to a conventional SAM hydrophobic coating (~$800/m^2^/yr), the LCC of F-DLC (~$1400/m^2^/yr) is ~72% higher but shows remarkable durability and offers a promising payback period (see Supplementary Note 11 for detailed economic assessment). We are currently collaborating with Abbott Power Plant at the University of Illinois to develop a larger-scale (tube length of ~0.6 m) condenser prototype that will undergo testing in the power plant environment with higher steam pressure (>100 kPa) and temperature (>100 °C). We acknowledge that this prototype is still not on an industrial scale, but our F-DLC deposition process, which combines PVD and PACVD in the same vacuum system, presents scalability and cost reduction opportunities. Due to the large global physical vapor deposition (PVD) market size, PVD manufacturing can significantly reduce coating costs when scaled up^41^. Additionally, in many applications, compact heat exchanger design is crucial, particularly in nuclear power plants, submarines, and navy ships. For these applications, the efficiency, reduced weight-volume and extended durability are of utmost importance, as opposed to cost^42,43^. Thus, F-DLC-coated compact and durable heat exchangers are promising.

In summary, our developed multilayer F-DLC coating has low surface energy characteristic of non-polar polymers, with a high Young’s modulus approaching that of metals. We demonstrate the versatility of F-DLC on a wide range of substrates including crystalline, non-crystalline and common engineering metals, all showing similar surface energy after coating. The F-DLC not only demonstrates enhanced dropwise condensation heat transfer, but also durability in moist environments for a period of more than 3 years. Characterization of the compatibility of F-DLC in elevated temperature environments exceeding 300 °C and sustainability after 5000 mechanical abrasion cycles demonstrates resiliency. The outcomes of our work not only develop a low surface energy coating capable of implementation for a plethora of versatile applications, it overcomes the challenge of generating hydrophobic surfaces that can achieve extended lifetime during exposure to harsh thermomechanical environments. Furthermore, F-DLC coatings have the potential to enhance the sustainability of non-renewable energy generation sources, thus helping societies reach their climate change goals through carbon emissions reduction and lower utilization of environmentally harmful fossil fuels.

## Methods

### F-DLC nanofabrication method

The four layer F-DLC coating manufacturing process uses a combination of PVD sputtering and PACVD. The Ti layer is sputtered on the substrate and while the remaining three layers are deposited with PACVD in a vacuum chamber at 250 °C. On the Ti-coated substrate, DLN is deposited by PACVD. Hydro-carbonated DLN gases integrated with silicon and oxygen provide the deposition of a soft DLN (a-C:H:Si:O) layer. Next the DLC layer is deposited in the same environment. Finally, the fluorine precursor in liquid form is introduced to the system which is then vaporized and co-deposited with the final DLC layer at the end of the deposition process yielding a top fluorinated DLC (f-DLC) layer.

### Cross-sectional SEM

The cross-section imaging of a multilayer F-DLC coating on a polished silicon substrate was prepared through focused ion beam (FIB, Thermo Scios2 Dual-Beam SEM/FIB) milling. The ion beam voltage and current were set into 30 kV and 15 nA, respectively. After milling, the coating cross-section was imaged by ultra-high resolution SEM.

### Energy-dispersive X-ray spectroscopy (EDS) analysis

Qualitative chemical element analysis for each layer of the F-DLC coating was conducted with EDS (JEOL JSM 7000 F Analytical SEM/EDS). The accelerating voltage was set to 10 kV during the analysis.

### X-ray photoelectron spectroscopy (XPS)

XPS was performed using a monochromatic Al Kα-source (Kratos Axis Ultra, Kratos Analytical). The size of the source beam was 2 mm × 2 mm, and the size of the analyzed region was 0.3 mm × 0.7 mm. The instrument was maintained at a pressure of 10^−7^ Pa during the experiments. The spectra were post processed with CasaXPS software to determine the change in composition of the sample surfaces.

### Time-domain thermoreflectance (TDTR)

Prior to thermal conductivity measurement, the sample was coated with a 111 ± 2 nm-thick Al film by magnetron sputtering (AJA Orion3 Sputter Coater). It was assumed that the film was free of adsorbed water because the sputtering procedure involved pumping the sputter chamber down to $\approx$1.33 × 10^−5^ Pa. The TDTR system uses a mode-locked Ti:sapphire laser tuned to 785 nm and a repetition rate of 74.6 MHz^9^. The output laser was split into separate pump and probe beams by a polarizing beam splitter. The power of the pump beam was set to 10 mW, and the probe beam power was set to 5 mW. The intensity of the pump beam was modulated with an electro-optic modulator at $f=$10.1 MHz synchronized with an RF lock-in amplifier. The time delay of the arrival of the pump and probe beam to the surface was adjusted by a mechanical delay stage in the path of the probe beam. The pump and probe beams were overlapped on the sample and the beams were focused using a 5× objective lens. The laser spot size was ≈ 10.7 µm. A photodiode was used to detect the reflected probe beam and the light from the pump beam was blocked from reaching the detector by spatial filtering and two-tint wavelength filtering. The signal was measured by a lock-in amplifier connected to the photodiode. The thickness of F-DLC ($1.65\pm 0.05$ µm) is measured by cross-section SEM imaging. $\sqrt{kC}=\left(1.07\pm 0.05\right)\times {10}^{-3}$(W s^1/2^)/(m K). The heat capacity of amorphous carbon film is usually within the rage of 2.5 J/(cm^3^ K)^44^, hence the thermal conductivity of the film $k=0.46\pm 0.05$ W/(m K). The error comes from experimental uncertainty (~10%).

### Surface energy measurement

The surface energy was measured by the contact angle method^19^ using Fowkes theory and the Young-Dupre equation^45^. The dispersive, ${E}^{{{{{{\rm{d}}}}}}}$ and polar, ${E}^{{{{{{\rm{p}}}}}}}$ components of the free surface energy were determined by measuring the intrinsic advancing contact angle between the surface and two test liquids with known surface energy components. A non-polar test liquid, diiodomethane was first used to obtain the dispersive component, ${E}^{{{{{{\rm{d}}}}}}}$, where the polar component, ${E}^{{{{{{\rm{p}}}}}}}$ is negligible. Then, deionized (DI) water was used to obtain the polar component, ${E}^{{{{{{\rm{p}}}}}}}$ with known dispersive component, ${E}^{{{{{{\rm{d}}}}}}}$ from the previous measurement. All apparent contact angles were measured using a microgoniometer (MCA-3, Kyowa Interface Science).

### Atmospheric water vapor condensation experiment

To induce water droplet condensation in the atmospheric laboratory environment having air temperature ${T}_{{{{{{\rm{a}}}}}}}$ = 25 ± 1 °C and a relative humidity *RH* = 50 ± 5% (RO120, Roscid Technologies), we placed the samples horizontally on a cold stage (Linkam T95-PE) and reduced the stage temperature to ${T}_{{{{{{\rm{c}}}}}}}$ = 2.0 ± 0.1 °C to enable observation of water vapor condensation on the substrates using a top-view optical microscopy (Nikon Eclipse LV100) coupled to a monochrome camera (Nikon DS-Qi2)^46^.

### HTMS SAM hydrophobic surface fabrication

Polished aluminum and copper tabs were first ultra-sonicated in acetone (10 min), followed by IPA (10 min), then thoroughly rinsed in DI water and dried under a clean N_2_ gas stream. To fabricate the hydrophobic surface, the Al and Cu tabs were functionalized with (heptadecafluoro-1,1,2,2 tetrahydrodecyl)trimethoxysilane (HTMS, TCI America, CAS #: 83048-65−1) using the vapor phase deposition method^47^. Briefly, the substrates were placed in a glass beaker with a vial of HTMS toluene solution (5% v/v). A glass lid was placed on top to seal the container, followed by heating in atmospheric pressure oven (Thermo Scientific, Lindberg Blue M) at 80 ± 5 °C for 3 h to allow conformal HTMS SAM deposition.

### Initiated chemical vapor deposition (iCVD) hydrophobic surface fabrication

Deposition of polymeric PFDA and DVB-PFDA films were performed via iCVD in a stainless-steel vacuum chamber with a base pressure of 5 mTorr, using 1H,1H,2H,2H-Perfluorodecyl acrylate (PFDA, Sigma-Aldrich, CAS#:27905-45-9) as the monomer, Divinyl benzene (DVB, Sigma-Aldrich, CAS#:1321-74-0) as the crosslinker and Tert-Butyl Peroxide (TBPO, Sigma-Aldrich, CAS#:110-05-4) as the initiator. No surface treatments were applied to the surface pre or post deposition. During deposition the chamber pressure was maintained at 300 mTorr using a throttle valve. The substrate and filament temperatures were kept constant at 20 °C and 280 °C, respectively, during the deposition process. The delivery of the precursor vapors into the chamber was controlled by a set of needle valves and the depositions were performed at flowrates of 1.7, 1.6 and 1.2 sccm for PFDA, DVB and TBPO, respectively. The thickness of the samples was monitored real-time using a laser interferometry system mounted on the vacuum chamber and the depositions were stopped when the target thicknesses of 30 nm or 60 nm were reached.

### Supplementary information

Supplementary Information for Publication

Description of Additional Supplementary Files Document

Supplementary Movie

## Data Availability

All data that support the findings of this study are available in the manuscript and in the Supplementary information section. Raw data are available from the corresponding authors upon request.

## Code availability

The code for this study is available from the corresponding author upon request.
