# Supplementary material for: Ultra-resilient multi-layer fluorinated diamond like carbon hydrophobic surfaces
Source: Nat Commun. 2023 Aug 14;14:4902. doi: 10.1038/s41467-023-40229-6 (PMC10425355; doi:10.1038/s41467-023-40229-6)
Supplement: Supplementary file 1 — Supplementary Information for Publication [file 41467_2023_40229_MOESM1_ESM.pdf]

Supplementary Information for

Ultra-resilient multi-layer fluorinated diamond like carbon hydrophobic surfaces

Muhammad Jahidul Hoque[1+], Longnan Li[1,2+], Jingcheng Ma[1+], Hyeongyun Cha[1,3], Soumyadip Sett[1], Xiao Yan[1], Kazi Fazle Rabbi[1], Jin Yao Ho[1], Siavash Khodakarami[1], Jason Suwala[4], Wentao Yang[1], Omid Mohammadmoradi[5], Gozde Ozaydin Ince[5,6], Nenad Miljkovic[1,7,8,9]*

[1]Department of Mechanical Science and Engineering, University of Illinois, Urbana, IL, USA

[2]Current Address: GPL Photonics Laboratory, State Key Laboratory of Luminescence and Applications, Changchun Institute of Optics, Fine Mechanics and Physics, Chinese Academy of Sciences, Changchun, Jilin, 130033 P. R. China

[3]Current Address: Massachusetts Institute of Technology, 77 Massachusetts Avenue, Cambridge, MA, USA

[4]Oerlikon Balzers Coating, Schaumburg, IL, USA

[5]Department of Materials Science and Nanoengineering, Sabanci University, Istanbul, Turkey

[6] Sabanci University Nanotechnology Research and Application Center, Istanbul, Turkey

[7]Materials Research Laboratory, University of Illinois, Urbana, IL, USA

[8]Department of Electrical and Computer Engineering, University of Illinois, Urbana, IL, USA

[9]International Institute for Carbon Neutral Energy Research (WPI-I2CNER), Kyushu University, 744 Motooka, Nishi-ku, Fukuoka 819-0395, Japan

([+]Equal Contribution, *Corresponding Author)

*Corresponding Author Email: nmiljkov@illinois.edu

Supplementary Figures

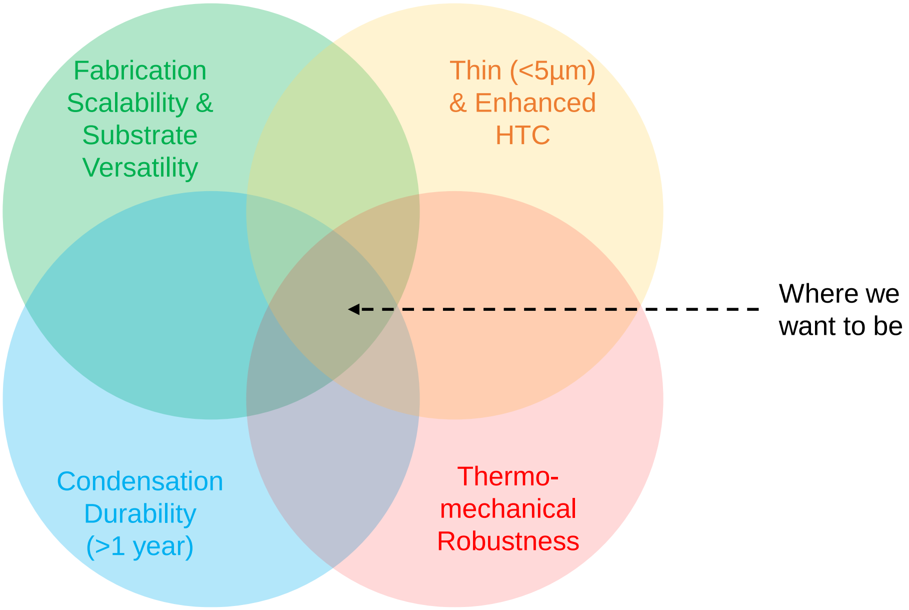

Supplementary Figure 1. Requirements for fabricating a robust hydrophobic coating for energy applications.

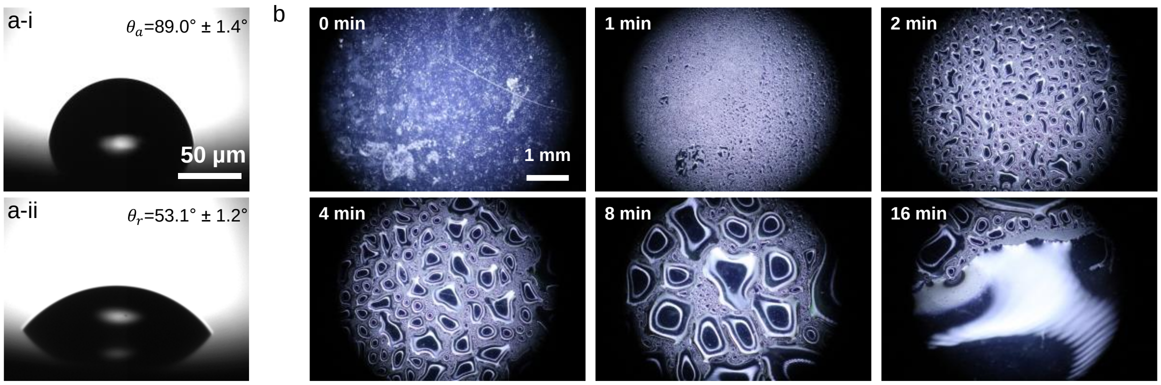

Supplementary Figure 2i. Wettability characterization of traditional DLC surfaces. a(i)-(ii) DI water contact angle showing the contact angle hysteresis (difference between advancing and receding angles) of ~36º leading to (b) non-sustainable dropwise condensation.

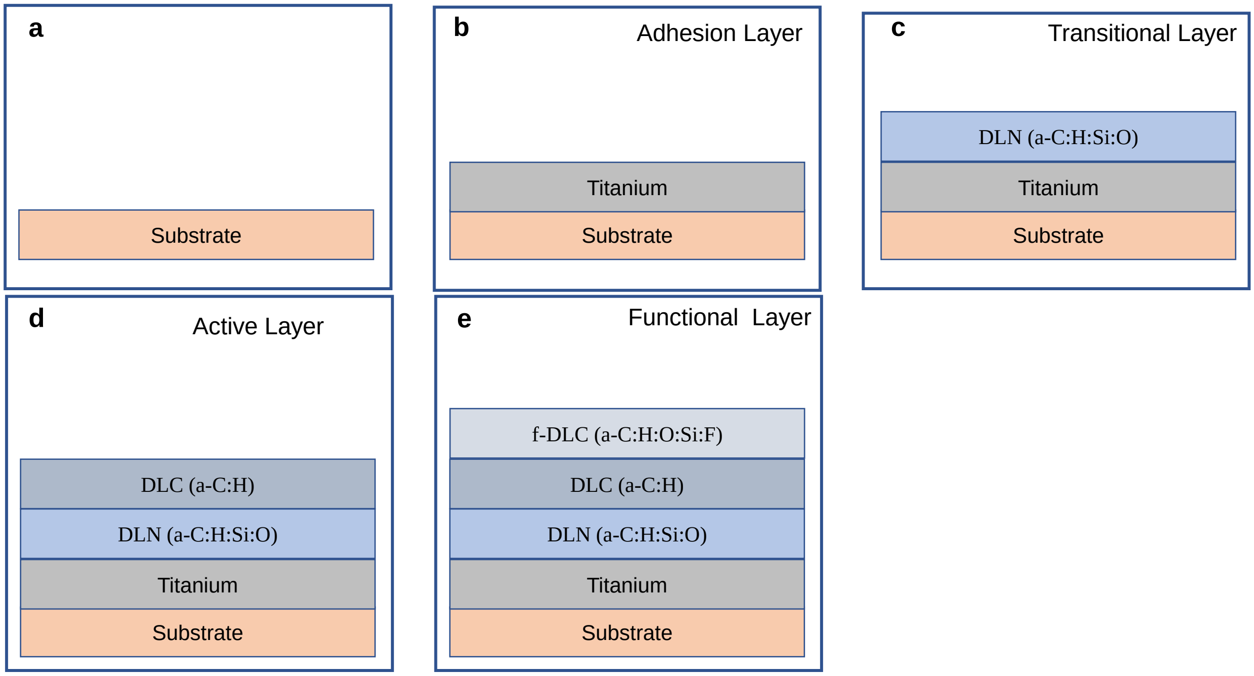

Supplementary Figure 2ii. Fabrication strategy of F-DLC coating showing the deposition of titanium adhesion (sputtered) layer, transitional, active, and functional layer. Transitional to functional layers are deposited using plasma assisted chemical vapor deposition (PACVD).

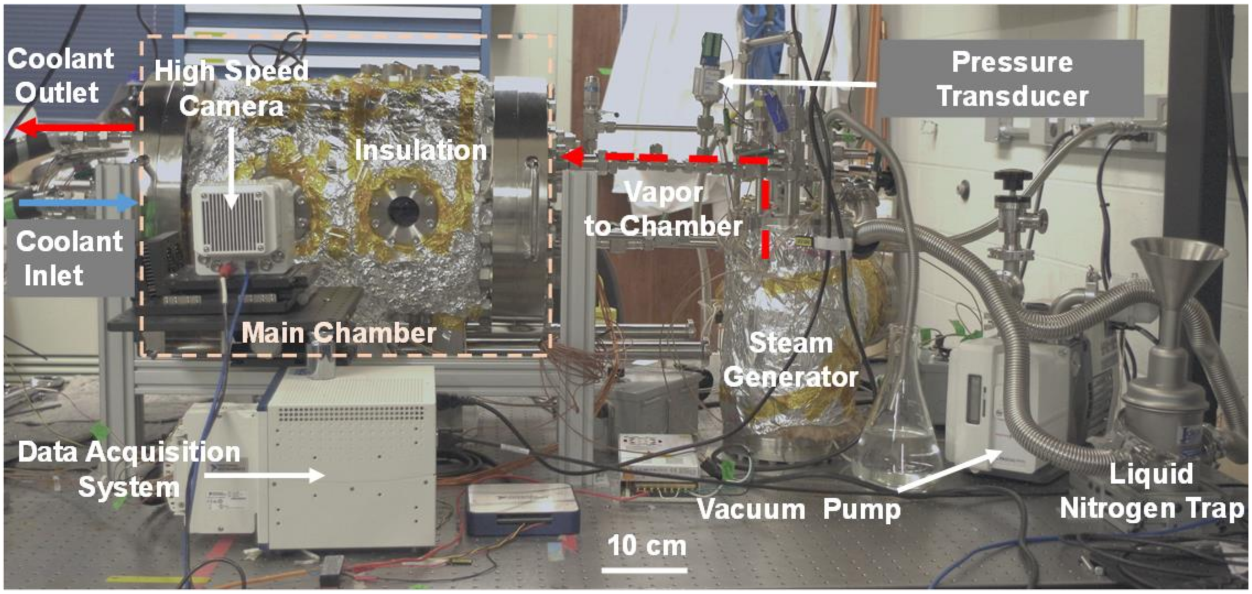

Supplementary Figure 3. Optical Image of the environmental chamber used for condensation heat transfer experiments. This chamber is depicted schematically in Figure 3 of the manuscript.

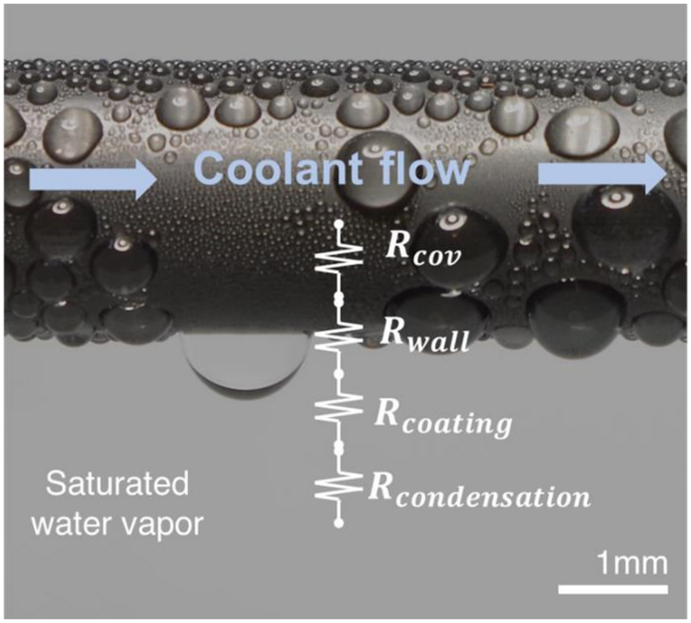

Supplementary Figure 4. Thermal resistance network for condensation of steam on an F-DLC coated tube sample. Resistors not to scale.

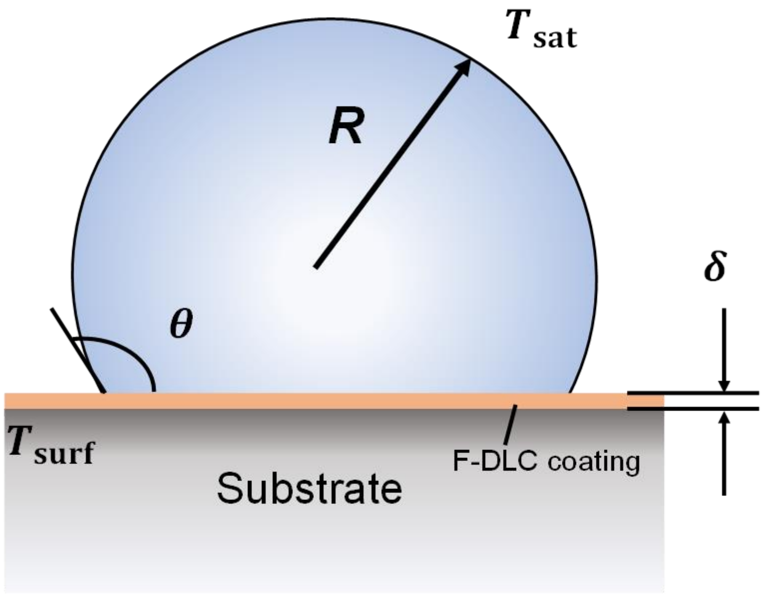

Supplementary Figure 5. Schematic of a condensate water droplet with radius *R* growing on the F-DLC coated (hydrophobic) condensing surface having a total coating thickness of δ.

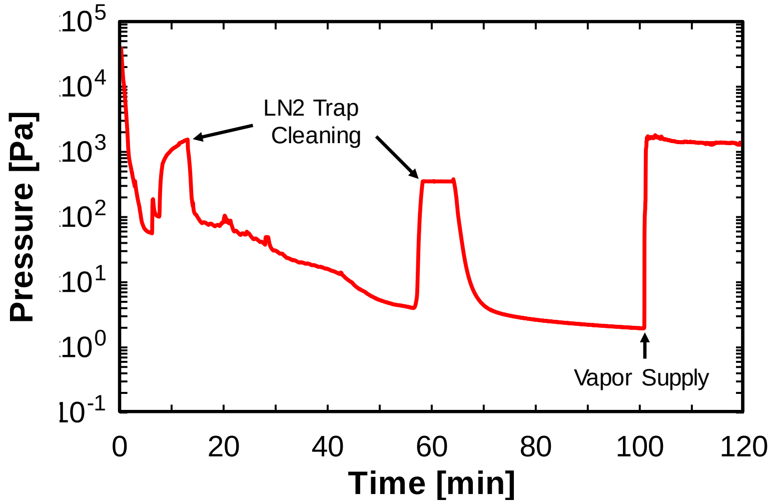

Supplementary Figure 6. Chamber pressure as a function of time during pump-does and degassing operation. The increase in pressure ($t \approx 10$ min and 60 min) is due to cleaning of the liquid nitrogen trap while isolating the chamber. The slight kinks in pressure are due to evaporation of water vapor and leftover ice from a previous experimental run.

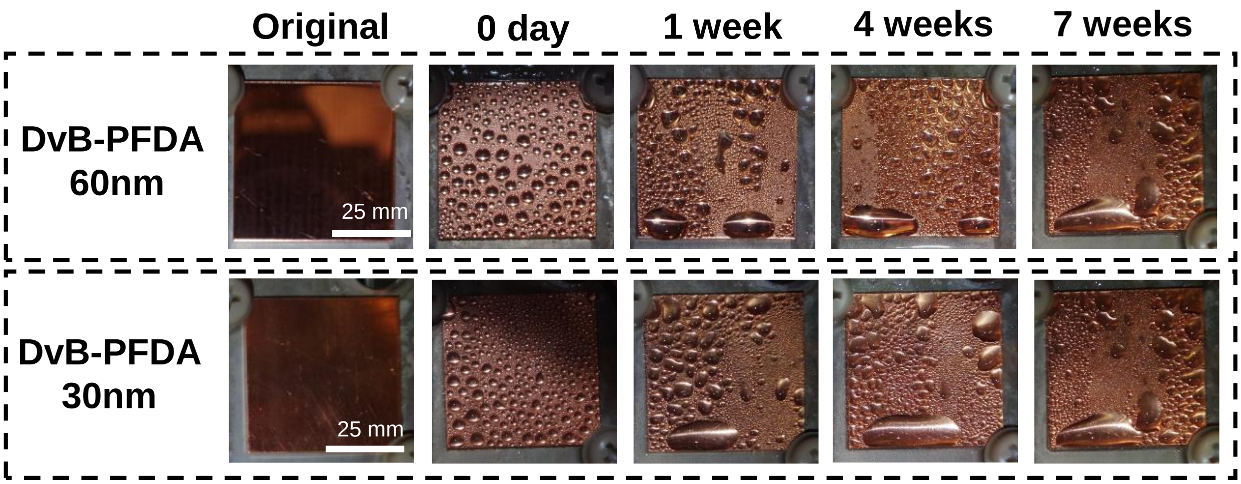

Supplementary Figure 7. Time-lapse images of pure steam dropwise condensation on the vertically oriented surfaces consisting of polished copper coated with (1) 60 nm and (2) 30 nm of DVB-PFDA film. All durability tests were carried out in the same condition as the F-DLC samples.

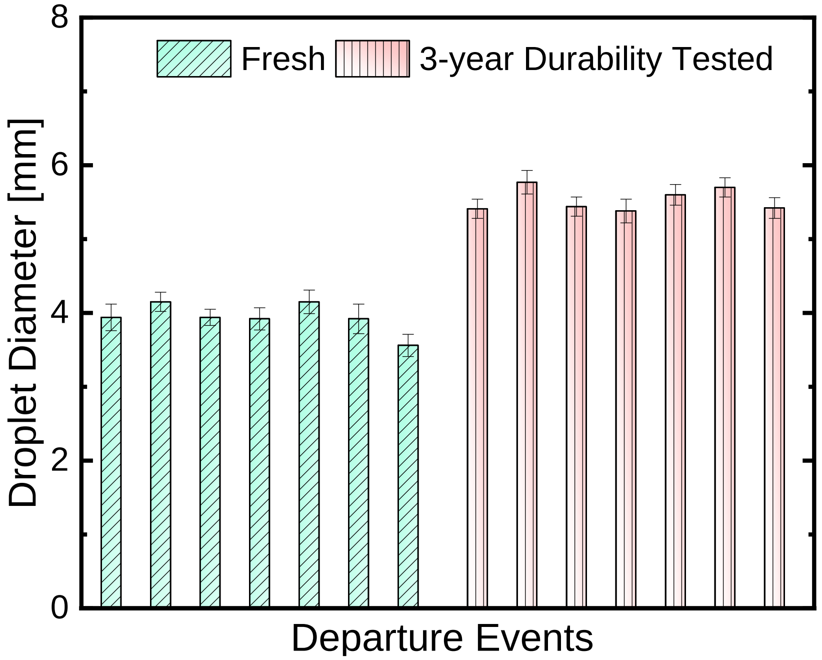

Supplementary Figure 8. Comparison of condensate departure size on a fresh and 3-year durability tested F-DLC surface deposited on a copper substrate. The error bars indicate the standard deviation, which was determined based on three independent measurements conducted for each data point. See Supplementary Note 7 for the details of the experiments.

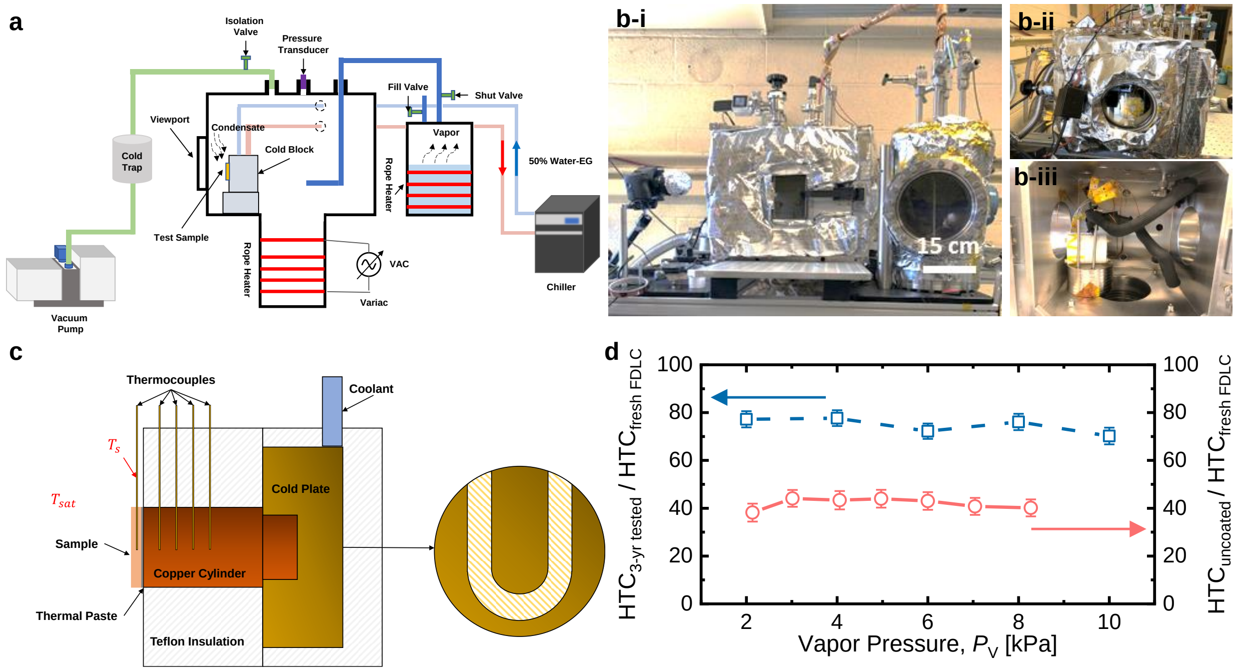

Supplementary Figure 9. (a) Schematic of the test setup used for the condensation heat transfer measurement of the flat samples. Optical Image of the vacuum chamber showing the (b-i) front, (b-ii) side, and (b-iii) internal view. (c) Schematic of the test section, showing the cold plate, thermocouples, and sample arrangement. Condensation experiments were conducted over a wide range of vapor pressure on both fresh and 3-year durability tested F-DLC coated copper coupons. (d) Normalized heat transfer coefficient (HTC) showing that the 3-year durability tested FDLC (HTC$_{\text{3-yr tested}}$) sample shows <30% reduction in condensation heat transfer coefficient compared to a freshly coated FDLC sample (HTC$_{\text{fresh FDLC}}$). This is due to the reduction of surface wettability and increment of the condensate size imposing higher thermal resistance to heat transfer. However, the HTC$_{\text{3-yr tested}}$ is still ~74% higher than uncoated copper sample (HTC$_{\text{uncoated}}$) which underwent filmwise condensation. Error bars were computed using the propagation of error (see Supplementary Note 4c). See Supplementary Note 7 for the details of the experiments.

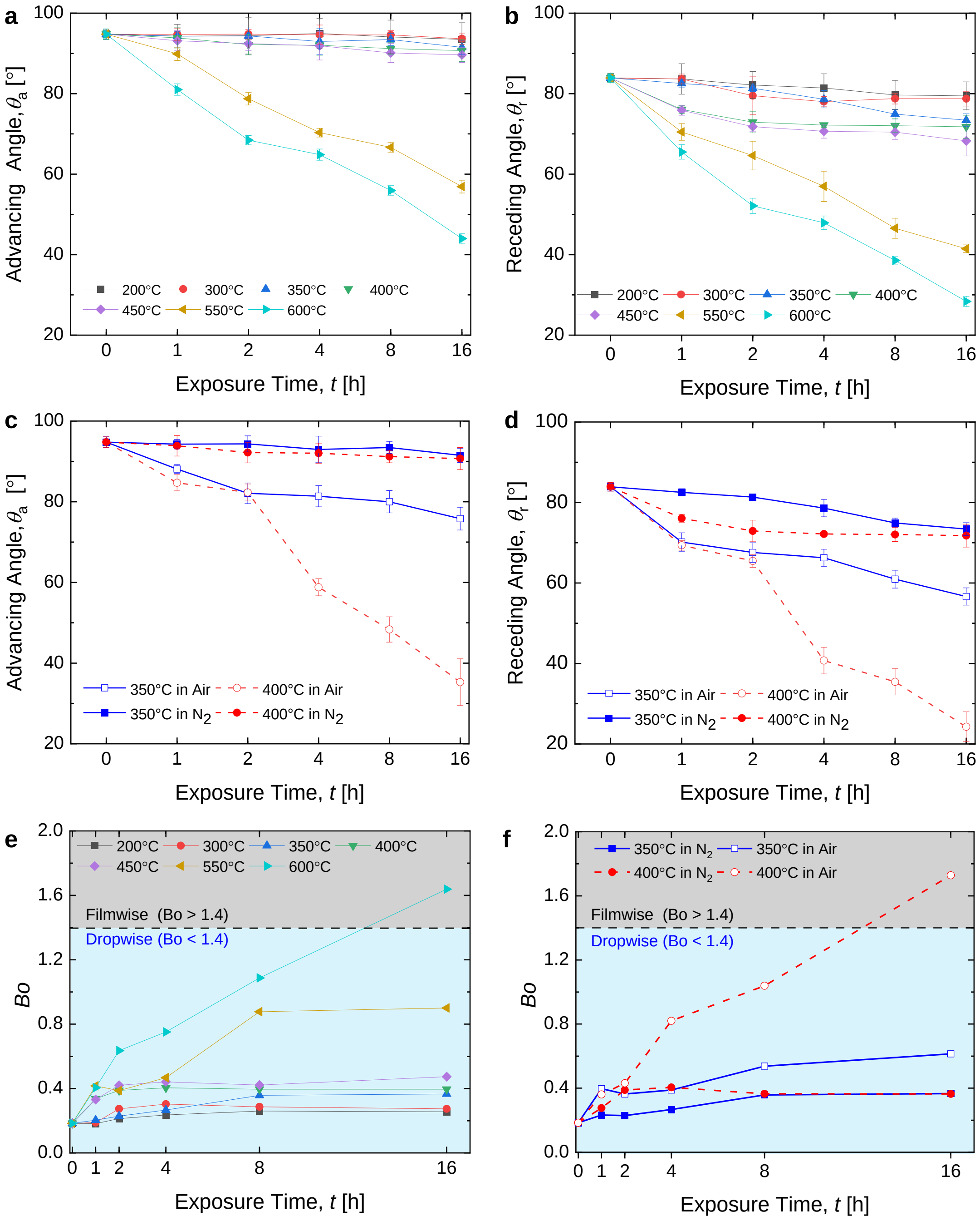

Supplementary Figure 10. Stability of the F-DLC coating in different thermal environments. (a) Advancing, and (b) receding DI water droplet contact angle of the F-DLC coating in inert environments (N_2 gas) for different temperatures showing good hydrophobic properties up top 450°C. (c)-(d) Comparison of F-DLC degradation process in air and N_2. Calculated Bond number (Bo) for the F-DLC coated surfaces in different thermal environments, showing a filmwise transition (Bo_{crit} > 1.4)[1] temperature in the (e) 600°C inert environment, and (f) 400°C non-inert environment. See Supplementary Note 8 for the details of the experiments. For (a-d), the error bars indicate the standard deviation, which was determined based on three independent measurements conducted for each data point.

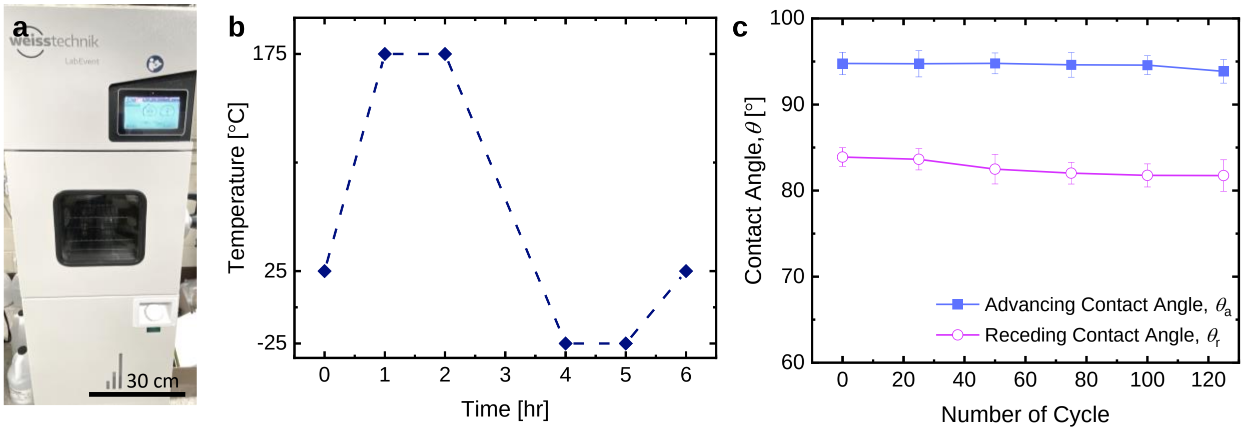

Supplementary Figure 11. Thermal cycling test of F-DLC coated samples. (a) Image of the cyclic chamber, (b) temperature profile of a single thermal cycle. (c) DI water contact angle of the F-DLC coated copper sample after exposure to different thermal cycles. See Supplementary Note 8 for the details of the experiments. The error bars indicate the standard deviation, which was determined based on three independent measurements conducted for each data point.

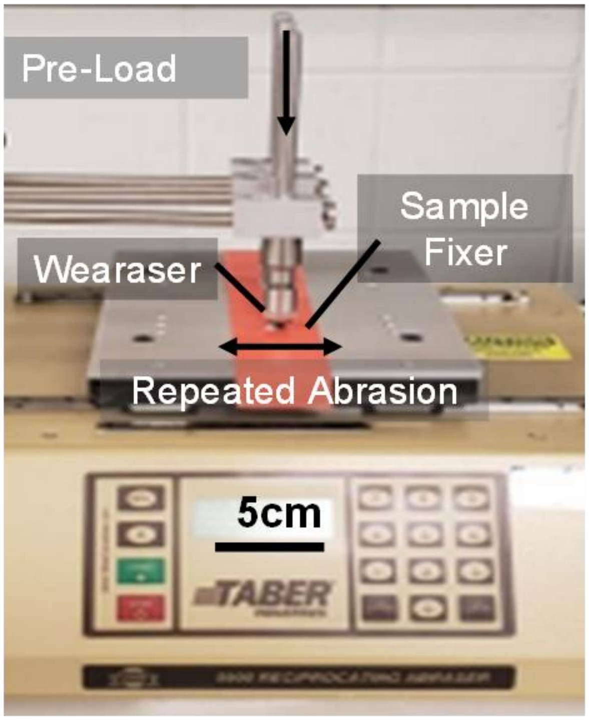

Supplementary Figure 12. Photograp of a reciprocating abrasion tester used to characterize the mechanical stability of the F-DLC coated samples. See Supplementary Note 8 for the details of the experiments.

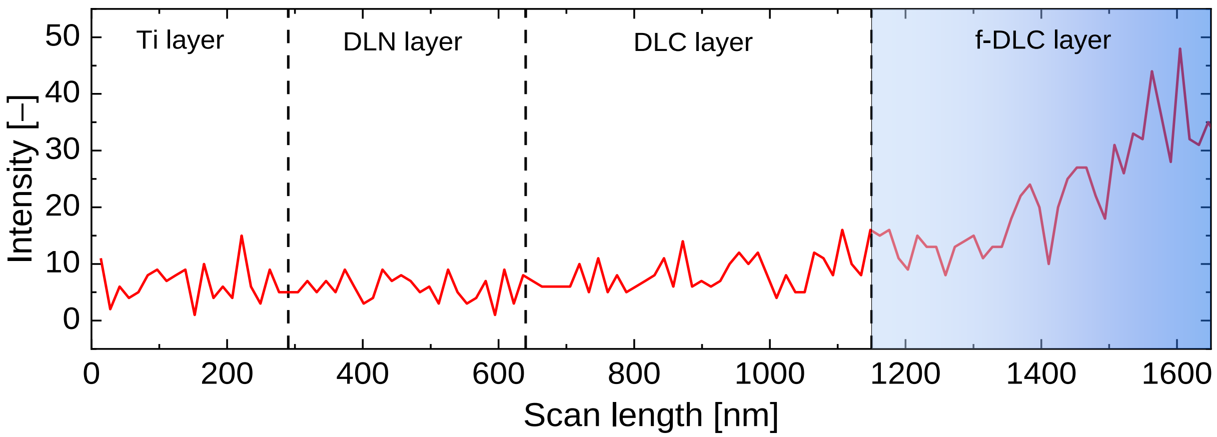

Supplementary Figure 13. EDS line scan result for Fluorine at a cross-section of the multi-layer F-DLC coating. The blue colored area indicates the f-DLC top layer. See Supplementary Note 8 for the details.

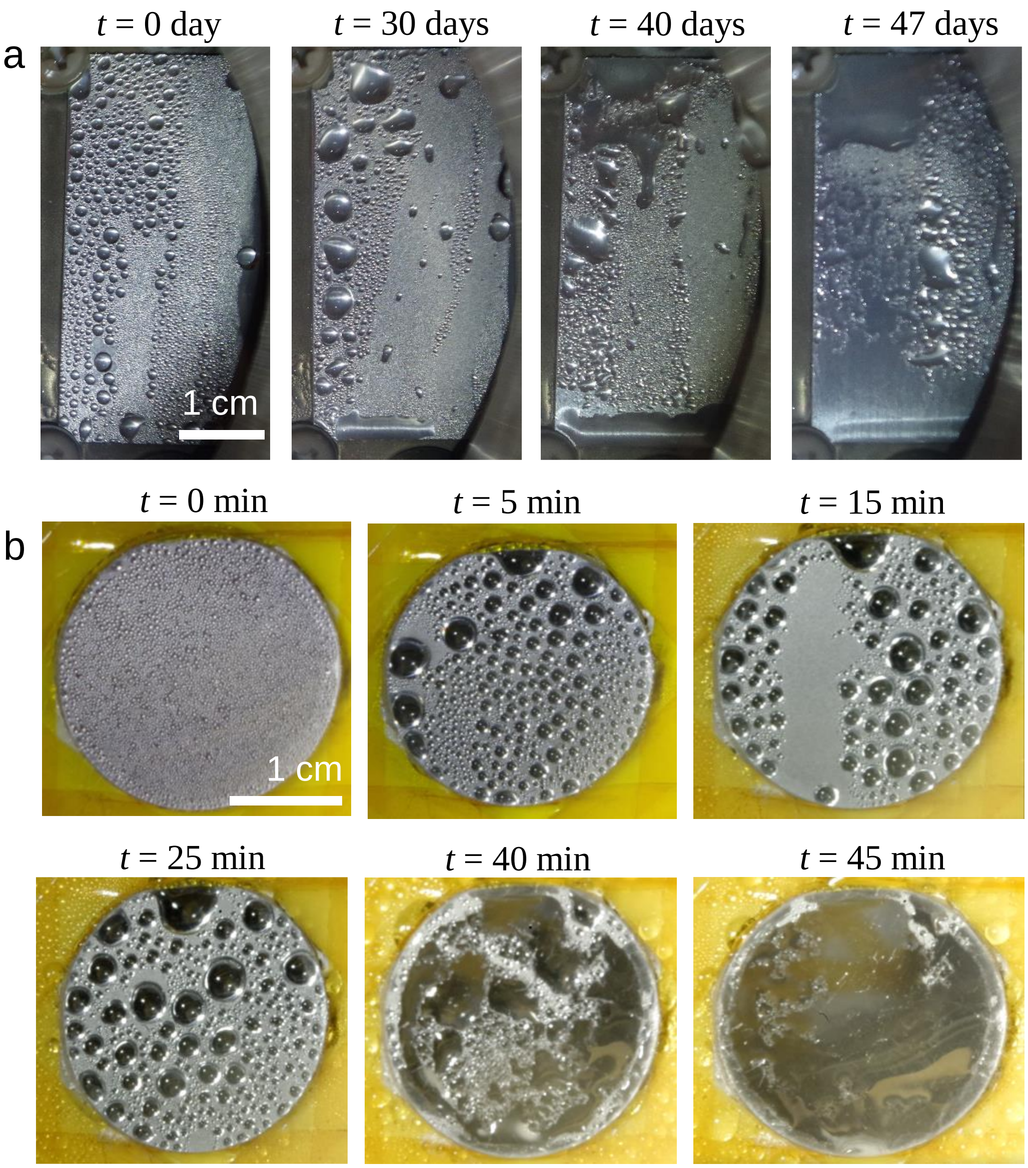

Supplementary Figure 14. Time-lapse photographs of durability tests during steam condensation on HTMS coated aluminum samples exposed to pure steam having (a) ~2 kPa vapor pressure and ~35°C vapor temperature, and (b) ~25 kPa vapor pressure and ~65°C vapor temperature. Surfaces exposed to high vapor pressure experience accelerated condensation rate, which results in much faster degradation of the hydrophobic chemistry as observed in (b). See Supplementary Note 9 for the details of the experiments.

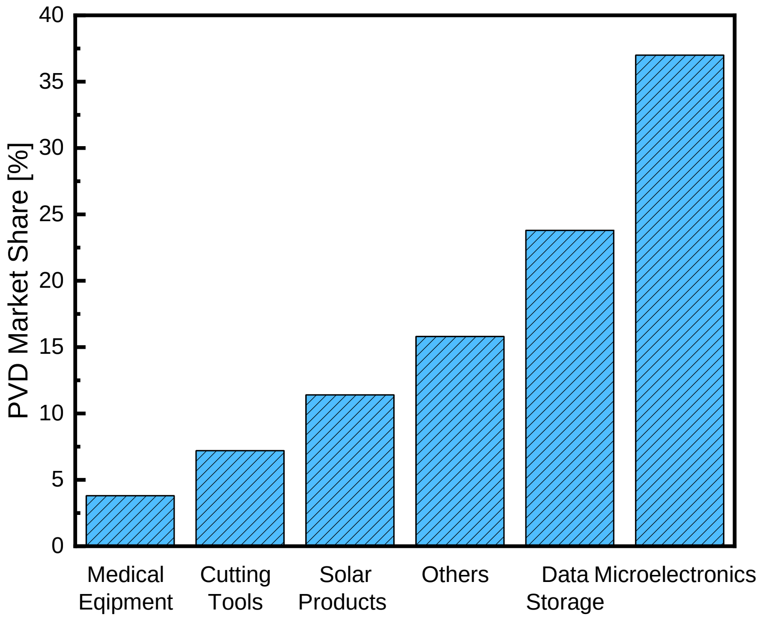

Supplementary Figure 15. PVD market share percentage by application for 2021. The rising demand for solar panels is expected to boost the demand for PVD coating during the forecast period.[2] See Supplementary Note 11 for the details.

Supplementary Tables

Supplementary Table 1. Summary of past studies on F-DLC conducted over the past 30 years. Studies are sorted focusing on studied substrates, fabricated layer thickness, number of layers, reported thermo-mechanical robustness and condensation durability. Here, Si = Silicon, SiC = Silicon Carbide, SiN= Silicon Nitride, SS = Stainless Steel, Cu = Copper, PMMA = Polymethyl Methacrylate, Al= Aluminum, KBr = Potassium bromide, PC = Polycabonate, PES = Polyethersulfone, Ti= Titanium. HTC= Heat Transfer Efficient, COF = Coefficient of Friction. Empty cells represent that the study did not report the data or did not conduct the experiments.

No	Year	Focus	Substrates	No. of layer/ Thickness	HTC ($kW.m^{-2}.K^{-1}$)	Thermo-mechanical robustness	Condensation Durability	Ref.
1	1993	Solid Lubrication	SiC, SS	1		Friction life maximum- 22 meters focusing on COF		3
2		Fabrication Parameters	Si	1				4
3	1994	Surface Modification & Properties	Si	1/1µm				5
4		Network Modification & Surface Energy	Si, Steel, Glass	1			Only angle reported	6
5	1995	Modification & Applications	Steel	3/5 µm				7
6	1997	Modification & Wettability	Si, Glass	1				8
7		Wear Resistance	Si	1				9
8	1998	Dropwise Condensation	Cu Disc	1/3.1 µm	Avg. 40 on flat surface		No instabilities of coating after 500 hours was mentioned	10
9		Dropwise Condensation	Cu Disc		Ratio of flat and tilted samples		Only condensation was performed on flat sample in atmospheric pressure at different orientation	11
10		Solid Lubrication	Si, SiN, SiC			Only wear rate reported		12
11		Dielectric Properties	Si					13
12	1999	Co-deposition & Hydrophobicity	Si, SS				Only angle reported	14
13	2000	Co-deposition & Optical Properties	Glass, PMMA	1/100nm				15
14	2001	Co-deposition	Si	1/1.3 µm			Only angle reported	16
15		Tribology	Steel	3/3 µm		Only wear rate reported		17
16		Heat Resistivity	Si	1/700 nm		Stable up to 300° C for 30 minutes		18
17	2002	Hydrophobicity	Si, Al	3/variable			Only angle reported	19
18		Hydrophobicity	Glass, Plastic	Variable			Only angle reported	20
19	2003	Structural and Mechanical Properties	Widia, Al, KBr	1/500nm-1 µm		Only hardness reported		21
20		Co-deposition and Properties	Si	2		Hardness and modulus reported	Surface energy measured	22
21	2004	Mechanical Properties	Si	1/variable		Hardness reported		23

22		Structural, mechanical, and hydrophobic properties	Si	2		Only hardness reported	Only angle reported	24
23		Surface modification and properties	Si	1		300 cycles of friction reported in air and vacuum	Only angle reported	25
24		Co-deposition and Dielectric Properties	Si	1				26
25	2005	Co-deposition and Anti-scaling	SS	1			Only surface energy reported	27
26		Co-deposition	Si	2/~500nm			Only angle reported	28
27		Antithrombogenicity	Si	1/50nm			Only angle reported	29
28		Co-deposition	Si	1/5nm			Surface energy reported	30
29	2006	Fabrication Parameters	Si, Quartz	1				31
30		Solid Lubrication	Steel	1/50nm				32
31		Surface Modification	Si	1/500nm			Only angle reported	33
32		Anti-thrombogenicity	Si	1/50nm				34
33		Anti-thrombogenicity	PC,SS	1/50nm				35
34	2007	Field Emission Properties	Glass, Si	1				36
35		Protein Adsorption and Platelet Activation	PC	1/50nm			Only angle reported	37
36		Nanoimprint Mold	Si	1/50nm				38
37			PMMA	1/1nm				39
38	2008		Si	1/300nm				40
39		Fabrication & Hydrophobicity	Si	1			Only angle reported	41
40		Anti-corrosion	NiTi	1				42
41	2009	Co-deposition & Properties	Si	2/1µm				43
42		Tribology	Si	1/800nm			Surface energy reported	44
43		Nanoimprint Mold	Si	1/200nm				45
44		Optical Properties	Si, Glass	1/500nm				46
45	2010	Biofouling	SS	2/1um			Only angle reported	47
46		Antibacterial Properties	SS	2/2um				48
47		Fabrication and Properties	Si	2/1um		Hardness reported	Surface energy reported	49
48	2011	Surface Modification	Si	1/1um			Only angle reported	50
49		Biofouling	Si	1/200nm				51
50	2012	Fabrication and Hydrophobicity	Si	1/100-200nm			Only angle reported	52
51		Biocompatibility	NiTi	1/500nm			Surface energy reported	53
52		Structure and Tribology	Si			10000 frictional cycles tested focusing on COF		54
53	2013	Thermo-mechanical properties	Glass plate, Hard metal	1/1.5um		Hardness reported after annealing for 16 hours		55
54		Anti-thrombogenicity	Si	1/50nm				56
55			PES membrane	1/3-6nm			Only angle reported	57
56		Tribology	Si			Hardness reported		58
57	2014	Modeling of FDLC						59
58	2016	Solid Lubrication & Wettability	SS				Only angle reported	60
59	2017	Antibacterial properties	PC	1/100-300nm			Surface energy and angle reported	61
60		Co-deposition & Corrosion Behavior	SS, Ti	3/3um				62

61	2020	Icing	SS	1/3µm			Dynamic angle measured	35
62		Anti-icing Performance	SS	1/3µm		325°C thermal stability. After 3000 abrasion cycles hydrophobicity reduced drastically	180 min of soaking in water degrades the coating	[63]
63		Enhanced HTC	Al	1/200nm	On flat surface in atmospheric pressure		30 days of wetting reported	[64]

Supplementary Table 2. Comparison of the attempted DLC coatings in this study. Details of different approaches are added in Supplementary Note 3.

**Coating**	$\boldsymbol{\theta}_{\mathbf{a}}$	$\boldsymbol{\theta}_{\mathbf{r}}$	$\boldsymbol{\Delta\theta}$ $(\boldsymbol{\theta}_{\mathbf{a}} - \boldsymbol{\theta}_{\mathbf{r}})$	**Note**
Single-layer DLC	89.0° ± 1.4°	53.1° ± 1.2°	≈36°	Not fluorinated
Single-layer DLC + Modification 1*	95.4° ± 1.7°	63.5° ± 5.5°	≈32°	Fluorinated (Modified)
Single-layer DLC + Modification 1**	95.5° ± 2.6°	60.7° ± 3.9°	≈35°	Fluorinated (Modified)
**Multi-layer F-DLC**	**99.1° ± 0.4°**	**86.2° ± 0.4°**	**≈13°**	**Fluorinated (co-deposition)**

Modification 1* and modification 2* refer to different surface fluorination techniques. Perfluoroheptane and Perfluoropentanol gases are used for modification 1 and modification 2, respectively.

Supplementary Table 3. Uncertainties corresponding to experimental measurements.

**Experimental Measurement**	**Uncertainty**
Cooling water temperature ($T_{\mathrm{out}}, T_{\mathrm{in}}$)	0.15°C + 0.12%
Saturated vapor pressure (P_{v})	1%
Saturated vapor temperature (T_{v})	$T_{sat}(1.01(P_v))-T_{sat}(P_v)$
Cooling water mass flow rate ($\dot{m}$)	1%
Sample surface area (A_{OD})	2%
Petukhov correlation heat transfer coefficient (h_{i})	6%

Supplementary Table 4. Condensation durability test samples and summary of test results.

Coating	Thickness	Substrate	θ_a	θ_r	$\Delta\theta$	Operation Time [days]	Durability
**F-DLC**	1.65 µm	Si	99.1° ± 0.4°	86.2° ±0.4°	13°	>1095	Good
		Polished Cu	98.4° ± 0.8°	82.7° ±1.1°	15°	>1095	Good
		Polished Al	99.0° ± 0.5°	85.3° ±0.8°	14°	>1095	Good
**HTMS**	2 nm	Polished Cu	120° ± 0.8°	94.6° ±1.2°	26°	<29	Poor
		Polished Al	111° ± 1.9°	71.1° ±0.5°	41°	<29	Poor

Supplementary Table 5. Surface property comparison of the F-DLC coated fresh sample and samples after exposure in the steam environment for 1095 days. The sample substrates were polished Si wafers.

**Coating property**	**As coated**					**After 1095 condensation days**				
$\boldsymbol{\theta_a/\theta_r}$	99.1°± 0.4° / 86.2°±0.4°					70.5°± 1.0° / 57.1°±1.3°				
**Chemical composition**	F %	O %	N %	C %	Si %	F %	O %	N %	C %	Si %
	9.5	27.5	2	38.9	24.1	4.9	33.7	0	38.5	22.9
**Thermal conductivity k [W.m^{-1}.K^{-1}]**	0.46 ± 0.05					0.42 ± 0.04				

Supplementary Table 6. Bond number (Bo) comparison between fresh F-DLC coated samples and condensation durability tested samples after 1095 days. Durability tested samples still maintain Bo smaller than the critical Bond number, $Bo_{crit} = 1.4$, ensuring dropwise condensation.

**Substrate**	**Before Test**			**After 1095 condensation days**		
	$\boldsymbol{\theta_a}$	$\boldsymbol{\Delta\theta}$	**Bo**	$\boldsymbol{\theta}_{\mathbf{a}}$	$\boldsymbol{\Delta\theta}$	**Bo**
Si	99.1° ± 0.4°	≈13°	0.2	70.5° ± 1.0°	≈14°	0.47
Polished Cu	98.4° ± 0.8°	≈15°	0.26	73.2° ± 0.6°	≈16°	0.51
Polished Al	99.0° ± 0.5°	≈14°	0.22	71.0° ± 1.1°	≈14°	0.49

Supplementary Table 7. Compiled performance parameters of different hydrophobic promoters reported in previous studies. Here θ_a, t, k, G, E, E_s refer to the advancing deionized water droplet contact angle, coating thickness, thermal conductivity, coating-substrate adhesion, Young's modulus, and surface energy, respectively. Empty cells represent that the study did not conduct or report the data.

No	Coating	θ_a [º]	*t* [nm]	*K* [$W.m^{-1}\cdot K^{-1}$]	*G* [$mJ.m^{-2}$]	*E* [GPa]	E_s $mJ.m^{-2}$]	Abrasion Resistance [# cycle]	Heat Transfer Coefficient [$kW.m^{-2}\cdot K^{-1}$]	Thermal Stability [ºC]	Condensation Durability [hours]	Ref.
1	Vitrimer	93 ± 3°	~10		1000	0.1	21	1		200	408^{+}	65
2	Armor-polymer	>170°	>10000					1000				66
3	PKFE nanocomposite	~160°	>50000					100				67
4	Candle soot	>160°	> 3000**							< 100		68, 69
4	Lipid-double layer	163± 2°	~100		800	4	10	1		200	8760^{+}	70, 71
5	Thick polymer	~90°	~60000								20000*	72
6	Nitrogen Ion /Chromium ion Implantation	60-90°	< 1000**								4000-5500 *	73, 74
7	Polyvinylidene chloride	< 90°**	10000								21568*	75
8	Few-layer graphene on nickel	~84°	<1**								25920*	76
9	Cu and gold-plated Cu with SAM	~110°	<2						~15		6480	77
10	SLIPS	~120± 3°	~2000							~150	1080	78
16	Fluroacrylic/ParyleneN/ParyleneD/No-Stik/Emralon333		3000/1000/1000/60000/13000								16000*/4000*/12000*/16000*/18000*	79
17	No-stik/Nerlox/Nedox/C6 Fluoroepoxy/BCE7Fluoroepoxy/Fluoroacrylate/Fluoroacrylic/Pepco61222/Isonel 472/Supttered PTFE/Parylene N/		60000/5000/5000/1000-10000/1000-10000/1000-10000/1000-10000/5000-10000/5000-10000/400/1000								22000*/4000*/1000*/22000*/20*/20*/20000*/2000*/24*/48*/4000*	80

19	Mixed monolayer (Stearic+octadecane), stearic acid, montanic acid										200*, 150*, 50 min*	81
21	Electroplated silver with cyanide coating		300								2400	82
22	Oleic acid on chromium surface										3000	83
23	Docosanoic acid SAM, electroless Ni-P-PTFE		0.3/ ~4500								168	84
24	N2 plasma ion implantation										480	85
25	hybrid organic–inorganic sol–gel silica coatings		250								2	86
26	Lubricant-Infused Vertical Graphene Nanoscaffolds		70								24	87
27	Superhydrophobic Copper oxide nanowires	159°	Micron scale								120	88
28	Hydrophilic SLIPS on CuO	32°	Micron scale								312	89
29	Copper oxide Nanograss		2000-4000								168	90
30	Reentrant structures (no coating)										504	91
31	Teflon coatings with adhesion promoter		67								386	92
33	dynamic ion-beam mixed implantation technique		~ 1000								1000	93, 94
34	Cerium Oxide (Rare earth oxides)		~300								100	95
35	16 low energy compounds tested										1730 (dibenzyl sulphide), 1350 (S-ethyl octadecyl xanthate), 1680 (SS-decamethylene di(octadecy1 xanthate), 3530 (glycerol tri-[ll-ethoxy(thiocarbonyl)thioundecanoate); 1850	96

											(dodecyltrisethyl thiosilane)	
36	Sulfide coated silver plated steel; Metals (Gold, Palladium, Rhodium); 1 micron parylene										12500 (sulfide data not consistent); 12500; 2000	[97]
38	Spray coated SAM (an aqueous silver nitrate solution and 1-dodecanethiol)										500	[98]
39	thermal stability of 1-octadecanethiol (ODT), 16-mercaptohexadecanoic acid (MHDA) and 1H,1H,2H,2H-perfluorodecanethiol (PFDT) SAMs on gold surfaces, and of 4-aminobutyltriethoxysilane (ABTES) and 1H, 1H, 2H, 2H-perfluorodecyltriethoxysilane (PFDS) assembled on hydroxylated silicon surfaces									110 (ODT), 145 (MHDA), 145 (PFDT), 250 (ABTES), 350 (PFDS)		[99]
42	Acrylonitrile butadiene styrene rubber (ABS) nanocomposites on Aluminum							1700		420		[100]
43	Laser microstructured titanium coated with waterborne fluoroacrylic polymer							200				[101]
44	Metal (copper)/polymer composite coating							10				[102]
45	Electroplating of zinc, nickel, and cobalt (Zn–Ni–Co electroplated coating)							50				[103]
46	Fluorinated polyurethane elastomer and polyhedral oligomeric silsesquioxane (FPOSS)							20000				[104]

47	Nanoparticles dispersed in PDMS resin matrix on copper							200				[105]
48	Porcupinefish-like structures (composite of tetrapodshaped ZnO and polydimethylsiloxane							1000				[106]
49	trichloro(1H,1H,2H,2H-perfluorooctyl)-silane and trichloro(octadecyl)-silane										460	[107]
50	Flexible polydimethylsiloxane polymer coating							10			40	[108, 109]
51	Bidentate PDMS film (b-PDMS), and Capped Bidentate PDMS Film (cb-PDMS)							5000			24	[110]
52	SLIPS (Copper oxide with Krytox GPL 103)										192	[111]
53	Polymer infused porous surfaces (PIPS)	~108°	5000						~130		4800	[112]
**54**	**F-DLC**	**~97± 1°**	**~1650**	**0.46**	**100000**	**~80**	**~24**	**5000**	**~45**	**350**	**26280[+]**	**This work**

* Condensation experiment conducted in the presence of non-condensable gases (NCGs).

** An estimated quantity done by the authors based on the available data in the paper.

+ Study did not observe and report coating failure at the end of the test

Supplementary Table 8. Comparison of thermal resistances during condensation of steam on a copper tube coated with FDLC of various thickness. For calculation details please refer to Supplementary Note 10.

Coating Thickness, h [μm]	R_{coating} [K.W^{-1}]	R_{wall} [K.W^{-1}]	$R_{\mathrm{convection}}$ [K.W^{-1}]	$R_{\mathrm{condensation}}$ [K.W^{-1}]
0.1	2.64×10^{-4}	5.36×10^{-4}	0.0118	0.024815
0.5	1.32×10^{-4}			0.024814
1.65	4.36×10^{-4}			0.024811
10	2.64×10^{-3}			0.024789
20	5.27×10^{-3}			0.024763

Considering a copper tube with D_{OD} = 9.53 mm, D_{ID} = 8.0 mm, L = 134.6 mm, and $k_{\mathrm{Cu}} = 386\ \mathrm{W.m^{-1} \cdot K^{-1}}$). And F-DLC coating with $\mathrm{k_{FDLC}} \sim 0.47\ \mathrm{W.m^{-1} \cdot K^{-1}}$. Also considering for a standard experimental condition, $\mathrm{h_i} \approx 25$ kW.m^{-2}.K^{-1}; $\mathrm{h} \approx 10$ kW.m^{-2}.K^{-1}.

Supplementary Table 9. A typical coating (CVD+PVD) costs per part for a durable decorative finish including all consumables. Consumables include electricity, metal "target" material used for coating, and process gases.[113] For details please see Supplementary Note 11.

PVD System	# Of Racks	Example Parts/Rack (6" part)	Example Parts/Batch (6" part)	Estimated PVD coating cost per PART	Estimated PVD coating cost per BATCH
Small (VT-1000i™ system)	6	16	96	$0.15	$14
Medium (VT-1500i™ system)	10	16	160	$0.10	$16
Large (VT-3000i™system)	16	16	256	$0.09	$22

Supplementary Notes

Supplementary Note 1. Crucial Requirements to Develop a Robust Hydrophobic Coating

A robust hydrophobic coating for power system applications should have a scalable and substrate independent fabrication method (Supplementary Figure 1). The coating should also have low thermal resistance (<5 μm thick with high thermal conductivity) to condensation heat transfer and should sustain elevated thermo-mechanical environments and humid environments for long times (years) (Supplementary Figure 1).

Supplementary Note 2. Potential of DLC and Summary of Previous Studies

The first report on hard amorphous carbon films (also known as diamond like carbon, DLC) was published in 1953 by Heinz Schmellenmeier. About 20 years later, worldwide intensive research activities initiated on DLC. In the following years, the number of publications increased continuously and the importance for industrial applications became more and more evident. Several deposition techniques were applied to prepare hydrogenated (a-C:H), non-hydrogenated (ta-C) amorphous carbon, metal containing a-C:H:Me, and non-metal containing a-C:H:X coatings. In parallel the structure and deposition mechanisms of DLC coatings were extensively studied. An essential obstacle for a broad industrial application was the high compressive stress level in a-C:H (DLC) films causing delamination and limiting the film thicknesses. There are a number of strategies to reduce the compressive stress, it effects, and to increase the adhesion. It is found that for Tetrahedral amorphous carbon (ta-C), an annealing the film at a temperature > 500°C reduces the stress[114, 115] . However, this method is not useful for a-C:H, because at such high temperature the hydrogen is lost, so the film is no longer diamond-like. To overcome this obstacle, metal inclusion in DLC (Me-DLC, today abbreviated as a-C:H:Me) was proposed[116], with metal based intermediate layer systems most adhesion problems could be solved satisfactorily and thus from the mid-1990s the pre-conditions for a broad application especially in the automotive industry were given. However, later it was realized that Me-DLC has higher wear rate and poor mechanical stability[117]. In the mid-1990s, it was discovered that incorporation of non-metal elements into the a-C:H film can be a solution to this. Moreover, it alters the surface energies of a-C:H coatings to lower (X: F, Si, Si+O) and also to higher (B, N, O) values[7] . Thus, it became possible to combine the high mechanical stability of diamond-like carbon with low surface energies comparable with those of the well-known hydrophobic material polytetrafluorethylene (PTFE — surface energy about 19 mN/m, hardness <1 GPa). In a study, Grischke et al.[6, 118]

reported low surface energies of modified a-C:H:Si (31 $mN.m^{-1}$, 10–15 GPa), a-C:H:Si:O (24 $mN.m^{-1}$, 7–10 GPa) and a-C:H:F (20 $mN.m^{-1}$, 2 GPa) coatings. For the corresponding pure a-C:H coatings the surface energy was 41 $mN.m^{-1}$ and the hardness of 20–30 GPa. Other method to reduce stress are forming multi-layers, the high potential of DLC based multilayers was confirmed by later publications in the 2000s[119, 120, 121] . Multi-layer DLC is more stable than monolayer DLC due to reduced stress. Inclusion of F also improves the flexibility of DLC, which is helpful in application where deformation, movement (tubes, guidewires) exists[37]. The smooth, amorphous-no grain boundaries, fluorinated diamond like carbon (F-DLC) film gives pin-hole-free continuous film, as DLC can nucleates wherever the carbon ions impact the surface[122], this homogenous film property should yield to long-term corrosion barrier to water. F-DLC is a good solid lubricant, though fluorine in DLC reduces the internal stress, hardness and Young's modulus (lower than pure DLC), but wear resistance increased, coefficient of friction (COF) reduces, adhesion increases, and surface energy decrease[24, 43, 55, 58, 60]. In the past 30 years, there were extensive research on F-DLC focusing on mostly tribological, medical, mechanical, wettability, and dielectric properties improvement focusing on various fabrication method, recipe modification and doping, with limited focus on substrate versatilities, long-term sustainability of enhanced properties (Supplementary Table 1). As shown in Supplementary Table 1, the majority of past studies only report the wettability change of the developed coating. Few studies have demonstrated potential applications of doped-DLC for heat exchanger surfaces for anti-scaling[123] and condensation on flat surface for short durations (<1 month)[10]. Recent studies showed anti-icing[63], and heat transfer enhancement of FDLC[64]. However, in these studies, crucial parameters for robust hydrophobicity (Supplementary Figure 1) were not considered, implemented, or tested/reported. Interestingly, no other studies to date have demonstrated or reported robust hydrophobicity

covering scalable and substrate versatile fabrication methods using the design strategies for strong adhesion, thermo-mechanical robustness, and long-term durability to steam condensation with enhanced heat transfer performance.

Supplementary Note 3. Pathway from DLC to Multi-layer Durable F-DLC Development

Prior to developing the F-DLC formulation, we began by investigating the condensation performance of traditional single layer fluorine free DLC coatings deposited on copper substrates by plasma assisted physical vapor deposition (PACVD). The advancing and receding contact angles of water on fluorine-free DLC coatings were 89.0° ± 1.4° and 53.1° ± 1.2°, respectively (see Supplementary Figure 2ia). Due to the high contact angle hysteresis of fluorine-free DLC (≈36°), non-sustainable dropwise condensation ensued (Supplementary Figure 2ib). To make the DLC more favorable for dropwise condensation, we then reduced the surface energy by fabricating fluorinated diamond like carbon (F-DLC). There are two strategies to develop F-DLC: (1) by surface modification with fluorine, or by (2) co-deposition of fluorine. We started by using surface modification with fluorinated gases (Perfluoroheptane, Perfluoropentanol). As shown in Supplementary Table 2, compared to fluorine free DLC, surface modified DLC has higher contact angles. However, the contact angle hysteresis is still high leading to undesirable condensation performance. To develop a low hysteresis and durable F-DLC coating we started to study the degradation mechanism and required properties to overcome the degradation, and finally, developed the multi-layer co-deposited F-DLC coating (Supplementary Figure 2ii).

Supplementary Note 4. Environmental Chamber Setup for Heat Transfer Characterization

Supplementary Note 4a. Chamber Setup

The complete description of the experimental setup (Supplementary Figure 3) with all components is discussed in detail in previous work.[124, 125] In brief, the individual components are discussed below.

Main Chamber. The custom environmental chamber used for this work was designed by Gladwin Tank Manufacturing to withstand 5066 kPa of internal pressure and is ASME code stamped. The chamber has an internal diameter of 0.305 m, a length of 0.559 m, and is sealed at each end with 44.5 mm thick flanges. Both flanges are sealed with neoprene rubber gaskets. The front flange rests on a sliding mechanism so that the chamber can be easily opened. Both flanges have several leak-tight pass-through ports that allow instruments and fluid lines into the chamber. Viewports are installed on the chamber walls to allow for visual access. The chamber was specifically designed to accommodate both high pressure operation as well as vacuum operation.

Vacuum Pump. Before each experiment, the chamber is evacuated with an Adixen vacuum pump (Model Alcatel 2005) to remove non-condensable gases. A liquid nitrogen cold trap was incorporated into the pump-down line from the chamber to the vacuum pump to remove any moisture from the air, which improves the quality of the vacuum and maintains the reliability of the pump.

Vapor Generation. Water vapor is generated in a smaller stainless-steel vessel (8” OD Standard CF Tee, Kurt J. Lesker). The vessel is wrapped with three independently controlled 120 W tape heaters and is insulated to limit losses to the environment. The temperature of the fluid is monitored using a T-type thermocouple inserted directly into the liquid with a feedthrough connection on the top flange. For safety, a pressure relief valve set at 300 kPa is connected to the vapor generator.

Test Sample. The test samples used in this study are tubes with an outer diameter of 9.53 mm and a length of 134.6 mm. The diameter was chosen to maintain high heat transfer coefficients on the internal surface. A chilled water flow loop is used to cool the test samples to promote condensation on the F-DLC coated or bare copper surfaces. All auxiliary tubing inside the chamber is insulated to prevent condensate formation on its surface.

Measurements and Data Acquisition (DAQ). The water flow rate was measured using an electromagnetic flow meter (Part #FMG93, Omega) and the inlet and outlet temperatures into the test section were measured using class A resistance temperature detectors, or RTDs (Part #AT-PX1123Y-LR4S1T2T, ReoTemp). During each test, the pressure of the working fluid (water) was measured using two pressure transducers (Baratron 728A and MicroPirani 925 supplied by MKS Instruments). A detailed reasoning for using data from the Baratron 728A over the classical MicroPirani 925 is listed in a previous work.[125] The vapor temperature and the chamber and test section wall temperatures are monitored with T-type thermocouples (Part #SCPSS-032, Omega).

All measurements were collected with a data acquisition system (DAQ) (PXIe 1073, National Instruments) and analyzed with LabVIEW. The PXIe system was specifically chosen due to its low DAQ error, enabling high fidelity measurements.

Finally, visual recordings of the condensation process were captured with a high-speed camera (Phantom v7.1, Vision Research) and a digital SLR camera (Pentax K-50).

Supplementary Note 4b. Experimental Procedure

The experimental procedure for steady-state vapor condensation within the chamber is discussed in detail in our previous work.[125] Here we discuss in brief the initial chamber preparation step and the steady state condensation operation.

A set of strict procedures were followed to maintain consistency across experiments and to ensure repeatable results. The test sample was connected to the coolant line using a T-joint Swagelok tube fitting and all connections were checked before the chamber was sealed to ensure there were no leaks. Once the environmental chamber front flange door was sealed, the vapor generator was completely filled with approximately 4.5 liters of deionized (DI) water and heated. After the DI water boiled (>95°C) for at least 10 minutes, the vacuum-pump-down procedure of the environmental chamber was initiated.

During the vacuum-pump-down, the pressure inside the chamber was monitored using both of the installed pressure transducers. This process took 30-45 minutes in order to achieve the target vacuum conditions. The environmental chamber was evacuated to a pressure of $P < 1 \pm 0.025$ Pa with a leak rate of 0.1 Pa.min^{-1} after chamber isolation. Evacuation eliminated noncondensable gases (NCGs) which add diffusional resistance to condensation heat transfer.[126]

The final step in the startup procedure was to initiate the cooling water flow loop. The Neslab System 3 water pump reservoir was filled and turned on to the maximum recirculating pressure valve setting, giving a flow rate of $\approx 8 \pm 0.2$ L.min^{-1}. The relatively high flow rate of 8 $\text{L.min}^{-1}$was chosen to ensure a well-mixed highly turbulent flow with Reynolds number of $\text{Re}_{\text{d}_{\text{ID}}} = \rho_{\text{w}} \bar{U} \text{d}_{\text{ID}} / \mu_{\text{w}} = 24{,}000$. This helped in maximizing the internal heat transfer coefficient and therefore reduce the overall condensation thermal resistance. The reservoir temperature was set at 7 ± 1°C and the coolant was circulated through the flow loop until the test section inlet temperature (T_{in}) ranged between 7 to 8°C, and was maintained for at least 5 minutes to confirm steady state operation.

In summary, the following conditions were met before injecting vapor to the environmental chamber: 1) the liquid inside the vapor generator must reach the boiling point and generate vapor for at least 10 minutes, 2) the inlet coolant temperature must be between 7°C and 8°C for at least 5 minutes, and 3) the chamber pressure must be below 1 Pa. Once these conditions were reached,

the valve in the vacuum-line was closed and the vacuum pump turned off. The vent valve on the vapor generator was closed and the valve connecting the vapor generator to the environmental chamber was gradually opened to allow vapor inside the chamber until it reached a desired pressure. All thermocouples, RTDs and pressure transducer readings were recorded over a 10-minute period to obtain steady state condensation measurements.

Supplementary Note 4c. Heat Transfer Calculations and Error Analysis

<u>*Overall heat transfer coefficient ($\bar{U}$).*</u> The overall condensation heat transfer rate (Q) was calculated using an energy balance on the cooling water flowing inside the tube, as shown in Equation S1:

$$Q = \dot{m}c_{\mathrm{p}}(T_{\mathrm{out}} - T_{\mathrm{in}})\ , \tag{S1}$$

where Q is the overall condensation heat transfer rate, $\dot{m}$ is the cooling water mass flow rate, c_{p} is the liquid cooling water specific heat, and T_{out} and T_{in} are the outlet and inlet temperatures, respectively. The overall heat transfer rate (Q) was then balanced with the overall heat transfer coefficient, $\bar{U}$ as:

$$\dot{m}c_{\mathrm{p}}(T_{\mathrm{out}} - T_{\mathrm{in}}) = \bar{U}A_{\mathrm{o}}\Delta T_{\mathrm{LMTD}}\ , \tag{S2}$$

where A_{o} is the tube outer surface area ($A_{\mathrm{o}} = \pi d_{\mathrm{OD}}L$, where d_{OD} = 9.35 mm, L = 134.62 mm) and ΔT_{LMTD} is the log mean temperature difference defined by:[127]

$$\Delta T_{\mathrm{LMTD}} = \frac{(T_{\mathrm{v}} - T_{\mathrm{in}}) - (T_{\mathrm{v}} - T_{\mathrm{out}})}{\ln\left[\frac{(T_{\mathrm{v}} - T_{\mathrm{in}})}{(T_{\mathrm{v}} - T_{\mathrm{out}})}\right]}\ , \tag{S3}$$

where T_{v} is the temperature of the surrounding saturated vapor inside the chamber ($T_{\mathrm{v}} = T_{\mathrm{sat}}(P_{\mathrm{v}})$). The overall heat transfer coefficient ($\bar{U}$), which is only a function of experimentally obtained parameters, can thus be calculated as:

$$\bar{U} = \frac{\dot{m}c_{\mathrm{p}}(T_{\mathrm{out}} - T_{\mathrm{in}})}{A_{\mathrm{o}}\Delta T_{\mathrm{LMTD}}}\ . \tag{S4}$$

Internal convection heat transfer coefficient (h_i). The calculated $\bar{U}$ is a measure of the overall heat transfer performance from the vapor to the cooling water. It includes the convective resistances on the inner and outer walls and the conductive resistance through the copper wall and hydrophobic coating. Further calculations were performed to isolate the thermal resistance on the outer wall to quantify the condensation heat transfer coefficient, h_c, as measured from the vapor to the tube outer surface.

To extract h_c, the conductive resistance was calculated using the thermal conductivity of hollow conductive cylinder assembly and the internal resistance was calculated by estimating the internal heat transfer coefficient. The water-side heat transfer coefficient (h_i) was estimated by the Petukhov correlation (Equations S5-S7), which is relevant to the coolant flow conditions and has an accuracy of approximately 6%:[128]

$$h_i = \frac{k_i}{d_{ID}} \frac{\left(\frac{f}{8}\right)\mathrm{RePr}}{\left(1.07 + 12.7\left(\frac{f}{8}\right)^{\frac{1}{2}}\left(\mathrm{Pr}^{\frac{2}{3}} - 1\right)\right)} \left(\frac{\mu_b}{\mu_s}\right)^n . \qquad \text{(S5)}$$

$$\mathrm{Re} = \frac{\rho v d_{ID}}{\mu} . \qquad \text{(S6)}$$

$$f = [0.79 \ln(\mathrm{Re}) - 1.64]^{-2} . \qquad \text{(S7)}$$

In Equations S5 to S7, f represents the pipe friction factor, Re is the cooling water Reynolds number, Pr is the cooling water Prandtl number, ρ is the cooling water density, k_i is the cooling water thermal conductivity, and μ_b and μ_s are the cooling water dynamic viscosities at the bulk and tube wall temperatures, respectively.

Thermal resistances. The overall heat transfer performance from vapor-to-cooling water can be expressed as a series sum of four individual thermal resistances: the internal convective resistance R_{conv}, the radial conduction tube wall resistance R_{wall}, the conductive resistance through the coating, R_{coating}, and condensation heat transfer thermal resistance $R_{\mathrm{condensation}}$, as shown in Supplementary Figure 4.

Considering the coating thickness of the F-DLC as h, the thermal resistance of the coating on a tube with length, L is:

$$R_{\mathrm{coating}} = \frac{\ln\left(\frac{d_{\mathrm{OD}} + h}{d_{\mathrm{OD}}}\right)}{2\pi L k_{\mathrm{F-DLC}}}, \tag{S8}$$

where d_{OD} is the outer diameter of the bare copper tube and $k_{\mathrm{F-DLC}}$ is the thermal conductivity of the F-DLC multilayer coating. Similarly, the conductive tube wall resistance can be calculated as:

$$R_{\mathrm{wall}} = \frac{\ln\left(\frac{d_{\mathrm{OD}}}{d_{\mathrm{ID}}}\right)}{2\pi L k_{\mathrm{Cu}}}, \tag{S9}$$

where d_{OD} and d_{ID} are the outer and inner bare tube diameters, respectively and k_{Cu} is the thermal conductivity of multipurpose copper. The internal convection resistance and external condensation resistance can be calculated as:

$$R_{\mathrm{conv}} = \frac{1}{h_{\mathrm{i}} \pi d_{\mathrm{ID}} L}. \tag{S10}$$

$$R_{\mathrm{condensation}} = \frac{1}{h_{\mathrm{c}} \pi d_{\mathrm{OD}} L}. \tag{S11}$$

Condensation heat transfer coefficient (h_c).

The overall heat transfer coefficient can be expressed as Equation S12 by considering all four thermal resistances in series:

$$\frac{1}{\overline{U}A_{\mathrm{o}}} = R_{\mathrm{conv}} + R_{\mathrm{wall}} + R_{\mathrm{coating}} + R_{\mathrm{condensation}}\,, \tag{S12}$$

where A_{o} is the tube outer surface area ($A_{\mathrm{o}} = \pi d_{\mathrm{OD}} L$). Knowing h_{i}, a closed form solution can be obtained for h_{c} by combining all the relevant thermal resistances (internal convection and radial conduction through the tube wall and coating):

$$h_{\mathrm{c}} = \left(\frac{1}{\overline{U}} - A_{\mathrm{o}}(R_{\mathrm{conv}} + R_{\mathrm{wal}l} + R_{\mathrm{coating}})\right)^{-1}. \tag{S13}$$

Tube surface temperature (T_{surf}). The tube surface outer temperature, T_{surf}, was used to calculate the supersaturation for each test condition. The outer wall temperature was calculated using the total heat transfer rate and the conductive and water-side convective thermal resistances, as shown in Equation S14:

$$Q = \frac{T_{\mathrm{surf}} - T_{\mathrm{avg}}}{\dfrac{1}{h_{\mathrm{i}}A_{\mathrm{i}}L} + \dfrac{\ln\left(\dfrac{d_{\mathrm{OD}}}{d_{\mathrm{ID}}}\right)}{2\pi L k_{\mathrm{t}}}}, \tag{S14}$$

where $T_{\mathrm{avg}} = (T_{\mathrm{out}} + T_{\mathrm{in}})/2$. Rearranging Eq. S14, the tube surface temperature can be calculated as:

$$T_{\mathrm{surf}} = T_{\mathrm{avg}} + \dot{m}c_{\mathrm{p}}(T_{\mathrm{out}} - T_{\mathrm{in}})\left[\frac{1}{h_{\mathrm{i}}A_{\mathrm{i}}L} + \frac{\ln\left(\dfrac{d_{\mathrm{OD}}}{d_{\mathrm{ID}}}\right)}{2\pi L k_{\mathrm{Cu}}}\right]. \tag{S15}$$

Finally, the supersaturation, S, defined as the ratio of the vapor pressure to the saturation pressure corresponding to the tube sample surface temperature is given by:

$$S = \frac{P_{\mathrm{v}}}{P_{\mathrm{sat}}(T_{\mathrm{surf}})}. \tag{S16}$$

Error analysis. The uncertainty of the overall heat transfer coefficient, $\overline{U}$, was calculated by propagating the instrument uncertainty of each measured variable (Supplementary Table S3), as shown in Equation S17:

$$E_{\overline{\mathrm{U}}} = \overline{U}\sqrt{\left(\frac{E_{\dot{\mathrm{m}}}}{\dot{m}}\right)^2 + \left(\frac{E_{(T_{\mathrm{out}}-T_{\mathrm{in}})}}{(T_{\mathrm{out}} - T_{\mathrm{in}})}\right)^2 + \left(\frac{-E_{\mathrm{A}}}{A}\right)^2 + \left(\frac{-E_{\Delta T_{\mathrm{LMTD}}}}{\Delta T_{\mathrm{LMTD}}}\right)^2}\,. \tag{S17}$$

As the condensation heat transfer coefficient, h_{c} is a product of powers, the error is determined as a function of the first partial derivatives of h with respect to its components:

$$E_{h_c} = h_c\sqrt{\left(\frac{\partial h_{\mathrm{c}}}{\partial h_{\mathrm{i}}}\frac{E_{\mathrm{h_i}}}{h_{\mathrm{i}}}\right)^2 + \left(\frac{\partial h_{\mathrm{c}}}{\partial \overline{U}}\frac{E_{\overline{\mathrm{U}}}}{\overline{U}}\right)^2}\,, \tag{S18}$$

$$\frac{\partial h_c}{\partial h_{\mathrm{i}}} = \frac{-(A_{\mathrm{o}}/A_{\mathrm{i}})\overline{U}^2}{(h_{\mathrm{i}} - (A_{\mathrm{o}}/A_{\mathrm{i}})\overline{U} - R_{\mathrm{t}}A_{\mathrm{o}}\overline{U}h_{\mathrm{i}})^2}\,, \tag{S19}$$

$$\frac{\partial h_c}{\partial \overline{U}} = \frac{{h_{\mathrm{i}}}^2}{(h_{\mathrm{i}} - (A_{\mathrm{o}}/A_{\mathrm{i}})\overline{U} - R_{\mathrm{t}}A_{\mathrm{o}}\overline{U}h_{\mathrm{i}})^2}\,, \tag{S20}$$

where $R_{\mathrm{t}}$is the thermal resistance of the tube wall given by:

$$R_{\mathrm{wall}} = \frac{\ln\left(\frac{d_{\mathrm{OD}}}{d_{\mathrm{ID}}}\right)}{(2\pi k_{\mathrm{Cu}})}\,. \tag{S21}$$

Supplementary Note 4d. Dropwise Condensation Model

To calculate the overall condensation heat transfer, we first calculate the heat transfer through a water droplet. Then, we multiply the individual droplet heat transfer with the droplet distribution density to get the overall condensation heat flux. To calculate the droplet heat transfer, we studied the thermal resistance network. For a droplet residing on a surface, the dominating thermal resistances are the conduction resistance within the droplet, the surface conduction thermal resistance (coating), and the interfacial thermal resistance at the liquid-vapor interface. The conduction resistance is determined by following our previous study.[129] Due to the sufficiently small size of condensing droplets, convection inside of the droplet is low and is neglected.[130] Here we consider a droplet with radius R on a plain surface which is coated with F-DLC, as shown in Supplementary Figure 5. The contact angle θ of the F-DLC coated hydrophobic surface is assumed to be fixed regardless of the droplet size, surface temperature and vapor temperature. Knowing the vapor saturation temperature, T_{sat} and F-DLC coated tube surface temperature, T_{surf}, the heat transfer rate Q can be calculated by considering all thermal resistances.

Considering the droplet nucleation site density, N_s,[131] the effective coalescence radius of the droplets, R_e above which droplets starts to coalesce can be calculated by:

$$R_{\text{e}} = 1/\sqrt{4N_{\text{s}}}\,. \tag{S22}$$

Droplet critical nucleation radius ($R_{\min}$) was calculated by:[130]

$$R_{\min} = \frac{2T_{\text{sat}}\sigma}{h_{\text{fg}}\rho(T_{\text{sat}} - T_{\text{surf}})}\,. \tag{S23}$$

where $\sigma, \rho,$ and h_{fg} are the liquid-vapor surface tension, density, and latent heat of vaporization of water, respectively.

The heat transfer rate through a single droplet with radius R is:

$$q(R) = \frac{\pi R^2 (T_{\mathrm{sat}} - T_{\mathrm{surf}}) \left(1 - \frac{R_{\mathrm{min}}}{R}\right)}{\frac{1}{2h_{\mathrm{i}}\left(1 - \cos\theta_{\mathrm{a}}^{\mathrm{app}}\right)} + \frac{R\theta_{\mathrm{a}}^{\mathrm{app}}}{4k_{\mathrm{w}}\sin\theta_{\mathrm{a}}^{\mathrm{app}}} + \frac{\delta}{k_{\mathrm{HC}}\sin^2\theta_{\mathrm{a}}^{\mathrm{app}}}} \tag{S24}$$

where, k_{w}, and k_{HC} are thermal conductivity of water and F-DLC, respectively. Here $\theta_{\mathrm{a}}^{\mathrm{app}}$ is the apparent advancing contact angle of condensate droplets, h_{i} is the interfacial heat transfer coefficient and is determined by the Schrage equation:[129, 130, 132]

$$h_{\mathrm{i}} = \frac{2\alpha}{2 - \alpha} \frac{1}{\sqrt{2\pi R_{\mathrm{g}} T_{\mathrm{sat}}}} \frac{h_{\mathrm{fg}}^2}{v_{\mathrm{g}} T_{\mathrm{sat}}}\,, \tag{S25}$$

where a is the accommodation coefficient ranging from 0 to 1, representing the ratio of vapor molecules that will be captured by the liquid phase to the total number of vapor molecules reaching the liquid phase, and R_{g} is the specific gas constant of vapor.

To calculate the overall condensation heat flux we combined the individual droplet heat transfer with the total number distribution of droplets on the condensing surface. For a superhydrophobic surface, a droplet distribution theory to account for the fraction of droplets on the surface of a given radius $\boldsymbol{R}$ for surfaces undergoing shedding and jumping is used. For small droplets ($\boldsymbol{R} \leq \boldsymbol{R_e}$), the size distribution $\boldsymbol{n(R)}$ is determined by:[133]

$$n(R) = \frac{1}{3\pi R_{\mathrm{e}}^3 \hat{R}} \left(\frac{R_{\mathrm{e}}}{\hat{R}}\right)^{-\frac{2}{3}} \frac{R(R_{\mathrm{e}} - R_{\mathrm{min}})}{R - R_{\mathrm{min}}} \frac{A_2 R + A_3}{A_2 R_{\mathrm{e}} + A_3} \exp(B_1 + B_2)\,, \tag{S26}$$

where, where $\hat{R}$ is the effective maximum droplet radius. For large droplets ($R \geq R_{\mathrm{e}}$) growing due to coalescence, the droplet distribution $N(R)$ is determined from:[134]

$$N(R) = \frac{1}{3\pi R_e^2 \hat{R}} \left(\frac{R_e}{\hat{R}}\right)^{-\frac{2}{3}} . \tag{S27}$$

The parameters A_1, A_2, A_3, B_1, B_2 are constants defined as:[129]

$$A_1 = \frac{\Delta T}{h_{fg} \rho_w \left(1 - \cos\theta_a^{app}\right)^2 \left(2 + \cos\theta_a^{app}\right)} , \tag{S28}$$

$$A_2 = \frac{\theta_a^{app}}{4 k_w \sin\theta_a^{app}} , \tag{S29}$$

$$A_3 = \frac{1}{2 h_i (1 - \cos\theta_a^{app})} + \frac{\delta}{k_{HC} \sin^2\theta_a^{app}} , \tag{S30}$$

$$B_1 = \frac{A_2}{\tau A_1} \left[\frac{R_e^2 - R^2}{2} + R_{min}(R_e - R) - R_{min}^2 \ln\left(\frac{R - R_{min}}{R_e - R_{min}}\right)\right] , \tag{S31}$$

$$B_2 = \frac{A_3}{\tau A_1} \left[R_e - R - R_{min} \ln\left(\frac{R - R_{min}}{R_e - R_{min}}\right)\right] , \tag{S32}$$

$$\tau = \frac{3 R_e^2 (A_2 R_e + A_3)^2}{A_1 (11 A_2 R_e^2 - 14 R_e R_{min} + 8 A_3 R_e - 11 A_3 R_{min})} . \tag{S33}$$

The total surface steady state condensation heat flux (q") for a hydrophobic surface is obtained by incorporating the individual droplet heat transfer obtained from Equation S24, with the droplet size distributions (Eqns. S25 and S26):

$$q" = \int_{R_{min}}^{R_e} q(R) n(R) dR + \int_{R_e}^{\hat{R}} q(R) N(R) dR . \tag{S34}$$

The overall condensation heat transfer coefficient is calculated by:

$$h_c = \frac{q_{tot}}{\Delta T} , \tag{S35}$$

where ΔT is the temperature difference between the ambient vapor saturation temperature, T_{sat} and the condensing surface temperature, T_{surf} (i.e. $\Delta T = T_{sat} - T_{surf}$).

Supplementary Note 4e. Filmwise Condensation Heat Transfer Model

To model filmwise condensation of steam on the hydrophilic copper tube samples, the classical Nusselt model was used:[127, 135]

$$h_{\mathrm{c,filmwise}} = 0.729\left[\frac{g\rho_{\mathrm{l}}(\rho_{\mathrm{l}}-\rho_{\mathrm{v}}){k_{\mathrm{l}}}^{3}{h_{\mathrm{fg}}}'}{\mu_{\mathrm{l}}d_{\mathrm{OD}}\Delta T}\right]^{0.25}, \tag{S36}$$

$${h_{\mathrm{fg}}}' = h_{\mathrm{fg}} + 0.68c_{\mathrm{p,l}}\Delta T\,, \tag{S37}$$

where g is acceleration due to gravity (g = 9.81 m.s^{-2}), ρ_{v} is the vapor density, ρ_{l} is the condensate liquid density, μ_{l} is the condensate liquid dynamic viscosity, ${h_{\mathrm{fg}}}'$ is the modified latent heat of vaporization accounting for the change in specific heat of the condensate and $c_{\mathrm{p,l}}$ is the condensate liquid specific heat.

Supplementary Note 5. Long-term Condensation Durability

Supplementary Note 5a. Test Facility

The durability tests were performed in a customized vacuum compatible environmental chamber (Kurt J. Lesker). The main environmental box chamber (16 inch by 16 inch by 16 inch in size) consists of a front door to access all samples inside, 10 view ports (DN100CF, Kurt J. Lesker), and 17 feedthrough apertures for various components. The front door was sealed with a rubber gasket; all CF flanges were sealed with silver plated copper gaskets; and KF flanges were sealed with stainless steel centering rings accompanied by nitrile rubber O-rings. Resistive heating cables (AWH-101-040D, HTS/Amptek) were wrapped around the exterior of the chamber walls to prevent condensation on the inside chamber walls and view ports. The heaters were controlled by a voltage regulator (DNG-600PH-DK, Lutron). The chamber wall temperature was maintained at ≈40ºC throughout the experiment. Heating cables were insulated to prevent unnecessary condensation and limit heat losses to the ambient environment. Cooling water was supplied to the aluminum cold plates in the chamber from a large capacity chiller (Merlin M150, Thermo Fisher Scientific) via KF fluid feedthroughs (Kurt J. Lesker). To monitor the coolant water flow rate, an electromagnetic flow meter (FMG93-PVDF, Omega) with an accuracy of ±1% of the reading was integrated along the coolant inlet line.

To remove non-condensable gases prior to the experiments, a bellows valve (Ideal Vacuum) was attached to the top of the environmental chamber and connected to a rotary vane vacuum pump (KJLC-RV212, Kurt J. Lesker). An inline liquid nitrogen (LN2) cold trap (Kurt J. Lesker) was incorporated into the line from the environmental chamber to the vacuum pump via bellows tubes (Kurt J. Lesker) to remove any moisture from the pumped air from inside of chamber, which also helps produce a lower base pressure and achieve faster pump down. A secondary bellows valve

(Kurt J. Lesker) was integrated onto a tee flange between the vacuum pump and the LN2 trap to release the vacuum to ambient once the pump down process is finished. In order to monitor the pressure within the chamber, a vacuum pressure transducer (925 Micro Pirani, MKS) was installed on top of the chamber. A tertiary bellows valve was attached to the environmental chamber as a leak port between the inside of the chamber and the ambient to release the vacuum once the experiments are finished.

A secondary stainless-steel water reservoir (or degassing chamber) was installed to supply water vapor inside the environmental chamber. The water reservoir was connected to the environmental chamber via bellows tubes (Kurt J. Lesker) and two diaphragm valves (SS-DSS4, Swagelok). Bellows tubes were wrapped with tape heaters (HTWC101-010, Omega) to prevent condensation and icing during the pump down process. Five 6.35 mm (1/4 inch) diameter access tubes were welded directly on top of the degassing chamber flange and were attached with three independent diaphragm vales (SS-DSS4, Swagelok), a pressure relief valve, and a thermocouple. The first diaphragm valve was connected to the vapor supply line connecting the top of the water reservoir to bottom of the environmental chamber. The second valve was connected to the liquid condensate drainage line connecting the bottom of the water reservoir to the environmental chamber. The third value was connected the top of the water reservoir to the ambient air to establish a water filling port. The water reservoir was wrapped in a resistive heater (AWH-101-040D, HTS/Amptek) and insulated to limit heat losses. Heating power was controlled by a voltage regulator (DNG-600PH-DK, Lutron). To prevent over-pressurization of the water vessel, the water temperature was monitored using a K-type thermocouple located inside the water reservoir. A pressure relief valve (SS-RL3S4 , Swagelok) was set at 303.975 kPa (3 atm) to prevent overpressure. The mass of the water reservoir was monitored with a digital mass scale (CPWplus 75, Adam Equipment Inc.).

Four cold plates (12.0 inch by 3.5 inch, CP10G05, Lytron) forming heat sinks were installed inside the environmental chamber using aluminum frames and connected to the external chiller loop to promote condensation. The heat sinks were customized to hold up to 40 specimens (1 inch by 2 inch samples sizes). Screw threads were created on each cold plate to mount samples. The samples were mounted on the heat sinks using Polyether ether ketone (PEEK) screws and washers (Misumi), while the other sides of the cold plates were insulated with PEEK sheets (Misumi) to limit condensation outside of the specimen area. The PEEK material was chosen as an insulation material due to its low outgassing rate, low thermal conductivity, and excellent chemical and thermal resistance. The PEEK screws were chosen in order to limit potential for galvanic or electrochemical corrosion when connecting dissimilar sample materials to the aluminum cold plate.

To monitor temperatures within the system, K-type thermocouple bundles were connected to the chamber via thermocouple feedthroughs (TFT5KY00008B, Kurt J. Lesker). Inside the chamber, the temperature of the cold plates, side walls, water vapor, and collected condensate were monitored throughout the three-year experiment. The thermocouples and pressure transducer were electrically connected to a data acquisition (DAQ) system which was built on a CompactDAQ Chassis (cDAQ-9174, National Instruments). All collected data was analyzed with in-house LabVIEW code. The DAQ system includes a thermocouple module (NI-9213, National Instruments), a digital signal module (NI-9423, National Instruments), an analog signal module (NI-9209, National Instruments), and a terminal block (NI-9923, National Instruments). Finally, multiple DSLR Cameras (K-50, Pentax) were placed in line with all view ports on the chamber for visual recording of steam condensation process on samples to track coating degradation.

Supplementary Note 5b. Test Procedure

A set of strict procedures were followed to ensure consistency throughout the experiments. First, the chamber interior and sample mounting rig were thoroughly cleaned with isopropyl alcohol (IPA) to remove any contaminants. The water reservoir was filled with deionized (DI) water. The heating cables wrapping the environmental chamber were energized to maintain the chamber walls at ≈ 40ºC. The chamber walls were heated to dry out the inside of the chamber prior to pumping down and prevent condensation during the experiments. The test samples were mounted on aluminum cold plates using PEEK screws and PEEK washers. Then, the test rig with cold plates were connected to the coolant line. Simultaneously, thermocouples were attached to cold plates and chamber walls.

Given the long period of the durability test (> 1 month), a leak test was performed to ensure that no leaks were present before each experimental trial. All diaphragm valves on the degassing chamber and the leak port bellows valve on the environmental chamber were closed. The LN2 trap was filled with LN2. The bellows valve connecting the vacuum pump to the ambient was closed, and another bellows valve connecting the environmental chamber and LN2 trap was opened. Then, the vacuum pump was turned on to initiate the pump down process. The chamber pressure was monitored using the pressure transducer. In the middle of the pump down process, the large capacity chiller was turned on, and the coolant temperature was set to 10°C. The inflow rate of the coolant was monitored using the electromagnetic flowmeter. This process took approximately one hour to achieve the target vacuum conditions ($P < 5$ Pa). Whenever necessary, the LN2 trap was refilled. The LN2 trap was cleaned midway during the pump down process, since frost would form on the LN2 trap and block the vacuum line during pump down. When the target pressure was achieved, the valve connecting the environmental chamber and the LN2 trap was closed, and the

pump was turned off. Then, the valve connecting the vacuum pump and the ambient was opened to release vacuum in the pump line. The chamber was left under vacuum for >24 hours to perform the leak test, and the chamber pressure was monitored with the pressure transducer. The leak rate was characterized to acquire the fidelity of the data. The initial leak rate was controlled to less than 1 $Pa.min^{-1}$, with the long term leak rate controlled to less than 0.1 $Pa.min^{-1}$. When the leak test was finished, the vacuum was released by opening the valve connecting the environmental chamber and the ambient.

Once the leak test was finished, the experiment was initiated. The vapor supply diaphragm valve and the water filling valve on the water reservoir were opened, and the tape heater around the water reservoir was turned on with the voltage regulator set to maximum output to boil the water. During the boiling process, the excess water that spilled from the water reservoir was removed. In the meantime, most of the dissolved gas in the water was removed. The temperature of the water and reservoir were monitored using thermocouples. Once the water temperature reaches slightly higher than 100°C for at least 10 minutes, the voltage regulator was turned down, and all the valves were closed. Then, the vacuum pump down process was repeated.

When the target pressure ($P < 5$ Pa) inside the environmental chamber was reached (Supplementary Figure 6), the bellows valve connecting the environmental chamber and LN2 trap was closed, the vacuum pump was turned off, and the valve connecting the vacuum pump and the ambient was opened to release the vacuum. Immediately after closing the bellows valve on the environmental chamber, the vapor supply valve and condensate drainage valve on the water reservoir were opened. Steady state conditions were typically reached after approximately 30 min of full operation. Throughout the experiments, the data (temperatures, pressure, and mass scale) was collected, and the images of each sample were recorded using the DSLR cameras.

Supplementary Note 5c. Bond Number Calculation to Predict Filmwise to Dropwise Condensation Transition

The droplet Bond number is defined as $\mathrm{Bo} = R_\mathrm{f}^2/l_\mathrm{y}^2$, where R_f is the characteristic lateral length of the liquid droplet, taken to be its final equilibrium radius immediately before departure. The droplet departure size R_f can be calculated from a force balance between the gravitational body and contact line pinning forces and is defined as:[1]

$$R_\mathrm{f} = \sqrt{\left[\frac{6\gamma\,(\cos\theta_\mathrm{r} - \cos\theta_\mathrm{a})\sin\theta_\mathrm{e}}{\pi\rho g(2 - 3\cos\theta_\mathrm{e} + \cos^3\theta_\mathrm{e})}\right]}, \tag{S38}$$

where θ_a, and θ_r are the apparent advancing and receding contact angles, θ_e is the equivalent contact angle immediately before shedding from the condensing surface, defined as:

$$\theta_\mathrm{e} = \cos^{-1}(0.5\cos\theta_\mathrm{a} + 0.5\cos\theta_r). \tag{S39}$$

Here, l_y represents the capillary length defined as, $l_y = \sqrt{\gamma/\rho g}$, where γ and ρ are the condensate surface tension and density, respectively, and g is the gravitational constant. A previous study showed that the critical Bond number, $\mathrm{Bo}_\mathrm{crit} \approx 1.4$, forms the transition boundary, with Bo > 1.4 indicating filmwise condensation transition.[1]

Supplementary Note 6. Durability of iCVD and SAM Coatings

Durability of iCVD. We tested the durability of iCVD coatings (DVB-PFDA films) having different film thickness (30 nm and 60 nm) in the same environmental vacuum chamber system. Supplementary Figure 7 shows time-lapse images of pure steam dropwise condensation on the vertically oriented surfaces consisting of iCVD coating samples with two film thicknesses. Both surfaces showed unstable dropwise condensation (irregular shaped droplets) after 1 week of condensation.

Durability of HTMS (SAM). The self-assembled monolayer is obviously thinner than F-DLC. But it is not durable (chemically degrade) because covalent bonding with the substrate becomes chemically unstable when exposed to water.[136, 137, 138] This is why SAMs have never been demonstrated in the past to be durable dropwise condensation or hydrophobic application coatings. In our work, we tested samples consisting of Cu and Al tabs coated with SAM of heptadecafluorodecyl-trimethoxy-silane (HTMS), which failed via transition to filmwise condensation after less than one month of testing in pure steam conditions (see Table S4, Supplementary Information). To secure the adhesion between HTMS and substrate, we used the chemical vapor deposition (CVD) method to coat the surface. Specifically, we first ultrasonically cleaned the substrate using acetone for 5 min, followed by rinsing with ethanol, isopropanol (IPA), and deionized water. After drying using a clean N_2 stream, we then used air plasma (PDC-001-HP, Harrick Plasma, 30 W, 3- 5 min) to clean and activate the substrate. Air plasma cleaning has been shown to increase the number of hydroxy groups on the substrate thus to enhance the HTMS coverage[139]. The plasma-cleaned surface was then put into an atmospheric oven (Thermo Scientific, Lindberg Blue M) together with HTMS-toluene solution (5% v/v) to allow HTMS evaporation and deposition at 80-90ºC for 3 hours.

The coating methods and protocols are key to secure coating quality. One important aspect is the control of humidity during silane coating. The past studies have highlighted that elimination of water vapor during silane coating can enhance the coating quality.[140, 141] In our experiments, we plasma cleaned the sample and reduced the humidity by preheating the beaker and sample in the furnace at ~80ºC for 10-20 min before performing CVD. Although we anticipate the quality of silane coating can be further enhanced by conducting CVD in a vacuum furnace, which has been verified by others,[140, 141] our numerous experiments and calculations over the past decade of work demonstrate that these improvements alone cannot overcome the challenges of SAM hydrolysis in steam conditions, which has been well understood and characterized in the past decades.

With our F-DLC multilayer design, we eliminate pinholes, ensuring $\Omega < 4.2 \times 10^{-3}$ (see manuscript for criteria). This design is thermo-mechanically robust and exhibits excellent durability even in humid environments. Consequently, it proves to be a more attractive option compared to SAMs.

Supplementary Note 7. Droplet Departure and Heat Transfer Analyses Pre and Post 3-Year Condensation

Supplementary Note 7a. Droplet Departure Size

We performed condensation experiments focusing on the droplet departure size. Water vapor condensation was performed on both fresh and 3-year durability tested F-DLC surfaces. The duration of the tests on each sample were ~60 minutes. We randomly selected seven droplet departure phenomena for comparison. As shown in Supplementary Figure S8, droplet departure size on durability tested sample is ~28.7% larger than on fresh F-DLC coated surface This is because due to long-term exposure to humid environments, the DI water contact angle of the surface changed from θ_a/θ_r = 99.1°/86.2° to θ_a/θ_r = 70.5°/57.1°.

Supplementary Note 7a. Condensation Heat Transfer

We conducted additional condensation heat transfer experiments to compare the performance of the 3-year durability tested sample with a fresh FDLC coated sample. These experiments were carried out on flat surfaces, unlike the data presented in Figure 3 of the original manuscript, which used tube surfaces. We chose to compare heat transfer coefficients on flat surfaces because the durability test was conducted on such samples, and we only had access to flat surfaces from the 3-year durability test. We did not conduct any durability testing on tube surfaces. Flat surface condensation heat transfer experiments were conducted in a different vacuum chamber (Supplementary Figures 9a-b), which consists of a main chamber, a boiler or steam generator and a test section (Supplementary Figure 9b). The test section has a cold plate where the temperature is controlled by circulating cold water through a chiller (Supplementary Figure 9c). Additionally, another copper block in the test section had four thermocouples to monitor the temperature gradient (Supplementary Figure 9c). We attached the test sample at the end of the copper block and inserted a thermocouple into it to monitor the surface temperature.

A set of strict procedures were followed to maintain consistency across experiments and to ensure repeatable results. The test sample was connected to the copper block with thermal interface material and screws, and all connections were checked before the chamber was sealed to ensure there were no leaks. Once the environmental chamber front flange door was sealed, the vapor generator was completely filled with approximately ~5 liters of DI water and heated. After the DI water boiled (>98°C) for at least 20 minutes, the vacuum-pump-down procedure of the environmental chamber was initiated. During the vacuum-pump-down, the pressure inside the chamber was monitored using both installed pressure transducers. This process took ~45 minutes to achieve the target vacuum conditions. The environmental chamber was evacuated to a pressure

of $P < 5 \pm 0.025$ Pa with a leak rate of 0.1 Pa.min^{-1} after chamber isolation. Evacuation eliminated NCGs which add diffusional resistance to condensation heat transfer.[126]

The final step in the startup procedure was to initiate the chiller which controls the cold plate and sample temperature. A Polyscience chiller **(**6860T56A270D**)** was filled and turned on to the maximum recirculating pressure valve setting, giving a flow rate of ≈ 127 ± 2 g.s^{-1}. The temperature was set at 7 ± 1°C and the coolant was circulated through the flow loop until the test section inlet temperature (T_{in}) ranged between 7 to 8°C, and was maintained for at least 5 minutes to confirm steady state operation. In summary, the following conditions were met before injecting vapor to the environmental chamber: 1) the liquid inside the vapor generator must reach the boiling point and generate vapor for at least 20 minutes, 2) the coolant and test section temperature must be between 7°C and 8°C for at least 5 minutes, and 3) the chamber pressure must be below 1 Pa. Once these conditions were reached, the valve in the vacuum-line was closed and the vacuum pump turned off. The vent valve on the vapor generator was closed and the valve connecting the vapor generator to the environmental chamber was gradually opened to allow vapor inside the chamber until it reached a desired pressure. All thermocouples, and pressure transducer readings were recorded over a 10-minute period to obtain steady state condensation measurements.

We performed condensation hear transfer experiments on fresh and 3-year durability tested samples and compared their performance. To ensure one-dimensional (1D) heat transfer, we insulated the test section with a custom-built Teflon block. We inserted four thermocouples into the copper cylinder at known distances to provide a one-dimensional temperature gradient (dt/dx). By multiplying the temperature gradient with the thermal conductivity of the copper, we obtained the heat flux (q”). We calculated the temperature subcooling by subtracting the measured surface temperature from the saturation temperature corresponding to the saturation vapor pressure. We

calculated the condensation heat transfer coefficient by dividing the heat flux with the degree of subcooling. As shown in Supplementary Figure 9d, we observed < 30% reduction in condensation heat transfer coefficient for the 3-year durability tested sample compared to the fresh F-DLC coated copper surface. This decrease in performance is attributed to a slight increase in condensate droplet size resulting from the reduction in surface wettability after 3-year exposure to humid environments. However, we note that the condensation HTC on 3-year tested surface was ~ 74% higher than an uncoated copper sample which underwent filmwise condensation.

Supplementary Note 8. Thermo-Mechanical Stability

Supplementary Note 8a. Thermal Stability

In order to evaluate the thermal stability of FDLC under steady-state temperature conditions, we subjected the samples to a constant temperature in a controlled furnace environment. These experiments were carried out in both air and an inert atmosphere (Supplementary Figure 10). To anticipate the transition from dropwise to filmwise behavior, we determined the Bond number (Bo) by measuring the contact angle at various temperatures following exposure (Supplementary Figure 10)**.**

In real applications, the elevated temperature is not constant, but rather goes through fluctuations of high and low temperatures. Such temperature variations may result in degradation of the coating due to the mismatch of the thermal expansion coefficient between the substrate and the coating layer. To evaluate the coating's performance in such environments, we conducted a thermal cycling experiment using a laboratory test chamber (Lab event, Weisstechnik) (Supplementary Figure 11a). As depicted in Supplementary Figure 11b, for each cycle, the chamber environment is set to increase from 25°C to 175°C, which takes about an hour, followed by an idle period at 175°C for an additional hour. Then, the chamber temperature starts decreasing from 175°C to -25°C, which takes approximately 2 hours, followed by an hour of constant temperature at -25°C, and finally, the temperature increases from -25°C to 25°C, thus completing the first cycle (Supplementary Figure 11b). Each cycle requires approximately 6 hours to complete, and we conducted experiments for a total of 120 cycles, which took roughly 30 days. For this study, we chose both copper and silicon wafers coated with F-DLC. For each substrate, we placed six identical samples on a ceramic boat inside the chamber. After 20 cycles, we removed the first sample of each type. Thereafter, we took out the rest of the samples sequentially at 40, 60, 80, 100, and 120 cycles.

After each cycling experiment, we measured the apparent advancing (θ_a) and receding (θ_r) DI water droplet contact angles using a microgoniometer (MCA-3, Kyowa Interface Science). Even after 120 cycles, we did not observe any significant change in the apparent contact angle, indicating no coating degradation (Supplementary Figure 11c). Both silicon and copper coated substrates showed similar performance.

Supplementary Note 8b. Mechanical Abrasion Characterization

The abrasion resistance of the F-DLC coating on the polished silicon substrate was tested using a Taber Reciprocating abrader (Model-5900) (Supplementary Figure 12). A sliding specimen platform moves in a horizontal, reciprocating motion under a stationary abradant (Calibrase® CS-10, Taber Industries). During the test, the abrasion speed was set to 30 cycles per minute with a 1 N pre-load applied to the abradant holding arm. After each desired abrasion test cycles, abradant residuals on the F-DLC sample were removed by sonicating for 5 minutes in ethanol followed by rinsing with DI water. Then apparent advancing contact angle (θ_a) and receding contact angle (θ_r) of water on the samples were measured using micro-goniometry (MCA-3, Kyowa Interface Science Co., LTD).

Supplementary Note 8c. Fluorine Distribution Across the F-DLC Coating (Thickness Direction)

To confirm the distribution of fluorine in the multi-layer F-DLC coating, we conducted EDS line scan analysis on a cross section of coating layer. Supplementary Figure 13 shows the fluorine signal intensity along the scan line. In the Ti, DLN and DLC layers, the signal is nearly zero. However, the signal intensity in the f-DLC layer increases from bottom of that individual layer to the surface top.

Supplementary Note 9. Effect of Vapor Pressure and NCGs on Coating Condensation Lifespan

Non-condensables (NCGs) are gases that will not condense into a liquid within regular operating conditions. Even a small fraction of NCGs (below 0.1%) seriously hampers steam penetration to the target surface.[142] In condensation applications, there exists a complex vapor concentration profile due to the presence of NCGs.[143, 144] The presence of NCGs act as diffusion barrier preventing fresh steam coming in contact with the condensing surface, which results in reduced droplet nucleation density and slower condensation rates. In contrast, experimental environments devoid of NCGs and high vapor pressures surround the condensing surface with pure steam, ensuring enhanced droplet nucleation density and high condensation rates. This also provides harsher conditions for survival of the coatings.

To evaluate the behavior of the coatings in slow and rapid condensing environments, we performed a lifespan study of a hydrophobic chemistry (HTMS) deposited on polished aluminum in two different condensation chambers. For both chambers, we selected pure steam conditions having different vapor pressure and temperature. We intentionally avoided running the experiments in ambient conditions (in the presence of NCGs) for several reasons. NCGs provide a complex vapor concentration profile and diffusion barrier. Moreover, running experiments in such environments and resultant data will be dependent on types on NCGs present in the experimental environment. From a practical perspective, it makes more sense not to run the experiments in a non-standard NCG environment and wait for months for failure. Rather, we evaluate the lifespan in a harsher environment which is also reproducible.

Supplementary Figure 14 shows the durability study of HTMS coated aluminum surfaces under two different vapor pressures. The HTMS was coated following the same chemical vapor deposition (CVD) process described in the manuscript. Supplementary Figure 14a shows the

lifespan of the coating in pure vapor conditions having a vapor pressure of ~2 kPa. This test was performed in the same vacuum chamber facility as discussed in the manuscript. The results show that the coating fails after a period of ~ 30 days (Supplementary Figure 14a). To evaluate the effect of higher condensation rate (higher vapor pressure), we developed a highly accelerated life testing (HALT) chamber facility and evaluated the durability of the same HTMC coating in that chamber. The HALT chamber conducted the experiment in pure steam conditions at a vapor pressure of ~25 kPa, ensuring harsher conditions for the coating. As expected, after exposure to such highly accelerated condensing environments, the HTMS coating failed within 40 minutes (Supplementary Figure 14b). From a direct comparison, we can see that the HTMS performs ~720X longer at low condensation rate environments. Our systematic experimental demonstration clearly shows the effect of testing environment on the true value of coating lifespan. While reporting, the scientific community should clearly present the durability data along with the testing conditions.

Supplementary Note 10. F-DLC Thickness and Thermal Resistances

The rational design of the multi-layer F-DLC coating was guided by our physics-based understanding of condensation-induced blistering. The quantitative parameter that describes blistering, Ω, demonstrates that delamination of a hydrophobic coating will occur if $\Omega > 1$.[145] Specifically, the blistering parameter Ω is governed by the pinhole size, R_{d}, the base radius of the pinhole-adjunct delaminated region, R_{b0}, the liquid-vapor surface tension of the working fluid (water) γ, as well as the coating intrinsic properties including its wet adhesion G, Young's modulus E and coating thickness h:

$$\Omega = \left(\frac{1.04R_{\mathrm{b0}}}{R_{\mathrm{d}}}\right)\left(\frac{\gamma^4}{EG^3h}\right)^{\frac{1}{4}}$$

For a typical 100-nm thick fluoropolymer deposited on a smooth metal substrate ($R_{\mathrm{b0}} \approx 5R_{\mathrm{d}}, E \approx 1$ GPa, $G \approx 10\ \mathrm{mJ.m^{-2}}$), $\Omega \approx 3.6$. Hence, polymers are unable to prevent delamination, unless their thicknesses exceed 10 μm (where $\Omega \approx 1$). Our multi-layer F-DLC coating decreases Ω by using several synergistic approaches. By co-depositing short-chain PFCs (perfluorinated compounds) with the top DLC (a-C:H) surface (fDLC), we enable high Young's modulus of E = 78 GPa and low surface energy of ~ 24 $\mathrm{mJ.m^{-2}}$. By deploying a well-established titanium (Ti) bonding layer, we enable an interfacial toughness of ~ 10 $\mathrm{J.m^{-2}}$. Utilizing our Ti-DLC-fDLC multilayer, we eliminate pinholes, ensuring $\Omega < 4.2 \times 10^{-3}$ via the design of a 1-μm thick multi-layer F-DLC coating. While the Ti-DLC-fDLC multilayer focuses primarily on blistering and delamination, satisfying abrasion resistance and high temperature stability requires further stack modification. In addition to the aforementioned three-layer design, we included an additional layer composed of co-deposited DLC and silica (a-C:H:Si:O, amorphous hydrogenated carbon films containing silicon and oxygen) between the DLC layer and the Ti adhesion layer, which we term

DLN. The added DLN layer is well-adapted in conventional DLC multilayers to: 1) enhance adhesion with the Ti layer by silica infusion, 2) provide good thermal stability and act as a stress reliever, and 3) further decrease the pinhole density.

As we discussed above and, in the manuscript, our multi-layer architecture has several functionalities such as: strong interfacial toughness, thermal buffering, mechanical robustness, low surface energy, and pinhole prevention. The current design and thickness are optimized considering all these factors. Reducing the thickness or layer numbers with the existing recipe is not possible, because it will force to sacrifice the durability/robustness.

Furthermore, if we calculate the thermal resistance of the coating at the current design thickness, and then compare it to the thinner coating resistance, we see that both values are substantially smaller than the coolant side thermal resistance, the wall resistance, or the condensation heat transfer coefficient resistance, implying that using a thinner coating is not necessary to enhance performance (Supplementary Table 8). We agree that had the coating been 20 μm thick, the resistance would have been larger and more dominant (Supplementary Table 8).

The overall heat transfer performance from the vapor to the cooling water can be expressed as a series sum of four individual thermal resistances: the internal convective resistance $R_{\text{convection}}$, the conduction copper tube wall resistance R_{wall}, the conductive resistance imposed by the coating R_{coating}, and the condensation heat transfer resistance $R_{\text{condensation}}$, as shown in Supplementary Figure 4.

Considering a copper tube sample having outer diameter D_{OD} = 9.53 mm, inner diameter D_{ID} = 8.0 mm, and length L = 134.6 mm. And F-DLC coating with thickness h = 1.65μm and thermal conductivity $\mathrm{k}_{\mathrm{F-DLC}} \sim 0.47$ W.m^{-1}.K^{-1}, the thermal resistance of the coating sample is:

$$R_{\mathrm{coating}} = \frac{\ln\left(\frac{d_{\mathrm{OD}} + h}{d_{\mathrm{OD}}}\right)}{2\pi L k_{\mathrm{FDLC}}} \approx 4.36 \times 10^{-4}\ \mathrm{K.W^{-1}}$$

Similarly, the conductive tube wall resistance can be calculated by knowing the thermal conductivity of the wall ($k_{\mathrm{Cu}} = 386$ W.m^{-1}.K^{-1}, and using:

$$R_{\mathrm{wall}} = \frac{\ln\left(\frac{d_{\mathrm{OD}}}{d_{\mathrm{ID}}}\right)}{2\pi L k_{\mathrm{Cu}}} \approx 5.36 \times 10^{-4}\ \mathrm{K.W^{-1}}\ .$$

Compared to these two resistances, for a standard experimental conditions ($\mathrm{h_i} \approx 25$ kW.m^{-2}.K^{-1}; h ≈ 10 kW.m^{-2}.K^{-1}) the internal convective resistance and condensation heat transfer resistance are significantly larger.[124, 125]

$$R_{\mathrm{convection}} = \frac{1}{h_{\mathrm{i}} \pi d_{\mathrm{ID}} L} \approx 0.0118\ \mathrm{K.W^{-1}}\ ,$$

$$R_{\mathrm{condensation}} = \frac{1}{h \pi d_{\mathrm{OD}} L} \approx 0.024811\ \mathrm{K.W^{-1}}\ ,$$

Thus, both the tube wall and the additional coating thermal resistances are significantly lower than condensation and internal convection resistances.

Supplementary Note 11. Economic Assessment of Coating Methods

Economic Assessment: Our economic assessment involves a comparison between the simple chemical vapor deposition method used for HTMS coating and our F-DLC coating.

HTMS (SAM): For the purposes of our analysis, we are considering only the energy and chemical costs associated with it. The capital cost of equipment and labor costs are not being taken into account.

(Heptadecafluoro-1,1,2,2-Tetrahydrodecyl)Trimethoxysilane based coating is used for generating hydrophobic coating, this is chemical vapor deposition (CVD) process. To fabricate the hydrophobic surface, the Al and Cu tabs were functionalized with heptadecafluorodecyltrimethoxy-silane (HTMS, TCI America, CAS #: 83048-65-1) using the vapor phase deposition method.[146] Briefly, the substrates were placed in a glass beaker with a vial of HTMS toluene solution (5% v/v). A glass lid was placed on top to seal the container, followed by heating in atmospheric pressure oven (Thermo Scientific, Lindberg Blue M) at 80± 5°C for 3h to allow conformal HTMS SAM deposition.

Chemical Cost. The price of 25ml HTMS coating solution is $278.[147] Based on our experience, 1ml of solution can effectively coat an area of ~0.2m^2. Therefore, using 25ml of solution we can coat ~5m^2 surface area. As a result, the estimated cost of applying HTMS per unit area of surface is ~$278/5 m^2 ~ $55.60/m^2.

The cost of 1litre of toluene is ~$79 (Sigma Aldrich). For the 25ml HTMS solution, we will need 475ml of toluene at a 5% : 95% volume ratio. [146] Therefore, the cost of toluene for coating 5m^2 of surface area is ~$(79×475ml/1000ml)/5m^2 ~$7.5/m^2.

Energy cost. The power consumption of the furnace for 3 hours is approximately 3h × 3500W = 10kWh. The average price of electricity in Illinois is 12.95¢ per kWh (according to the Illinois Energy Rating in 2023). Therefore, the total energy cost for one batch of HTMS coating is around $1.4. The interior dimensions of the furnace are 22.9 × 22.9 × 35.6 cm (Thermo Scientific, Lindberg Blue M), giving an interior surface area of ~ 1.45m^2 square meters. Thus, the overall energy cost per unit area is approximately $1.4/1.45 m^2, which equals approximately $0.97/m^2.

Therefore, the overall cost of applying HTMS coating using a CVD process for 1m^2 of surface area is approximately ($55.60/ m^2 + $7.5/ m^2+ $0.97/ m^2) = $64.07/ m^2.

Because coating systems vary in their lifetimes, applied cost per year should be used to denote the true cost of a coating system. The proper method of developing costs for coating systems is to use life cycle costs (LCCs); that is, the applied cost of the entire coating system per year of coating life.[148] The service lives of coating systems are dependent on material and environmental exposure conditions. Service-life estimates are based on experience or projections from accelerated scientific investigations that truly mimic the failure mode relative to the specific exposure conditions.[148]

Based on our accelerated condensation environmental test, the estimated service life of the HTMS-based coating is approximately 29 days (0.0795 year) (Supplementary Table 4), Therefore, the total applied coating system cost for HTMS is ~$64.07 /m^2/ 0.0795 year ~ $805.91/ m^2/year.

FDLC: The four-layer F-DLC coating manufacturing process utilizes a combination of PVD sputtering and PACVD, which are both performed in the same cylindrical chamber with an interior dimension of 25" (H) x 12" (D). Based on the manufacturer's information (Oerlikon Balzers), the estimated surface area that can be coated per batch of F-DLC coating is approximately 0.96 m^2. The cost of coating one batch of the sample, including the coating material cost, energy cost, and labor cost, is approximately $4000.

Therefore, the costing cost of F-DLC for 1m^2 of surface area is approximately $4000/0.96 m^2 ~ $4166.67/$m^2$. According to our condensation durability test, the estimated service life of the F-DLC coating for condenser application is greater than 3 years (Supplementary Table 4). Therefore, the total applied coating system cost for F-DLC is ~$4166.67/$m^2$/ 3 year ~ $1388.89/ m^2/year.

Payback Period:

To determine the payback period for implementing F-DLC in a power plant condenser, let's consider a typical 500MW power plant with an annual generation capacity of 3.5 TWh.$year^{-1}$. [149] Assuming a durable condenser tube leads to a 2% improvement in efficiency, resulting in an overall 2% increase in yearly electricity production, which is equivalent to 0.07 TWh.$year^{-1}$. For the calculation, we'll exclude the additional monetary benefit of CO2 emissions reduction. Based on the average price of electricity in the USA, which is 16.5¢ per kWh according to the US Bureau of Labor Statistics, the 0.07 TWh.$year^{-1}$ of electrical power generation would amount to $1.15 × 10^7/year ($11.5 million/ year).

The effective surface area to be coated on a typical 50 MW power plant condenser is ~750 m^2.[150] Based on the previous cost analysis, the total applied coating system cost for F-DLC is ~$4000/$m^2$.

Therefore, the total cost of coating on a 500 MW power plant condenser, which is assumed to have 10 times the surface area of a 50 MW plant, would be approximately (750×10) m^2×\$4000 ~ \$$0.3\times10^8$.

To determine the payback period, we divide the total cost of coating by the annual benefit from enhanced electrical power generation. Thus, the payback period is approximately ~ \$ 0.3×10^8 / \$$1.15\times10^7$/year ~ 2.6 years (excluding the cost of CO_2 emissions reduction).

Please note that these calculations consider the provided information and assumptions made. Actual scenarios may involve additional factors and considerations that could impact the real costs and payback period. It's important to note that the coating cost mentioned earlier is specific to your existing facility. However, when scaling up to a power plant level, it is reasonable to anticipate a significant decrease in costs. Typically, economies of scale come into play, resulting in reduced costs as the size of the operation increases. Therefore, when implementing F-DLC at a power plant level, it is possible that the cost could decrease by a factor of 10 or more compared to the cost at your current facility. This suggests that the payback period for the investment in F-DLC may be even more favorable when considering the cost reduction during scale-up.

Compared to HTMS coating, the applied coating system cost of F-DLC is higher. It is important to consider the long-term benefits of using F-DLC coating, such as its significantly longer service life compared to HTMS coating. The F-DLC system used in this study is performed in a small chamber, in contrast to the widespread use of large and inexpensive PVD systems worldwide. However, since F-DLC is a combination of PVD and PACVD processes and utilizes the same

facility, scaling up the system for larger production can reduce costs to be comparable to those of PVD systems.

Global high technology markets such as consumer electronics (computer and display) and renewable energy (solar) have driven thin film technology adoption at a rapid scale over the last 40 years. The technology required for manufacturing individual components such as integrated circuits, hard disks, touch panel displays, solar cells and other components relies increasingly on thin film equipment. Thin film technology has become more complex over the years, while costs have continually been driven downward as adoption is driven to a large scale. A good example of high-volume manufacturing thin film technology is in the hard disk industry where ~1.4 billion disks are manufactured on sputtering systems (PVD) annually. The technology required for hard disks involves sputtering complex stacks of multiple metals (magnetic and non-magnetic) in layers as thin as 1nm and as thick as 200nm to form the nanostructure magnetic grains used for hard disk storage, additionally thin film layers of diamond like carbon (DLC) are deposited as final steps and used as a protective anti head-disk crash layer. All of these thin films layers are deposited in a single sputtering system with a throughput of 1000 disks per hour and a cost of less than 30 cents per part.[151] Moreover, the equipment used in PVD technique requires low maintenance and the process is environmentally friendly. The coating process does not create any hazardous waste compared to other types of coating, such as electroplating or painting.[152] Moreover, PVD coated products last longer, which reduces the risk of solid waste generation and indirectly helps keep the environment clean. The vacuum environment in the deposition chamber will reduce the gaseous contamination in the deposition process to a very low level. [153]

The global physical vapor deposition (PVD) market size was estimated at USD 23.5 billion in 2021 and it is expected to surpass around USD 47.7 billion by 2030 with a registered compound annual growth rate **(**CAGR) of 8.18% during the forecast period 2022 to 2030**.**[2] PVD has been widely adopted in large-scale applications such as solar products, which contribute significantly to the overall market share (Supplementary Figure 15). The increasing adoption of solar energy as a clean energy source propels the demand for solar products. Thus, increasing usage of solar panels and solar cells paves the way for the growth of PVD.[154] The direct method of harnessing solar energy is the solar thermal conversion method using solar absorbers. The absorbers are coated with solar selective coatings often performed with physical vapor deposition (PVD) methods and are used in concentrating solar power (CSP) systems for solar thermal power generation.[155] According to the International Energy Agency (IEA), CSP systems are becoming a crucial technology for mitigating climate change. The IEA report states that by 2050, CSP systems could provide 11.3% of global electricity, with 9.6% from solar power and 1.7% from backup fuels (i.e., fossil fuels and biomass) .[156] At present, parabolic trough technology is the most established and cost-effective large-scale solar power technology for solar thermal power generation. These systems currently have an installed capacity of 870 MW, with 2152 MW under construction and 10 GW in development. As of 2020, more than 4,000 MWe of the worldwide installed operating CSP capacity utilize parabolic trough collectors. [157] The estimated cost of solar collector installation ranges from $\$90/m^2$-$\$140/m^2$, [158] which includes manufacturing, purchased components, and installation costs.

CVD is another method of deposition under vacuum and is the process of chemically reacting to a volatile compound from a material to be deposited with other gases, in order to produce a non-volatile solid that is deposited onto a substrate. This method is sometimes used as pre-coating with

the aim of increasing the durability of the substrates, decreasing the friction, and improving the thermal properties—this means that one can combine deposition methods, like layers of PVD and CVD (Supplementary Table 9), in the same system (similar to our F-DLC deposition method). [113, 159]

The widespread adoption of PVD technology can significantly reduce costs and carbon footprint, driving demand for PVD equipment in surface coating companies worldwide.[2] Our F-DLC deposition process uses a combination of PVD and PACVD methods in a vacuum system. This implies that the scalability and cost reduction opportunities demonstrated in existing PVD markets, particularly solar collectors, apply to our F-DLC deposition methods. Like PVD, our process is environmentally friendly as it operates within a controlled chamber and generates no solid waste. However, further research could investigate the use of other chemistries, such as Si, Si+O, or other dopants, to create different types of DLC coatings. This could result in the development of a range of new and improved DLC coatings for various energy applications.

The utilization of scalable deposition methods can be crucial in reducing production costs and enhancing material efficiency, which is essential for sustainable manufacturing processes. However, it may be questioned whether Dip or Spray coating methods can be used for depositing F-DLC. Unfortunately, our current recipe cannot be applied using Dip or Spray coating. While Dip and Spray coating are generally scalable methods, they present challenges in various ways. Dip coating is a deposition technique where a substrate is immersed in a liquid solution or suspension of a material and gradually withdrawn at a controlled pace before being thermally treated to cure the coating. This method is scalable, making it ideal for large-scale production. However, Dip coatings involve solvents that emit volatile organic compounds (VOCs) and per- and polyfluoroalkyl substances (PFAS), [160]. PFAS are a class of chemicals that don't naturally

break down, and so they accumulate in water, soil, and in the human body. Studies have shown that high levels increase the risk of cancer and other adverse health effects.[161] The removal of PFASs from surface water, groundwater, soil, sediment and biota is technically extremely difficult and very costly, if at all possible. Considering the effects of PFAs on human health and environment, on February 2023, European Chemical Agency (ECHA) banned around 10000 PFASs. On the other hand, spray coating is another scalable process that can be used on a wide range of materials, from ceramics to metallics. A solution of nanocompounds, adhesives, matrix material, and solvent is atomized by pressure and directed toward the substrate. However, the performance and properties of a coating depend on several parameters, including particle velocity, size, temperature, and position, substrate roughness and chemistry. Spray coatings are directional[162] and difficult to control thickness accurately,[163] and the bind mechanism may not be compatible with complex substrates. Furthermore, achieving a uniform dip or spray coating on a multi-tube condenser poses significant challenges and is almost impossible to accomplish.

Supplementary References

1. Cha H, *et al.* Dropwise condensation on solid hydrophilic surfaces. *Sci Adv* **6**, eaax0746 (2020).

2. Research P. Physical Vapor Deposition Market. In: *Chemical and Material*) (2022).

3. Molian PA, Janvrin B, Molian AM. Laser Chemical-Vapor Deposition of Fluorinated Diamond Thin-Films for Solid Lubrication. *Wear* **165**, 133-140 (1993).

4. d'Agostino R, Lamendola R, Favia P, Giquel A. Fluorinated diamondlike carbon films deposited from radio-frequency glow discharge in a triode reactor. *Journal of Vacuum Science & Technology A: Vacuum, Surfaces, and Films* **12**, 308-313 (1994).

5. C. Vivensang and G. Turban EAaAG. Reactive ion etching of diamond and diamond-like carbon films. *Diamond and Related Materials* **3**, 645-649 (1994).

6. K. TROJANM G, n d H. DIMIGEN. Network Modification of DLC Coatings to Adjust a Defined Surface. *phys stat sol (a)* **145**, 575 (1994).

7. Grischke M, Bewilogua K, Trojan K, Dimigen H. Application-oriented modifications of deposition processes for diamond-like-carbon-based coatings. *Surf Coat Tech* **74-75**, 739-745 (1995).

8. Butter RS, Waterman DR, Lettington AH, Ramos RT, Fordham EJ. Production and wetting properties of fluorinated diamond-like carbon coatings. *Thin Solid Films* **311**, 107-113 (1997).

9. C.Donnet JF, A.Grill,V.Patel,C.Jahnes,M. Belin. Wear-resistant fluorinated diamondlike carbon films. *Surface and Coatiing Technology* **94-95**, 531-536 (1997).

10. G. KOCH DCZ, A. LEIPERTZ, M. GRISCHKE, K. TROJAN and H. DIMIGEN. Sturdy on plasma enhanced CVD coated matetrial to promote dropwise condensation of steam. *Inl J Heaf Moss Transfer* **41**, 1899-1906 (1998).

11. Cerd Koch KK, Alfred Leipertz. Parameter study on the performance of dropwise condensation. *Rev Gin Therm* **37**, 539-548 (1998).

12. Miyoshi K. Lubrication by diamond and diamondlike carbon coatings. *Journal of Tribology-Transactions of the Asme* **120**, 379-384 (1998).

13. Grill A, Patel V, Jahnes C. Novel low k dielectrics based on diamondlike carbon materials. *Journal of the Electrochemical Society* **145**, 1649-1653 (1998).

14. Hatada R, Baba K. Preparation of hydrophobic diamond like carbon films by plasma source ion implantation. *Nuclear Instruments & Methods in Physics Research Section B-Beam Interactions with Materials and Atoms* **148**, 655-658 (1999).

15. Hakovirta M, He XM, Nastasi M. Optical properties of fluorinated diamond-like carbon films produced by pulsed glow discharge plasma immersion ion processing. *Journal of Applied Physics* **88**, 1456-1459 (2000).

16. Hakovirta M, Lee DH, He XM, Nastasi M. Synthesis of fluorinated diamond-like carbon films by the plasma immersion ion processing technique. *Journal of Vacuum Science & Technology A: Vacuum, Surfaces, and Films* **19**, 782-784 (2001).

17. Gilmore R, Hauert R. Control of the tribological moisture sensitivity of diamond-like carbon films by alloying with F, Ti or Si. *Thin Solid Films* **398**, 199-204 (2001).

18. Hakovirta M, Verda R, He XM, Nastasi M. Heat resistance of fluorinated diamond-like carbon films. *Diamond and Related Materials* **10**, 1486-1490 (2001).

19. Ji H*, et al.* Hydrophobic fluorinated carbon coatings on silicate glaze and aluminum. *Thin Solid Films* **405**, 104-108 (2002).

20. Veerasamy VS. Hydrophobic Coating Including DLC on Substarte. USA (2002).

21. Bottani CE, Lamperti A, Nobili L, Ossi PM. Structure and mechanical properties of PACVD fluorinated amorphous carbon films. *Thin Solid Films* **433**, 149-154 (2003).

22. Yu GQ, Tay BK, Sun Z, Pan LK. Properties of fluorinated amorphous diamond like carbon films by PECVD. *Applied Surface Science* **219**, 228-237 (2003).

23. Trippe SC, Mansano RD, Costa FM, Silva RF. Mechanical properties evaluation of fluor-doped diamond-like carbon coatings by nanoindentation. *Thin Solid Films* **446**, 85-90 (2004).

24. Yao ZQ, Yang P, Huang N, Sun H, Wang J. Structural, mechanical and hydrophobic properties of fluorine-doped diamond-like carbon films synthesized by plasma immersion ion implantation and deposition (PIII–D). *Applied Surface Science* **230**, 172-178 (2004).

25. Nakamura T, Ohana T, Suzuki M, Ishihara M, Tanaka A, Koga Y. Surface modification of diamond-like carbon films with perfluorooctyl functionalities and their surface properties. *Surface Science* **580**, 101-106 (2005).

26. Oh T, Choi CK, Lee K-M. Investigation of a-C:F films as hydrogenated diamond-like carbon and low-k materials. *Thin Solid Films* **475**, 109-112 (2005).

27. Zhao Q, Wang X. Heat transfer surfaces coated with fluorinated diamond-like carbon films to minimize scale formation. *Surface and Coatings Technology* **192**, 77-80 (2005).

28. Yu GQ, Tay BK, Sun Z. Fluorinated amorphous diamond-like carbon films deposited by plasma-enhanced chemical vapor deposition. *Surface & Coatings Technology* **191**, 236-241 (2005).

29. Saito T, *et al.* Antithrombogenicity of fluorinated diamond-like carbon films. *Diamond and Related Materials* **14**, 1116-1119 (2005).

30. Ahn HJ, *et al.* Ion-Beam Induced Liquid Crystal Alignment on Diamond-like Carbon and Fluorinated Diamond-like Carbon Thin Films. *Japanese Journal of Applied Physics* **44**, 4092-4097 (2005).

31. Jiang M, Ning Z. Influence of deposition pressure on the structure and properties of fluorinated diamond-like carbon films prepared by RF reactive magnetron sputtering. *Surface and Coatings Technology* **200**, 3682-3686 (2006).

32. Hasebe T, *et al.* Lubrication performance of diamond-like carbon and fluorinated diamond-like carbon coatings for intravascular guidewires. *Diamond and Related Materials* **15**, 129-132 (2006).

33. Yamada N, Kato Y, Kanda K, Haruyama Y, Matsui S. Surface Modification of Diamond-like Carbon by Synchrotron Radiation Exposure under the Perfluorohexane Gas Atmosphere. *Japanese Journal of Applied Physics* **45**, 6400-6404 (2006).

34. Hasebe T, *et al.* Depth profiling of fluorine-doped diamond-like carbon (F-DLC) film: Localized fluorine in the top-most thin layer can enhance the non-thrombogenic properties of F-DLC. *Thin Solid Films* **516**, 299-303 (2007).

35. Hasebe T, *et al.* Effects of surface roughness on anti-thrombogenicity of diamond-like carbon films. *Diamond and Related Materials* **16**, 1343-1348 (2007).

36. Ahmed SF, Mitra MK, Chattopadhyay KK. The effect of fluorine doping and temperature on the field emission from diamond-like carbon films. *Journal of Physics: Condensed Matter* **19**, (2007).

37. Hasebe T, *et al.* Fluorine doping into diamond-like carbon coatings inhibits protein adsorption and platelet activation. *J Biomed Mater Res A* **83**, 1192-1199 (2007).

38. Yamada N, Nakamatsu K-i, Kanda K, Haruyama Y, Matsui S. Surface Evaluation of Fluorinated Diamond-Like Carbon Thin Film as an Antisticking Layer of Nanoimprint Mold. *Japanese Journal of Applied Physics* **46**, 6373-6374 (2007).

39. Schvartzman M, Mathur A, Hone J, Jahnes C, Wind SJ. Plasma fluorination of carbon-based materials for imprint and molding lithographic applications. *Appl Phys Lett* **93**, 153105 (2008).

40. Schvartzman M, Mathur A, Kang Y, Jahnes C, Hone J, Wind SJ. Fluorinated diamondlike carbon templates for high resolution nanoimprint lithography. *Journal of Vacuum Science & Technology B: Microelectronics and Nanometer Structures* **26**, 2394-2398 (2008).

41. Chen G, Zhang J, Yang S. Fabrication of hydrophobic fluorinated amorphous carbon thin films by an electrochemical route. *Electrochemistry Communications* **10**, 7-11 (2008).

42. Sui JH, Zhang ZG, Cai W. Surface characteristics and electrochemical corrosion behavior of fluorinated diamond-like carbon (F-DLC) films on the NiTi alloys. *Nuclear Instruments and Methods in Physics Research Section B: Beam Interactions with Materials and Atoms* **267**, 2475-2479 (2009).

43. Bendavid A, Martin PJ, Randeniya L, Amin MS. The properties of fluorine containing diamond-like carbon films prepared by plasma-enhanced chemical vapour deposition. *Diamond and Related Materials* **18**, 66-71 (2009).

44. Rubio-Roy M, *et al*. Effects of environmental conditions on fluorinated diamond-like carbon tribology. *Diamond and Related Materials* **18**, 923-926 (2009).

45. Schvartzman M, Wind SJ. Plasma fluorination of diamond-like carbon surfaces: mechanism and application to nanoimprint lithography. *Nanotechnology* **20**, 145306 (2009).

46. Ahmed SF, Banerjee D, Chattopadhyay KK. The influence of fluorine doping on the optical properties of diamond-like carbon thin films. *Vacuum* **84**, 837-842 (2010).

47. Su XJ, Zhao Q, Wang S, Bendavid A. Modification of diamond-like carbon coatings with fluorine to reduce biofouling adhesion. *Surface and Coatings Technology* **204**, 2454-2458 (2010).

48. Marciano FR, Lima-Oliveira DA, Da-Silva NS, Corat EJ, Trava-Airoldi VJ. Antibacterial activity of fluorinated diamond-like carbon films produced by PECVD. *Surface and Coatings Technology* **204**, 2986-2990 (2010).

49. Bendavid A, Martin PJ, Randeniya L, Amin MS, Rohanizadeh R. The properties of fluorine-containing diamond-like carbon films prepared by pulsed DC plasma-activated chemical vapour deposition. *Diamond and Related Materials* **19**, 1466-1471 (2010).

50. Kanda K, *et al*. Fabrication of fluorine-terminated diamond-like carbon thin film using a hyperthermal atomic fluorine beam. *Diamond and Related Materials* **20**, 703-706 (2011).

51. Ahmed MH, Byrne JA, McLaughlin J. Evaluation of glycine adsorption on diamond like carbon (DLC) and fluorinated DLC deposited by plasma-enhanced chemical vapour deposition (PECVD). *Surface and Coatings Technology* **209**, 8-14 (2012).

52. Jiang A, Xiao J, Li X, Wang Z. Effect of Structure, Composition, and Micromorphology on the Hydrophobic Property of F-DLC Film. *Journal of Nanomaterials* **2013**, 1-7 (2013).

53. Jongwannasiri C, Moolsradoo N, Khantachawana A, Kaewtatip P, Watanabe S. The Comparison of Biocompatibility Properties between Ti Alloys and Fluorinated Diamond-Like Carbon Films. *Advances in Materials Science and Engineering* **2012**, 1-8 (2012).

54. Wang J, Zhang K, Zhang L, Wang F, Zhang J, Zheng W. Influence of structure evolution on tribological properties of fluorine-containing diamond-like carbon films: From fullerene-like to amorphous structures. *Applied Surface Science* **457**, 388-395 (2018).

55. Nobili L, Guglielmini A. Thermal stability and mechanical properties of fluorinated diamond-like carbon coatings. *Surface and Coatings Technology* **219**, 144-150 (2013).

56. Hasebe T, *et al.* Hydrophobicity and non-thrombogenicity of nanoscale dual rough surface coated with fluorine-incorporated diamond-like carbon films: Biomimetic surface for blood-contacting medical devices. *Diamond and Related Materials* **38**, 14-18 (2013).

57. Prihandana GS, *et al.* Antithrombogenicity of Fluorinated Diamond-Like Carbon Films Coated Nano Porous Polyethersulfone (PES) Membrane. *Materials (Basel)* **6**, 4309-4323 (2013).

58. Qiang L, Zhang B, Gao K, Gong Z, Zhang J. Hydrophobic, mechanical, and tribological properties of fluorine incorporated hydrogenated fullerene-like carbon films. *Friction* **1**, 350-358 (2013).

59. Bai S, *et al.* Tight-binding quantum chemical molecular dynamics simulations of the low friction mechanism of fluorine-terminated diamond-like carbon films. *RSC Advances* **4**, (2014).

60. Akaike S, *et al.* Relationship between static friction and surface wettability of orthodontic brackets coated with diamond-like carbon (DLC), fluorine- or silicone-doped DLC coatings. *Diamond and Related Materials* **61**, 109-114 (2016).

61. Hosseini SI, *et al.* Antibacterial properties of fluorinated diamond-like carbon films deposited by direct and remote plasma. *Materials Letters* **188**, 84-87 (2017).

62. Zhang R, Zhao J, Yang Y. A novel diamond-like carbon film. *Surfaces and Interfaces* **7**, 1-5 (2017).

63. Liangliang Liu WT, Qingdong Ruan, Zhongcan Wub, Chao Yang, Suihan Cui,, Zhengyong Ma RKYF, Xiubo Tian, Ruijun Wang, Zhongzhen Wub,, Chu PK. Robust and durable surperhydrophobic F-DLC coating for anti-icing in aircrafts engineering. *Surface & Coatings Technology* **404**, 126468 (2020).

64. Ryu H, *et al.* Enhancement of a heat transfer performance on the Al6061 surface using microstructures and fluorine-doped diamond-like carbon (F-DLC) coating. *International Journal of Heat and Mass Transfer* **148**, (2020).

65. Ma J, *et al.* Ultra-thin self-healing vitrimer coatings for durable hydrophobicity. *Nat Commun* **12**, 5210 (2021).

66. Wang D, *et al.* Design of robust superhydrophobic surfaces. *Nature* **582**, 55-59 (2020).

67. Peng CY, Chen ZY, Tiwari MK. All-organic superhydrophobic coatings with mechanochemical robustness and liquid impalement resistance. *Nature Materials* **17**, 355-+ (2018).

68. Deng X, Mammen L, Butt HJ, Vollmer D. Candle Soot as a Template for a Transparent Robust Superamphiphobic Coating. *Science* **335**, 67-70 (2012).

69. Qahtan TF, Gondal MA, Alade IO, Dastageer MA. Fabrication of Water Jet Resistant and Thermally Stable Superhydrophobic Surfaces by Spray Coating of Candle Soot Dispersion. *Sci Rep* **7**, 7531 (2017).

70. Ma J, *et al.* A Lipid-Inspired Highly Adhesive Interface for Durable Superhydrophobicity in Wet Environments and Stable Jumping Droplet Condensation. *ACS Nano* **In press**, (2022).

71. Ma J, *et al.* A Lipid-Inspired Highly Adhesive Interface for Durable Superhydrophobicity in Wet Environments and Stable Jumping Droplet Condensation. *ACS Nano* **16**, 4251-4262 (2022).

72. K.M.Holden ASW, P. J. Marto, D. H. Boone, J. W. Rose. The Use of Organic Coatings to Promote Dropwise Condensation of Steam. *Journal of Heat Transfer* **109**, 768-774 (1987).

73. Rausch M, Fröba A, Leipertz A. Dropwise condensation heat transfer on ion implanted aluminum surfaces. *International Journal of heat and Mass transfer* **51**, 1061-1070 (2008).

74. Kim K, Lee Y, Jeong JH. Dropwise condensation induced on chromium ion implanted aluminum surface. *Nuclear Engineering and Technology* **51**, 84-94 (2019).

75. Haraguchi T, Shimada R, Kumagai S, Takeyama T. The effect of polyvinylidene chloride coating thickness on promotion of dropwise steam condensation. *International journal of heat and mass transfer* **34**, 3047-3054 (1991).

76. Chang W, *et al.* Few-layer graphene on nickel enabled sustainable dropwise condensation. *Science Bulletin* **66**, 1877-1884 (2021).

77. Bonner III RW. Dropwise condensation life testing of self assembled monolayers. In: *International Heat Transfer Conference*) (2010).

78. Hoque MJ*, et al.* Life Span of Slippery Lubricant Infused Surfaces. *ACS Appl Mater Interfaces* **14**, 4598-4611 (2022).

79. Marto P, Looney D, Rose J, Wanniarachchi A. Evaluation of organic coatings for the promotion of dropwise condensation of steam. *International journal of heat and mass transfer* **29**, 1109-1117 (1986).

80. Holden K, Wanniarachchi A, Marto P, Boone D, Rose J. The use of organic coatings to promote dropwise condensation of steam. (1987).

81. Tanner D, Pope D, Potter C, West D. The promotion of dropwise condensation by monolayers of radioactive fatty acids II. Chromium surfaces. *Journal of Applied Chemistry* **14**, 439-444 (1964).

82. O'neill GA, Westwater J. Dropwise condensation of steam on electroplated silver surfaces. *International journal of heat and mass transfer* **27**, 1539-1549 (1984).

83. Finnicum SS, Westwater J. Dropwise vs filmwise condensation of steam on chromium. *International journal of heat and mass transfer* **32**, 1541-1549 (1989).

84. Yang Q, Gu A. Dropwise condensation on SAM and electroless composite coating surfaces. *Journal of chemical engineering of Japan* **39**, 826-830 (2006).

85. Kananeh AB, Rausch MH, Leipertz A, Fröba AP. Dropwise condensation heat transfer on plasma-ion-implanted small horizontal tube bundles. *Heat transfer engineering* **31**, 821-828 (2010).

86. Parin R, Rigon M, Bortolin S, Martucci A, Del Col D. Optimization of Hybrid Sol-Gel Coating for Dropwise Condensation of Pure Steam. *Materials* **13**, 878 (2020).

87. Tripathy A*, et al.* Ultrathin Lubricant-Infused Vertical Graphene Nanoscaffolds for High-Performance Dropwise Condensation. *ACS nano* **15**, 14305-14315 (2021).

88. Torresin D, Tiwari MK, Del Col D, Poulikakos D. Flow condensation on copper-based nanotextured superhydrophobic surfaces. *Langmuir* **29**, 840-848 (2013).

89. Guo L, Tang G. Dropwise condensation on bioinspired hydrophilic-slippery surface. *RSC advances* **8**, 39341-39351 (2018).

90. Xie J, Xu J, Li X, Liu H. Dropwise condensation on superhydrophobic nanostructure surface, Part I: Long-term operation and nanostructure failure. *International Journal of Heat and Mass Transfer* **129**, 86-95 (2019).

91. Wilke KL, Preston DJ, Lu Z, Wang EN. Toward condensation-resistant omniphobic surfaces. *ACS nano* **12**, 11013-11021 (2018).

92. Seo D*, et al.* Enhancing heat transfer performance of a two-phase closed thermosyphon using a polymer-coated hydrophobic condenser. *Applied Thermal Engineering* **196**, 117350 (2021).

93. Ma X, Chen J, Xu D, Lin J, Ren C, Long Z. Influence of processing conditions of polymer film on dropwise condensation heat transfer. *International Journal of Heat and Mass Transfer* **45**, 3405-3411 (2002).

94. Ma XH, Wang BX, Xu DQ, Lin JF. Lifetime test of dropwise condensation on polymer-coated surfaces. *Heat Transfer—Asian Research: Co-sponsored by the Society of Chemical Engineers of Japan and the Heat Transfer Division of ASME* **28**, 551-558 (1999).

95. Khan S. Hydrophobicity of Rare-earth Oxide Ceramics and their Application in Promoting Sustained Dropwise Condensation and Corrosion and Fouling Mitigation in Hydropower Systems.). Hydropower Foundation (2015).

96. Blackman L, Dewar M, Hampson H. An investigation of compounds promoting the dropwise condensation of steam. *Journal of Applied Chemistry* **7**, 160-171 (1957).

97. Erb R, Thelen E. Dropwise condensation characteristics of permanent hydrophobic systems. (1966).

98. Zhang BJ, Kuok C, Kim KJ, Hwang T, Yoon H. Dropwise steam condensation on various hydrophobic surfaces: Polyphenylene sulfide (PPS), polytetrafluoroethylene (PTFE), and self-assembled micro/nano silver (SAMS). *International Journal of Heat and Mass Transfer* **89**, 353-358 (2015).

99. Chandekar A, Sengupta SK, Whitten JE. Thermal stability of thiol and silane monolayers: A comparative study. *Applied Surface Science* **256**, 2742-2749 (2010).

100. Milionis A, Languasco J, Loth E, Bayer I. Analysis of wear abrasion resistance of superhydrophobic acrylonitrile butadiene styrene rubber (ABS) nanocomposites. *Chemical Engineering Journal* **281**, 730-738 (2015).

101. Steele A, Nayak BK, Davis A, Gupta MC, Loth E. Linear abrasion of a titanium superhydrophobic surface prepared by ultrafast laser microtexturing. *Journal of Micromechanics and Microengineering* **23**, 115012 (2013).

102. Zhu X*, et al.* Robust superhydrophobic surfaces with mechanical durability and easy repairability. *Journal of Materials Chemistry* **21**, 15793-15797 (2011).

103. Xiang T, *et al.* Fabrication of inherent anticorrosion superhydrophobic surfaces on metals. *ACS Sustainable Chemistry & Engineering* **6**, 5598-5606 (2018).

104. Golovin K, Boban M, Mabry JM, Tuteja A. Designing self-healing superhydrophobic surfaces with exceptional mechanical durability. *ACS applied materials & interfaces* **9**, 11212-11223 (2017).

105. Zhang Z, Ge B, Men X, Li Y. Mechanically durable, superhydrophobic coatings prepared by dual-layer method for anti-corrosion and self-cleaning. *Colloids and Surfaces A: Physicochemical and Engineering Aspects* **490**, 182-188 (2016).

106. Yamauchi Y, Tenjimbayashi M, Samitsu S, Naito M. Durable and flexible superhydrophobic materials: abrasion/scratching/slicing/droplet impacting/bending/twisting-tolerant composite with porcupinefish-like structure. *ACS applied materials & interfaces* **11**, 32381-32389 (2019).

107. Wang R, Jakhar K, Ahmed S, Antao DS. Elucidating the Mechanism of Condensation-Mediated Degradation of Organofunctional Silane Self-Assembled Monolayer Coatings. *ACS Applied Materials & Interfaces* **13**, 34923-34934 (2021).

108. Zhang L, Guo Z, Sarma J, Dai X. Passive removal of highly wetting liquids and ice on quasi-liquid surfaces. *ACS Applied Materials & Interfaces* **12**, 20084-20095 (2020).

109. Monga D, Guo Z, Shan L, Taba SA, Sarma J, Dai X. Quasi-Liquid Surfaces for Sustainable High-Performance Steam Condensation. *ACS Applied Materials & Interfaces*, (2022).

110. Halvey AK, *et al.* Rapid and Robust Surface Treatment for Simultaneous Solid and Liquid Repellency. *ACS Applied Materials & Interfaces* **13**, 53171-53180 (2021).

111. Zhang P, Lv F, Askounis A, Orejon D, Shen B. Role of impregnated lubricant in enhancing thermosyphon performance. *International Journal of Heat and Mass Transfer* **109**, 1229-1238 (2017).

112. Wilke KL, *et al.* Polymer Infused Porous Surfaces for Robust, Thermally Conductive, Self-Healing Coatings for Dropwise Condensation. *ACS Nano* **14**, 14878-14886 (2020).

113. Tech V. VaporTech PVD Coating Machines.).

114. Sullivan JP, Friedmann TA, Baca AG. Stress relaxation and thermal evolution of film properties in amorphous carbon. *Journal of Electronic Materials* **26**, 1021-1029 (1997).

115. Ferrari AC, Kleinsorge B, Morrison NA, Hart A, Stolojan V, Robertson J. Stress reduction and bond stability during thermal annealing of tetrahedral amorphous carbon. *Journal of Applied Physics* **85**, 7191-7197 (1999).

116. Hübsch HDaH. Applying low-friction wear-resistant thin solid films by physical vapour deposition. *Philips Tech Rev 4* **41**, 186-197 (1983/84).

117. K. Bewilogua CVC, C. Specht , J. Schroder , R. Wittorf , Grischke M. Effect of target material on deposition and properties of metal-containing DLC Me-DLC coatings. *Surface and Coatings Technology* **132**, 275-283 (2000).

118. Grischke M, Hieke A, Morgenweck F, Dimigen H. Variation of the wettability of DLC-coatings by network modification using silicon and oxygen. *Diamond and Related Materials* **7**, 454-458 (1998).

119. E. Dekempeneera KVA, K. Vercammen, J. Meneve, . Neerinck, S. Eufinger, W. Pappaert, M. Sercu, J. Smeets. Abrasion resistant low friction diamond-like multilayers. *Surface and Coatings Technology* **142**, 669-673 (2001).

120. Bertran E, Martinez E, Viera G, Farjas J, Roura P. Mechanical properties of nanometric structures of Si/SiC, C/SiC and C/SiN produced by PECVD. *Diamond and Related Materials* **10**, 1115-1120 (2001).

121. Chouquet C, Ducros C, Barrat S, Billard A, Sanchette F. Mechanical properties of a-C:H/Si-containing a-C:H multilayered coatings grown by LF-PECVD. *Surface and Coatings Technology* **203**, 745-749 (2008).

122. Robertson J. Diamond-Like Carbon Films, Properties and Applications. In: *Comprehensive Hard Materials*) (2014).

123. W. Augustin JZ, I. Bialuch, T. Geddert, S. Scholl. Modified DLC Coating for the Mitigation of Scaling on Heat Exchanger Surfaces In: *Proceedings of Fifth International Conference on Enchanced, Compact and Ultra-Compact Heat Exchangers: Sciecne, Engineering and Technology*) (2005).

124. Sett S, Sokalski P, Mehta M, Rabbi KF, Gunay A, Miljkovic N. Transient pulse condensation. *Applied Physics Letters* **117**, (2020).

125. Sett S*, et al.* Stable Dropwise Condensation of Ethanol and Hexane on Rationally Designed Ultrascalable Nanostructured Lubricant-Infused Surfaces. *Nano Lett* **19**, 5287-5296 (2019).

126. Rose J. Dropwise condensation theory and experiment: a review. *Proceedings of the Institution of Mechanical Engineers, Part A: Journal of Power and Energy* **216**, 115-128 (2002).

127. Bergman TL, Incropera FP, DeWitt DP, Lavine AS. *Fundamentals of heat and mass transfer*. John Wiley & Sons (2011).

128. Petukhov B. An investigation of heat transfer to fluids flowing in pipes under supercritical conditions. In: *International Developments in Heat Transfer: Proceedings of the 1961-62 Heat Transfer Conference, August 28-September 1, 1961, University of Colorado, Boulder, Colorado USA, January 8-12, 1962, Continued discussions, Central Hall Lecture Theatre, Westminster, London, England*). The American Society of Mechanical Engineers (1963).

129. Miljkovic N, Enright R, Wang EN. Modeling and Optimization of Superhydrophobic Condensation. *Journal of Heat Transfer* **135**, (2013).

130. Kim S, Kim KJ. Dropwise Condensation Modeling Suitable for Superhydrophobic Surfaces. *Journal of Heat Transfer* **133**, (2011).

131. Miljkovic N, Enright R, Wang EN. Effect of Droplet Morphology on Growth Dynamics and Heat Transfer during Condensation on Superhydrophobic Nanostructured Surfaces. *ACS Nano* **6**, 1776-1785 (2012).

132. Scharge RW. A Theoretical Study of Interphase Mass Transfer.). Columbia University, New York (1953).

133. Kim S, Kim KJ. Dropwise Condensation Modeling Suitable for Superhydrophobic Surfaces. *Journal of Heat Transfer* **133**, 081502 (2011).

134. Le Fevre EJ, Rose JW. A Theory of Heat Transfer by Dropwise Condensation. In: *Proceedings of the Third International Heat Transfer Conference*). ASME (1966).

135. Miljkovic N, *et al.* Jumping-droplet-enhanced condensation on scalable superhydrophobic nanostructured surfaces. *Nano letters* **13**, 179-187 (2012).

136. Paxson AT, Yague JL, Gleason KK, Varanasi KK. Stable Dropwise Condensation for Enhancing Heat Transfer via the Initiated Chemical Vapor Deposition (iCVD) of Grafted Polymer Films. *Adv Mater* **26**, 418-423 (2014).

137. Wang RS, Jakhar K, Ahmed S, Antao DS. Elucidating the Mechanism of Condensation-Mediated Degradation of Organofunctional Silane Self-Assembled Monolayer Coatings. *Acs Appl Mater Inter* **13**, 34923-34934 (2021).

138. Wang RS, Guo JH, Muckleroy EA, Antao DS. Robust silane self-assembled monolayer coatings on plasma-engineered copper surfaces promoting dropwise condensation. *International Journal of Heat and Mass Transfer* **194**, (2022).

139. Yan X, *et al.* Atmosphere-Mediated Superhydrophobicity of Rationally Designed Micro/Nanostructured Surfaces. *ACS Nano* **13**, 4160-4173 (2019).

140. Wang R, Guo J, Muckleroy EA, Antao DS. Robust Silane Self-Assembled Monolayer Coatings on Plasma-Engineered Copper Surfaces Promoting Dropwise Condensation. *International Journal of Heat and Mass Transfer* **194**, (2022).

141. Wang R, Jakhar K, Ahmed S, Antao DS. Elucidating the Mechanism of Condensation-Mediated Degradation of Organofunctional Silane Self-Assembled Monolayer Coatings. *ACS Appl Mater Interfaces* **13**, 34923-34934 (2021).

142. van Doornmalen JP, Kopinga K. Measuring non-condensable gases in steam. *Rev Sci Instrum* **84**, 115106 (2013).

143. Mousa MH, Gunay AA, Orejon D, Khodakarami S, Nawaz K, Miljkovic N. Gas-Phase Temperature Mapping of Evaporating Microdroplets. *ACS Appl Mater Interfaces* **13**, 15925-15938 (2021).

144. Li J-D, Saraireh M, Thorpe G. Condensation of vapor in the presence of non-condensable gas in condensers. *International Journal of Heat and Mass Transfer* **54**, 4078-4089 (2011).

145. Ma J, Cha H, Kim M-K, Cahill DG, Miljkovic N. Condensation Induced Delamination of Nanoscale Hydrophobic Films. *Advanced Functional Materials* **29**, 1905222 (2019).

146. Zhou Yang Y-ZW, Yi-Fan Ye, Mao-Gang Gong, Xiao-Liang Xu. A simple way to fabricate an aluminum sheet with superhydrophobic and self-cleaning properties. *Chinese Physics B* **21**, (2012).

147. Gelest. HTMS.).

148. LUMIFLON. Life Cycle Cost Analysis: LUMIFLON vs. Conventional Coatings.).

149. McGinley. How Much of Each Energy Source Does It Take to Power Your Home (2023).

150. Spencer E. Specifying Steam Surface Condensers.). Graham Mfg.Co., Inc.

151. Latchford I, Riposan A, Kudriavtsev V, Bluck T, Smith C. Cost of Ownership Analysis for a High Productivity Thin Film PVD System. In: *Society of Vacuum Coaters 57th (2014) Annual Technical Conference Proceedings*) (2015).

152. Wayne D. How Much Does PVD Coating Cost?). VaporTech (2022).

153. Mattox DM. *Handbook of Physical Vapor Deposition (PVD) Processing Film Formation, Adhesion, Surface Preparation and Contamination Control*. Knovel: Norwich (1998).

154. Insights FB. Physical Vapor Deposition Market) (2021).

155. Selvakumar N, Barshilia HC. Review of physical vapor deposited (PVD) spectrally selective coatings for mid- and high-temperature solar thermal applications. *Solar Energy Materials and Solar Cells* **98**, 1-23 (2012).

156. Power CS. Technology roadmap concentrating solar power. *Current* **5**, 1-52 (2010).

157. NREL. http:www.nrel.gov csp solarpaces.).

158. Craig S. Turchi MB, Devon Kesseli, Parthiv Kurup, Mark Mehos, Ty Neises, Prashant Sharan, Michael Wagner, and Timothy Wendelin. CSP Systems Analysis - Final Project Report.) (2019).

159. Silva FJG, Fernandes AJS, Costa FM, Teixeira V, Baptista APM, Pereira E. Tribological behaviour of CVD diamond films on steel substrates. *Wear* **255**, 846-853 (2003).

160. Stockwell CE, *et al.* Volatile organic compound emissions from solvent- and water-borne coatings – compositional differences and tracer compound identifications. *Atmospheric Chemistry and Physics* **21**, 6005-6022 (2021).

161. Panieri E, Baralic K, Djukic-Cosic D, Buha Djordjevic A, Saso L. PFAS Molecules: A Major Concern for the Human Health and the Environment. *Toxics* **10**, (2022).

162. Azhdarzadeh M, *et al.* An Atomizer to Generate Monodisperse Droplets from High Vapor Pressure Liquids. *Atomization and Sprays* **26**, 121-134 (2016).

163. Baldelli A, Ou J, Li W, Amirfazli A. Spray-On Nanocomposite Coatings: Wettability and Conductivity. *Langmuir* **36**, 11393-11410 (2020).
