# Supplementary material for: Ultra-resilient multi-layer fluorinated diamond like carbon hydrophobic surfaces
Source: Nat Commun. 2023 Aug 14;14:4902. doi: 10.1038/s41467-023-40229-6 (PMC10425355; doi:10.1038/s41467-023-40229-6)
Supplement: Supplementary file 2 — Description of Additional Supplementary Files Document [file 41467_2023_40229_MOESM2_ESM.pdf]

Description of Additional Supplementary Files

Supplementary Movie 1. Steady state condensation of water vapor on an F-DLC coated Cu tube. The vapor pressure inside the environmental chamber was ≈ 4 ± 0.15 kPa. The video was captured with Canon EOS 7D camera at 30 frames per second (fps) and are played back at 30 fps. The cooling water flow rate was ≈ 8 ± 0.2 L/min inside the horizontal tube.
